# Supplementary material for: Smoother: a unified and modular framework for incorporating structural dependency in spatial omics data
Source: Genome Biol. 2023 Dec 18;24:291. doi: 10.1186/s13059-023-03138-x (PMC10726548; doi:10.1186/s13059-023-03138-x)
Supplement: Supplementary file 1 — Additional file 1: Fig. S1. Spatially adjacent spots share similar profiles, a common property of spatial omics data observed across biological systems and technologies. The figure demonstrates the distributions of pairwise cosine similarity between neighboring spots in spatial omics data. Similarity is calculated based on the first 20 PCs of log-normalized gene counts (for transcriptomics data) or gene activity scores (for epigenomics data). All transcriptomics data were preprocessed following the standard Scanpy workflow (1) by SODB (2). Left in each panel: Cumulative distribution of pairwise similarity between k-nearest physically adjacent spots. Red line shows the observed distribution whereas blue line shows the null distribution of random shuffled spots. Right in each panel: Variability in pairwise similarity as a function of neighbor distance. For each neighborhood degree level k (x-axis), pairwise similarities were calculated between the k-th nearest neighbors (self-similarity of 1 when k=0). The light gray area indicates the 10%-90% range, dark gray the 25%-75% range, and the red line the median, respectively. Datasets inspected: (a) 10x Visium human brain dorsolateral prefrontal cortex (DLPFC) (3). (b) 10x Visium human pancreatic ductal adenocarcinoma (PDAC) (4). (c) Slide-seqV2 mouse hippocampus (5). (d) Slide-seqV2 human melanoma brain metastasis (MBM) (6). (e) Stereo-seq axolotl brain (segmented and binned at single-cell resolution) (7). (f) Stereo-seq human colorectal cancer (CRC, binned into 50 µm x 50 µm spots) (8). (g) Spatial-CUT&Tag mouse embryo, H3K4me3 20µm (9). (h) Spatial-CUT&Tag mouse embryo, H3K4me3 50µm (9). (i) Shuffled Stereo-seq single-cell resolution data. Per contamination rate p, each spot contains 1-p of its original RNA counts and p of RNA counts from its adjacent neighbors. Fig. S2. Construction of boundary-preserving spatial priors. (a) Schematic illustration of the process for constructing spatial weights and covariance matrices. [file 13059_2023_3138_MOESM1_ESM.pdf]

Fig. S1

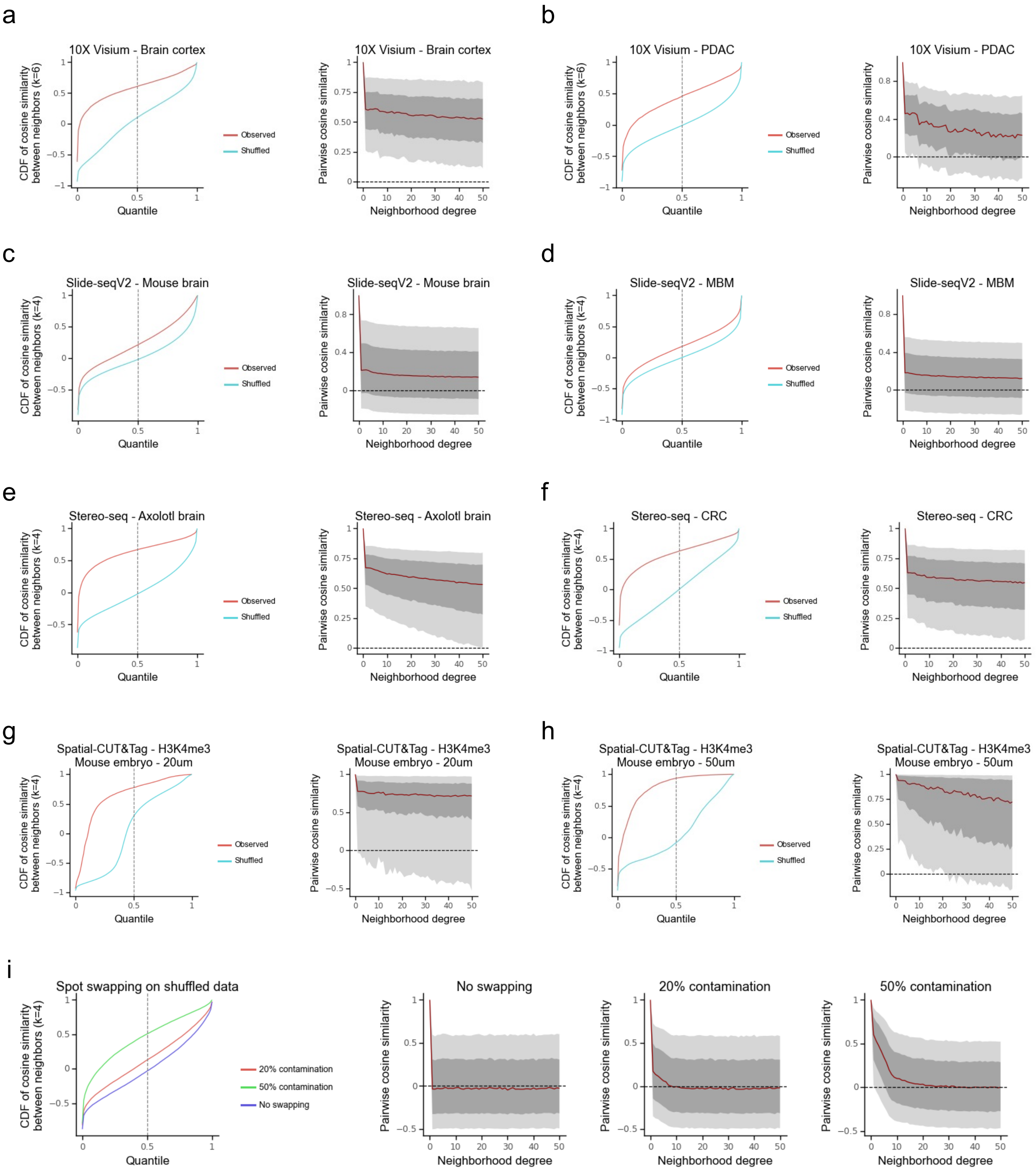

Fig. S1: Spatially adjacent spots share similar profiles, a common property of spatial omics data observed across biological systems and technologies.

The figure demonstrates the distributions of pairwise cosine similarity between neighboring spots in spatial omics data. Similarity is calculated based on the first 20 PCs of log-normalized gene counts (for transcriptomics data) or gene activity scores (for epigenomics data). All transcriptomics data were preprocessed following the standard Scanpy workflow(1) by SODB(2). **Left in each panel:** Cumulative distribution of pairwise similarity between k-nearest physically adjacent spots. Red line shows the observed distribution whereas blue line shows the null distribution of random shuffled spots. **Right in each panel:** Variability in pairwise similarity as a function of neighbor distance. For each neighborhood degree level k (x-axis), pairwise similarities were calculated between the k-th nearest neighbors (self-similarity of 1 when k=0). The light gray area indicates the 10%-90% range, dark gray the 25%-75% range, and the red line the median, respectively. **Datasets inspected:** (a) 10x Visium human brain dorsolateral prefrontal cortex (DLPFC)(3). (b) 10x Visium human pancreatic ductal adenocarcinoma (PDAC)(4). (c) Slide-seqV2 mouse hippocampus(5). (d) Slide-seqV2 human melanoma brain metastasis (MBM)(6). (e) Stereo-seq axolotl brain (segmented and binned at single-cell resolution)(7). (f) Stereo-seq human colorectal cancer (CRC, binned into 50 μm x 50 μm spots)(8). (g) Spatial-CUT&Tag mouse embryo, H3K4me3 20μm(9). (h) Spatial-CUT&Tag mouse embryo, H3K4me3 50μm(9). (i) Shuffled Stereo-seq single-cell resolution data. Per contamination rate p, each spot contains 1-p of its original RNA counts and p of RNA counts from its adjacent neighbors.

Fig. S2

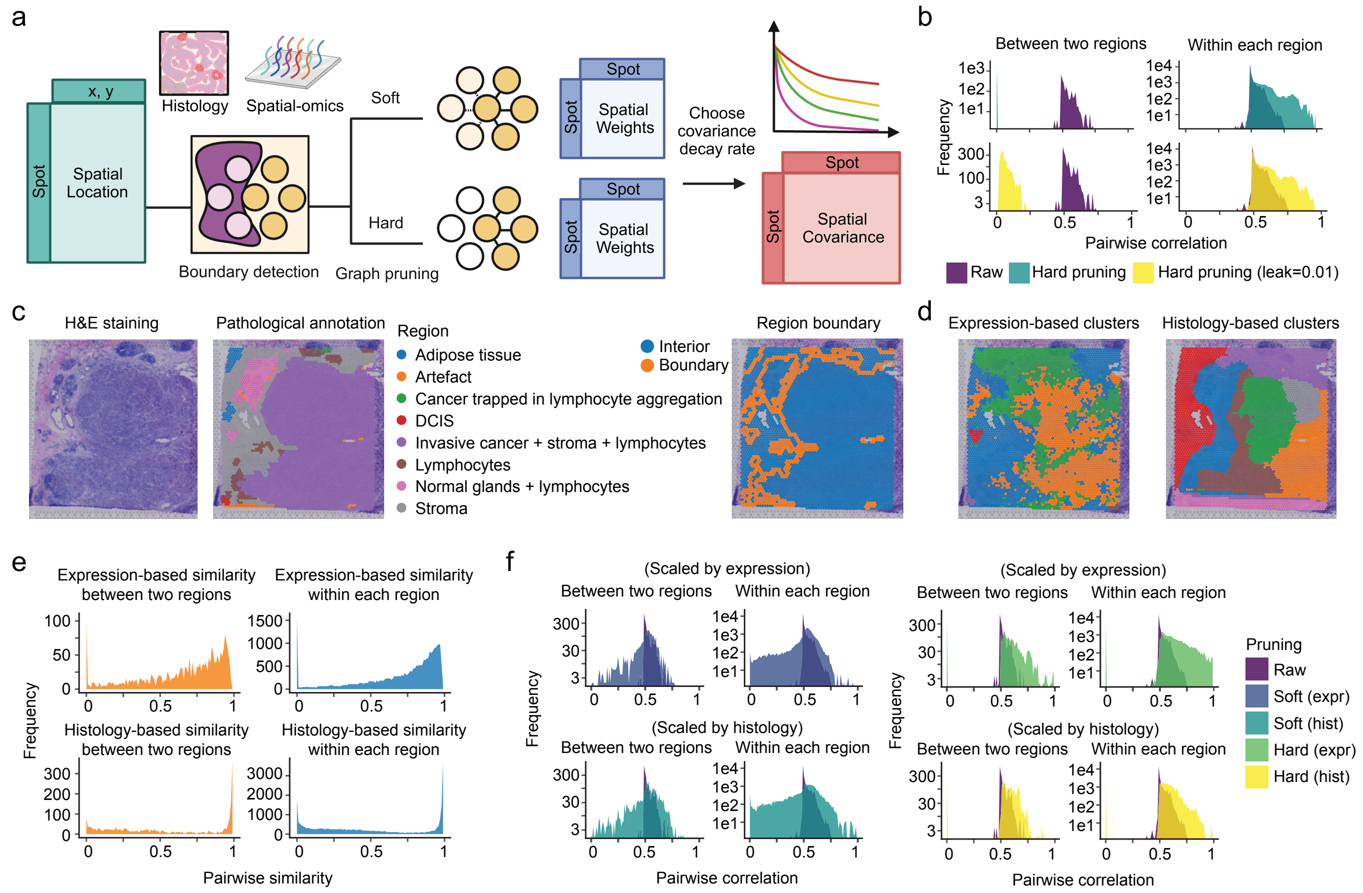

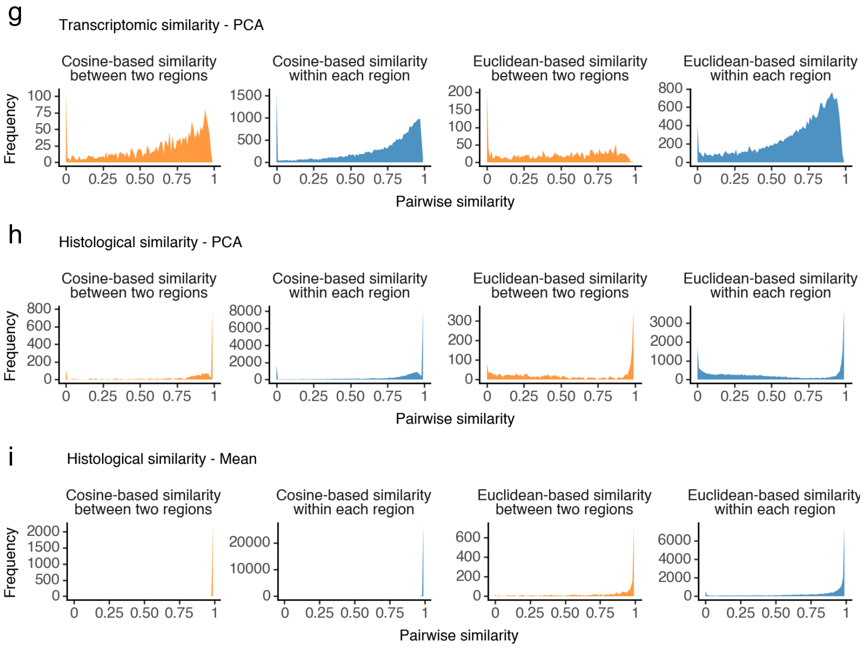

Fig. S2: Construction of boundary-preserving spatial priors.
(a) Schematic illustration of the process for constructing spatial weights and covariance matrices. Smoother first builds a distance-based spatial neighborhood graph, then prunes the graph using histology or other features to encode boundary information and inhibit undesired cross-region interactions. (b) The impact of hard pruning on the covariance structure. Given the regional membership annotation, hard pruning sets the weights between adjacent spots of different regions to zero (teal, top) or a nominal value (leak=0.01, yellow, bottom), generating distinctive correlation distributions for boundary and interior pairs of spots. (c) An example section (1160920F) from the human breast cancer datasets(10). Left to right: H&E staining, manual pathological annotation, and depiction of boundary and interior spots as defined by the pathological regions. (d) Unsupervised clustering results of the same slide in (c). Spots were clustered based on gene expression (left), or image-based features extracted from the H&E staining image (right).
(e) The distribution of pairwise feature similarities between boundary pairs from two regions (orange) and interior pairs from the same region (blue), as defined in (c) by the pathological annotation. (f) The distribution of pairwise correlations imposed by the resulting spatial priors after graph pruning. Regions were again defined by the pathological annotation in (c). Left: Soft scaling using expression- (top) and histology-based similarities (bottom). Right: Hard pruning using cluster memberships from expression (top) and histology (bottom) as defined in (d). (g-i) Distributions of pairwise feature similarities as shown in (e) but calculated with different preprocessing steps: (g) Transcriptomic similarities are calculated based on the log-normalized expression data of the top 2000 highly variable genes. PCA with zero-centering and scaling was applied to reduce the feature to the first 10 PCs. Left two panels: Cosine similarity. Right two panels: Euclidean similarity as converted from the Euclidean distance using an exponential kernel of bandwidth 0.01. (h) Similar to (g), except the feature is the concatenated RGB values of pixels of the H&E staining covered by the spot. PCA with zero-centering and scaling was applied to reduce the feature to the first 10 PCs. (i) Similar to (h), except the feature is the RGB values averaged over pixels of the H&E staining covered by the spot.

Fig. S3

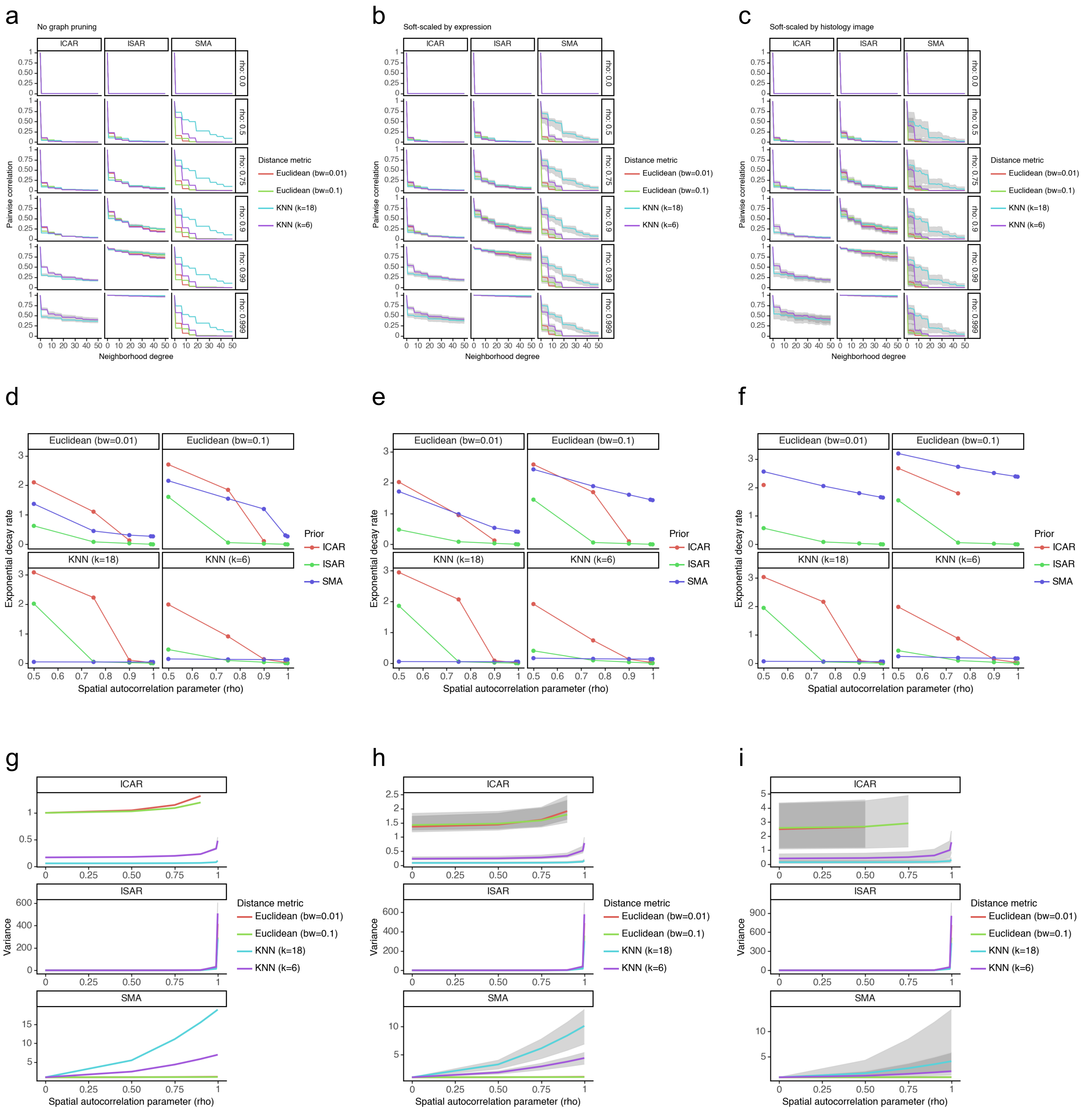

Fig. S3: Covariance structure analysis under different spatial random processes and autocorrelation parameters. (a-c) Distribution of pairwise correlation between the k-th nearest neighbors, as specified by the spatial stochastic process (columns) and the autocorrelation parameter (rows) ‘rho’. Intuitively, the autocorrelation parameter ‘rho’ can be viewed as the proportion of information that comes from the neighbors. The distributions are shown with colored lines and the associated gray areas indicating the median and 25%-75% range of correlation, respectively, over all neighboring pairs of the same neighborhood degree k (self-correlation of 1 when k=0). Color denotes different strategies to construct the spatial weights matrix. The spatial weights in the neighborhood graph are further scaled by expression similarity in (b), and by histology similarity in (c). Estimated exponential decay rates of median correlation are depicted in (d-f), and distributions of per-spot variances are shown in (g-i).

Fig. S4

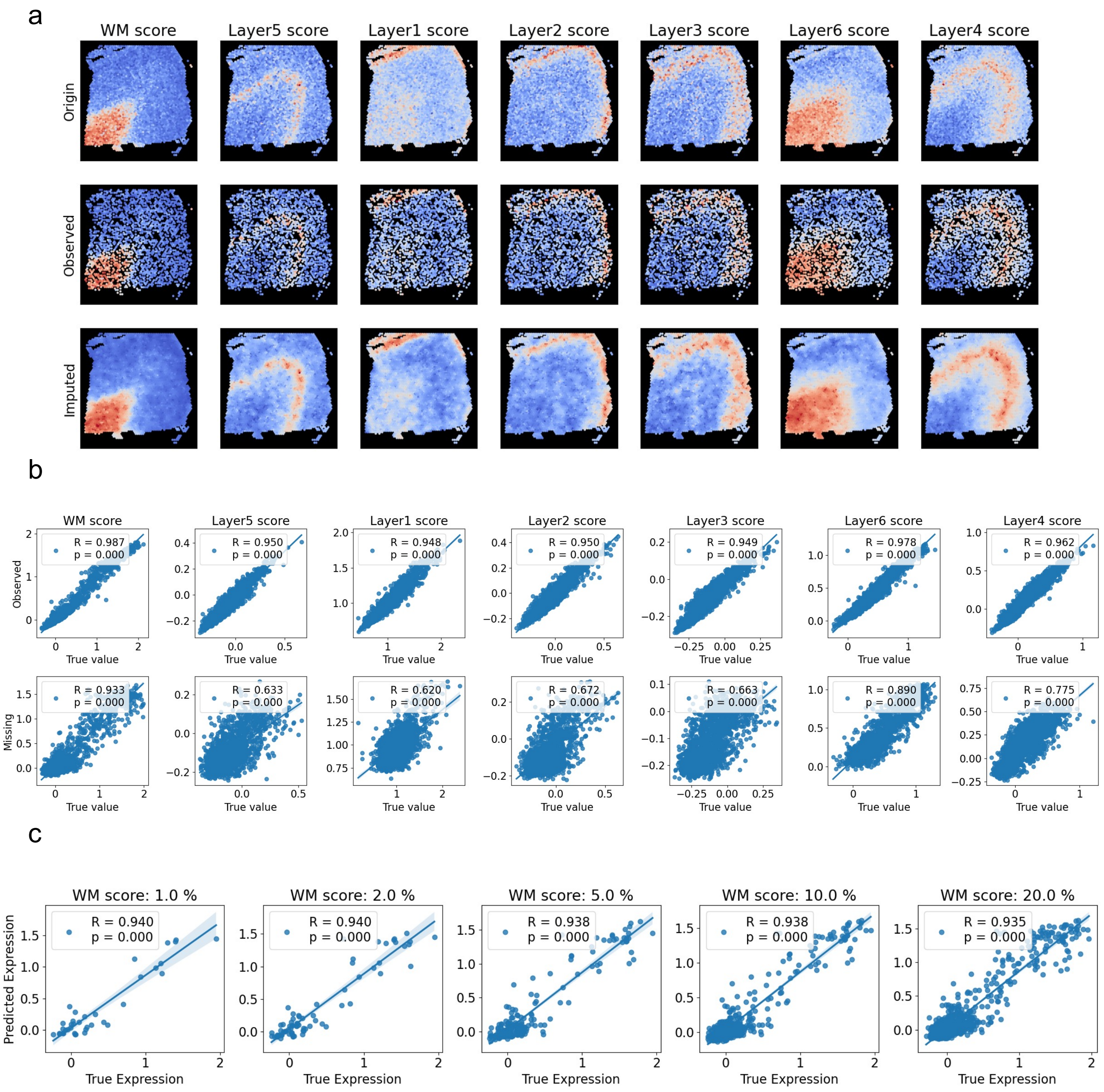

Fig. S4: Application of Smoother for gene signature score imputation and smoothing in the DLPFC dataset (151673)(3). Gene signature scores were defined per region using 'scanpy.tl.score_genes' as the weighted scaled average of top 20 marker genes of that region (adjusted for gene background). (a) Recovery of spatial patterns of signature scores through Smoother-guided data imputation. 2000 out of total 3639 spots are supplied as inputs (second row) to infer the score in all spots (third row). (b) Scatter plots of the imputed score against the observed score in input spots (top) and masked-out spots (bottom). (c) Imputation performances of the white matter (WM) score at masked-out locations as a function of mask-out rate.

Fig. S5

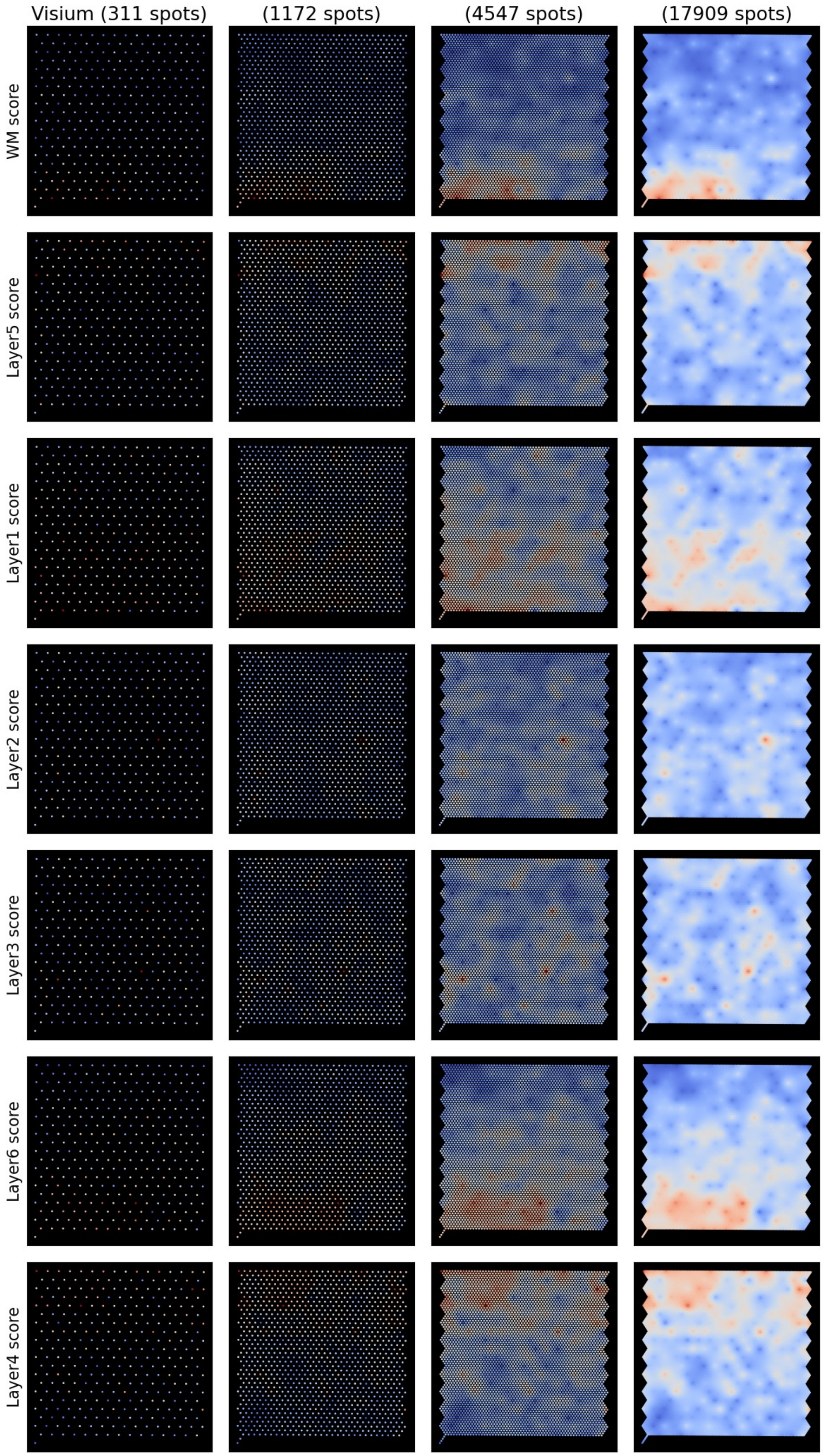

Fig. S5: Resolution enhancement of gene signature scores in the DLPFC dataset (151673)(3).
The figure depicts the enhancing of the spatial resolution of gene signature scores in the DLPFC slide 151673. The original 10x Visium slide was cropped to a smaller size (first column) before running enhancement to higher resolutions. Gene signature scores were defined per region using 'scanpy.tl.score_genes' as the weighted scaled average of top 20 marker genes of that region (adjusted for gene background).

Fig. S6

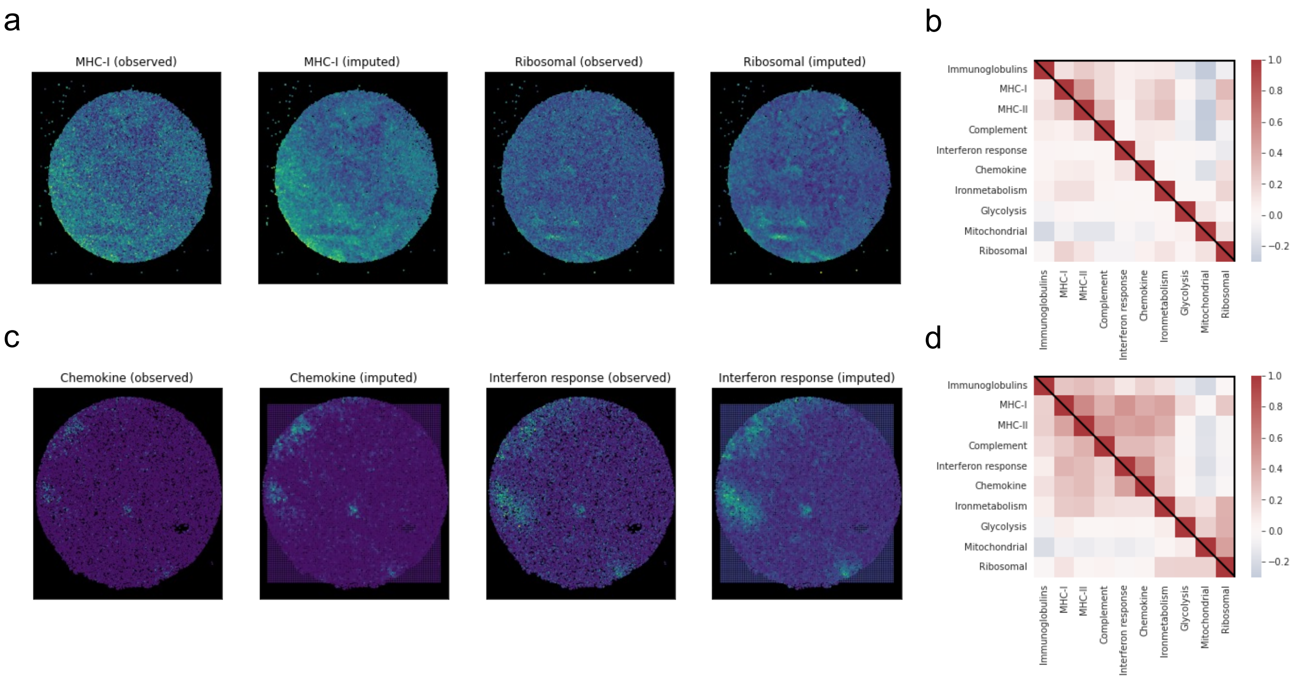

Fig. S6: Application of Smoother for smoothing and enhancing functional activity scores in the Slide-seqV2 MBM data(6).
The figure demonstrates the imputation (a, b) and resolution enhancement (c, d) of functional signature scores in a Slide-seqV2 melanoma brain metastasis slide (MBM11_rep2, a, b) and an extracranial melanoma metastasis slide (ECM01_rep2, c, d). Functional scores are calculated based on marker gene expression following the original publication. (a) MHC-I and ribosomal scores before and after smoothing. (b) Pairwise correlation between functional signature scores before (lower left) and after (upper right) smoothing. (c) Resolution enhancement of chemokine and interferon response scores before and after enhancement. (d) Pairwise correlation between functional signature scores before (lower left) and after (upper right) enhancement.

Fig. S7

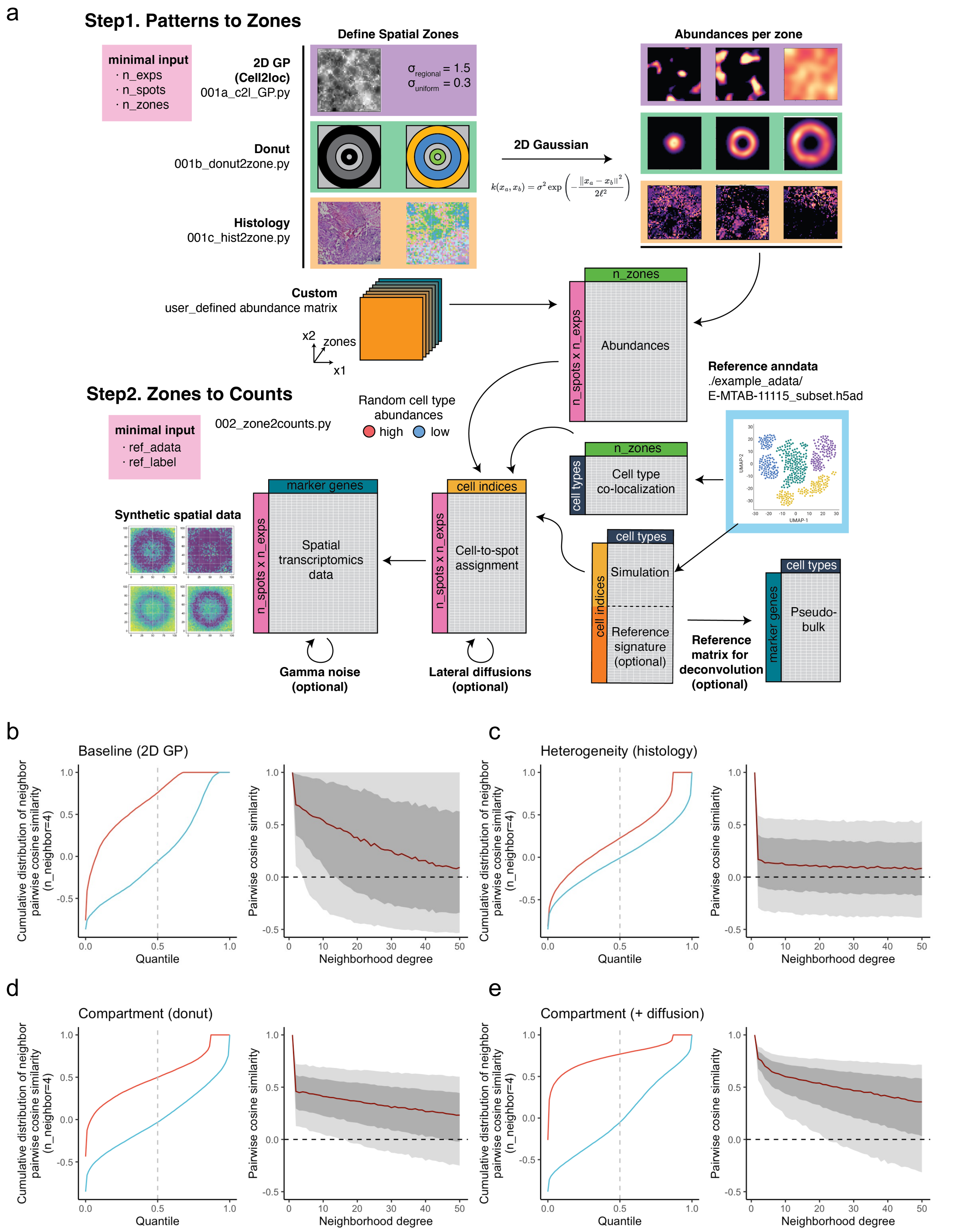

Fig. S7: Simulating spatial transcriptomics data for deconvolution benchmark.
(a) The simulation pipeline, based on cell2location(11) introduces key modifications to assign arbitrary spatial patterns to zones (co-localized cell type groups) and incorporate additional noise (termed lateral diffusion in this paper, also called contamination, spot swapping or bleeding in literature) to account for cell-type-independent dependencies. (b-e) Spatial dependency structures of the simulated data, related to **Fig. S1**. From b to e: a baseline pattern generated by the 2D Gaussian process (cell2location), a heterogeneous pattern specified by a tumor histology image, a pattern with clear doughnut-shaped compartments, and with additional lateral diffusion noise.

Fig. S8

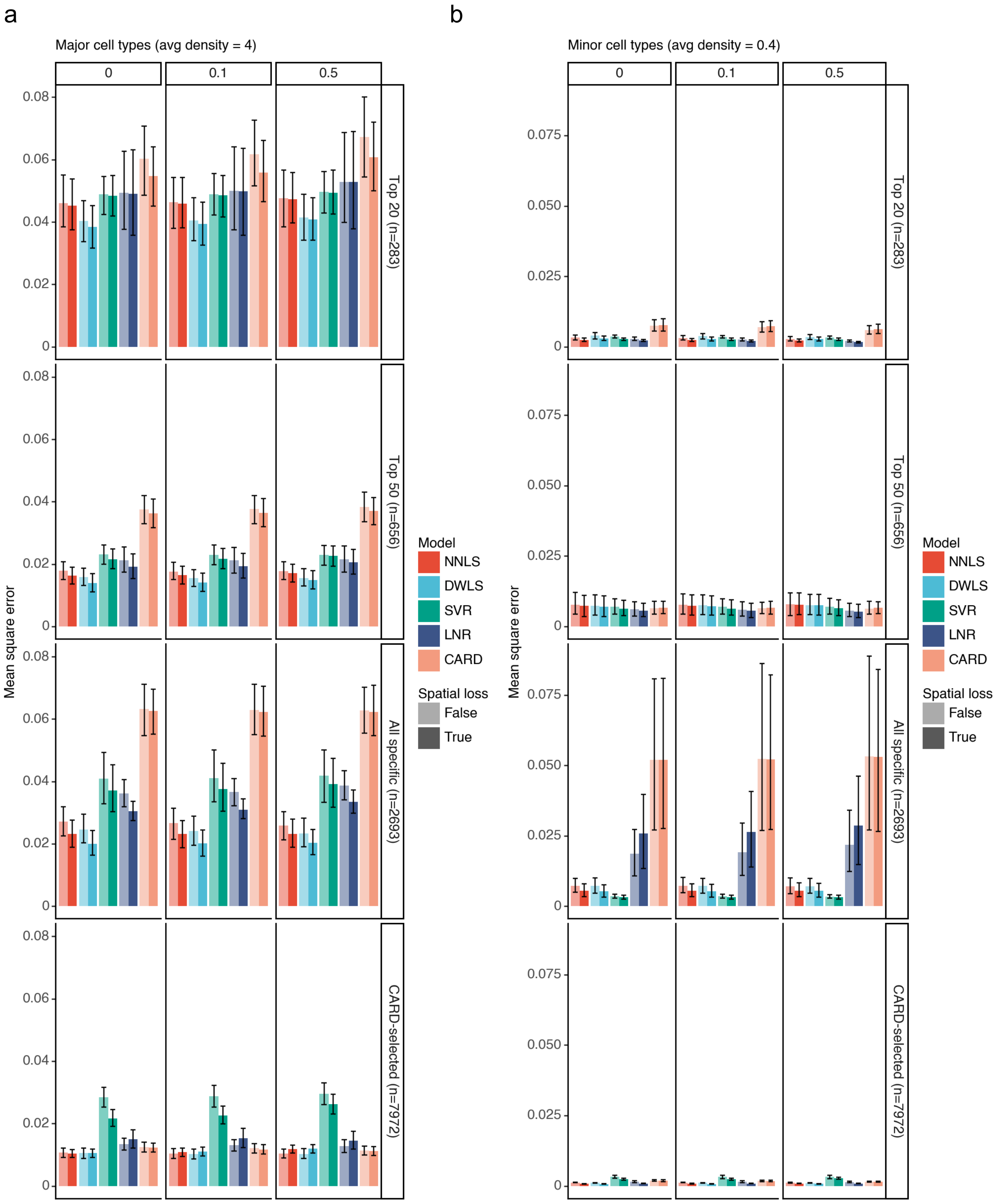

Fig. S8: Evaluation of deconvolution accuracy on simulated data under different scenarios measured by mean square error (the lower the better), related to Fig. 2a-d.

Doughnut-shaped spatial transcriptomics datasets, with varying degree of lateral diffusion (columns, number indicates the proportion of mRNA shared with adjacent spots), were generated from scRNA-seq reference on a 50x50 grid. In each of the ten experiments (replicates), we assigned 5 major cell types with high average abundances (a) and 10 minor cell types with low abundances (b) to overlapping spatial compartments. Mean square error (MSE) was calculated based on the true and estimated cell type proportions (sum to one per spot). Each row represents the scenario where only a subset of genes is informative as deconvolution input. From top to bottom: the union of the top 20 marker genes for each cell type (n=283), the union of top 50 marker genes (n=656), all informative markers genes whose log2(fold change) passes the threshold (by default 1) for one and only one cell type (n=2693), and informative genes selected by the CARD model (n=7972). Error bars denote standard error of the mean over 5 high- or 10 low-density cell types in 10 replicates.

Fig. S9

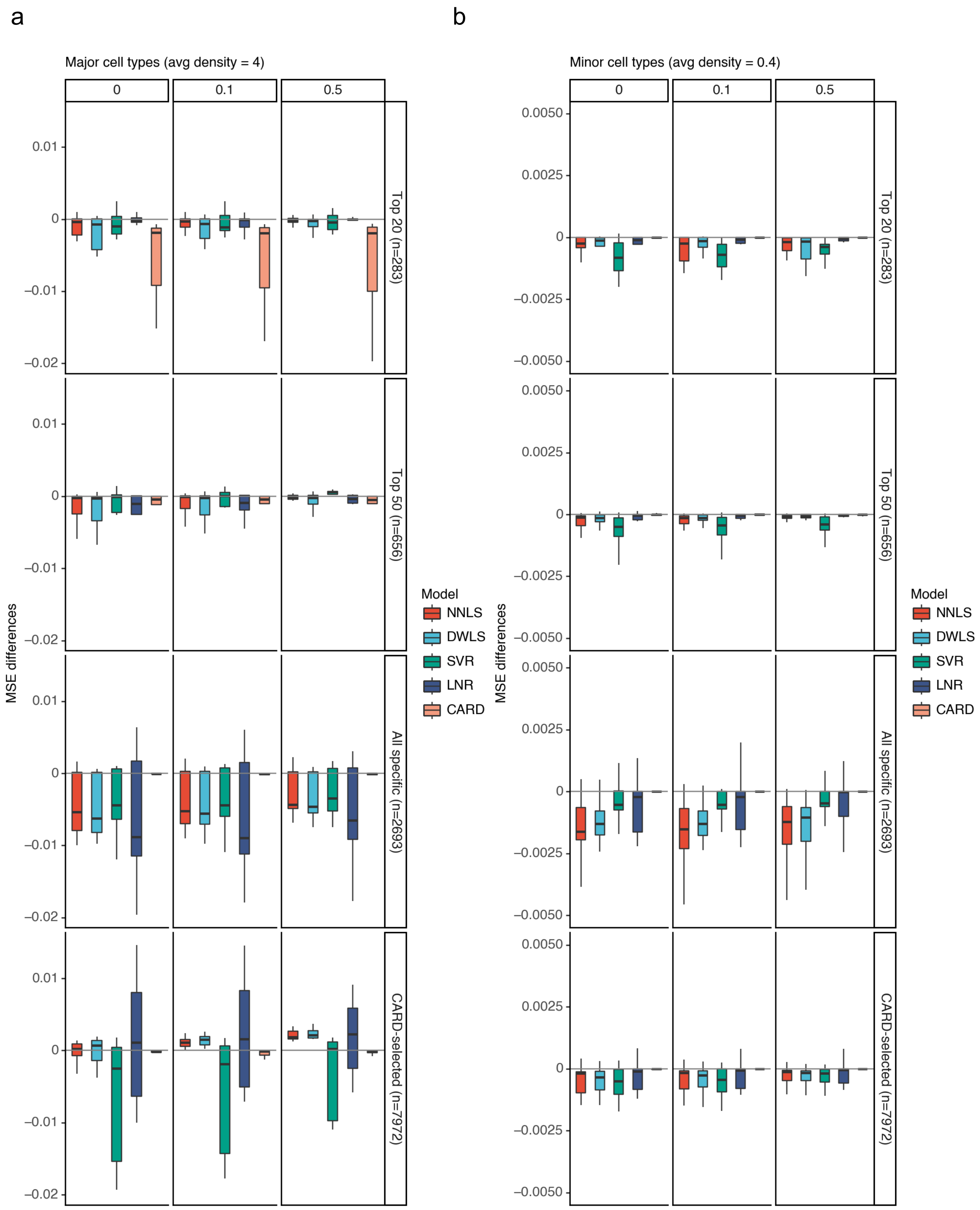

Fig. S9: Performance gained from the spatial regularization under different scenarios, measured by mean square error (the lower the better), related to Fig. S8.

Box plots showing the distribution of performance differences after the spatial regularization is incorporated into deconvolution. Scores were calculated by subtracting the MSE of the non-spatial baseline model from the MSE of a spatially aware model for each cell type in each experiment. Each box plot demonstrates the median and the 25th/75th percentiles of 50 samples for major cell types (a) and 100 for minor cell types (b), as well as the above and below 1.5 times interquartile ranges indicated by the whiskers.

Fig. S10

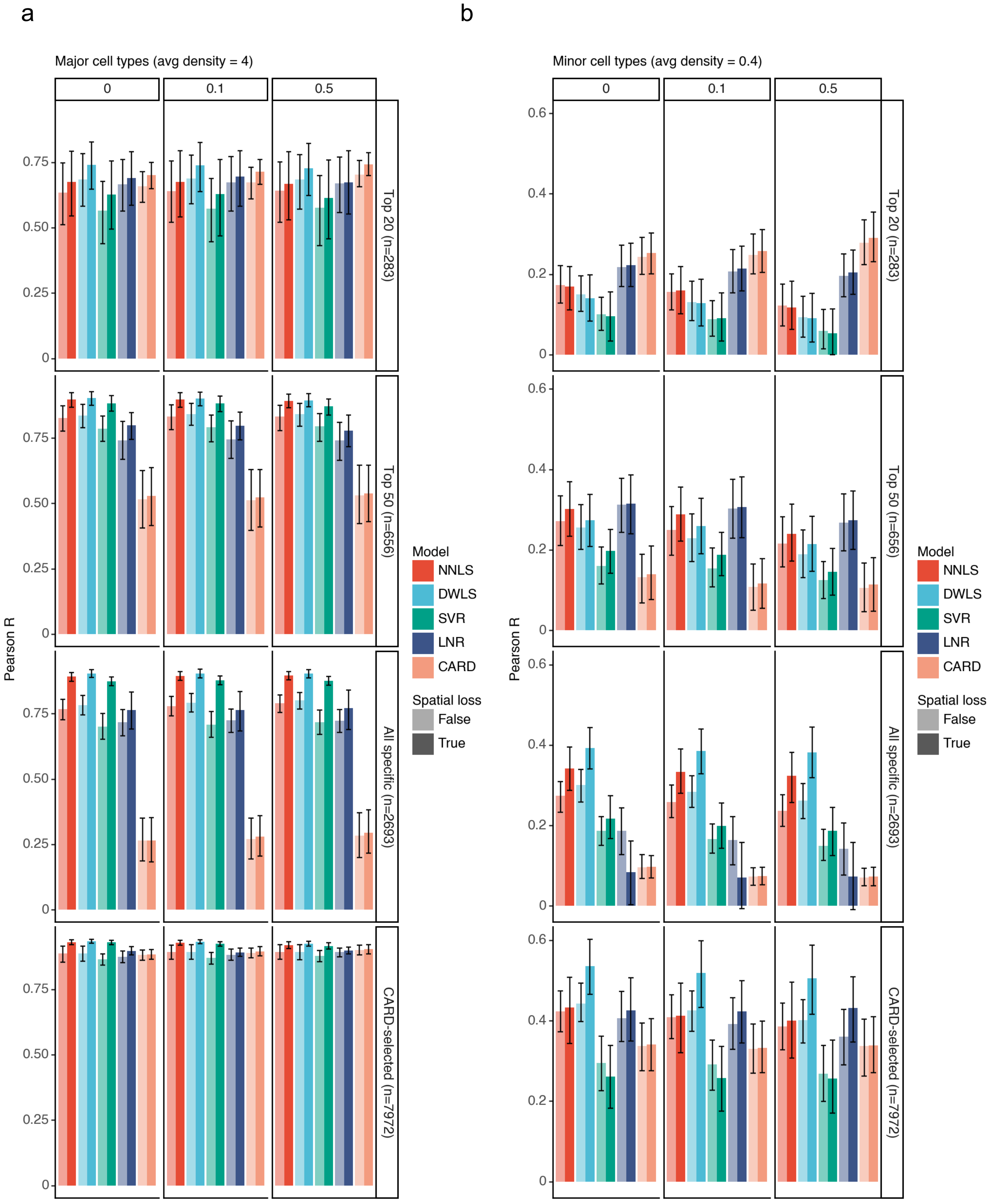

Fig. S10: Evaluation of deconvolution accuracy on simulated data under different scenarios measured by Pearson correlation (the higher the better), related to Fig. 2a-d.
Similar to **Fig. S8** except the performance is measured by the Pearson correlation between the true and estimated cell-type proportions (sum to one per spot).

Fig. S11

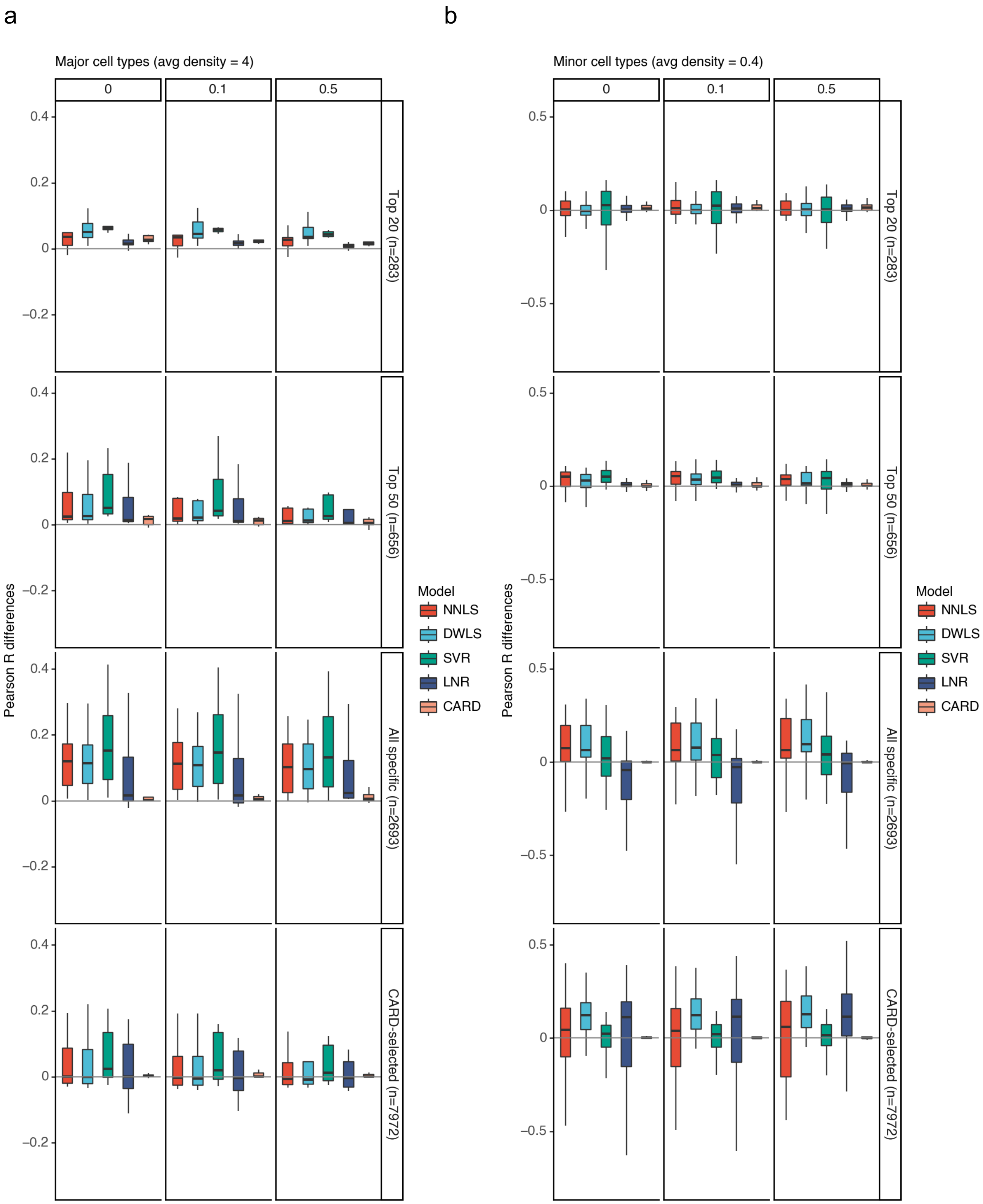

Fig. S11: Performance gained from the spatial regularization under different scenarios, measured by Pearson correlation (the higher the better), related to Fig. S10.
Similar to **Fig. S9** except the performance is measured by the Pearson correlation between the true and estimated cell-type proportions (sum to one per spot).

Fig. S12

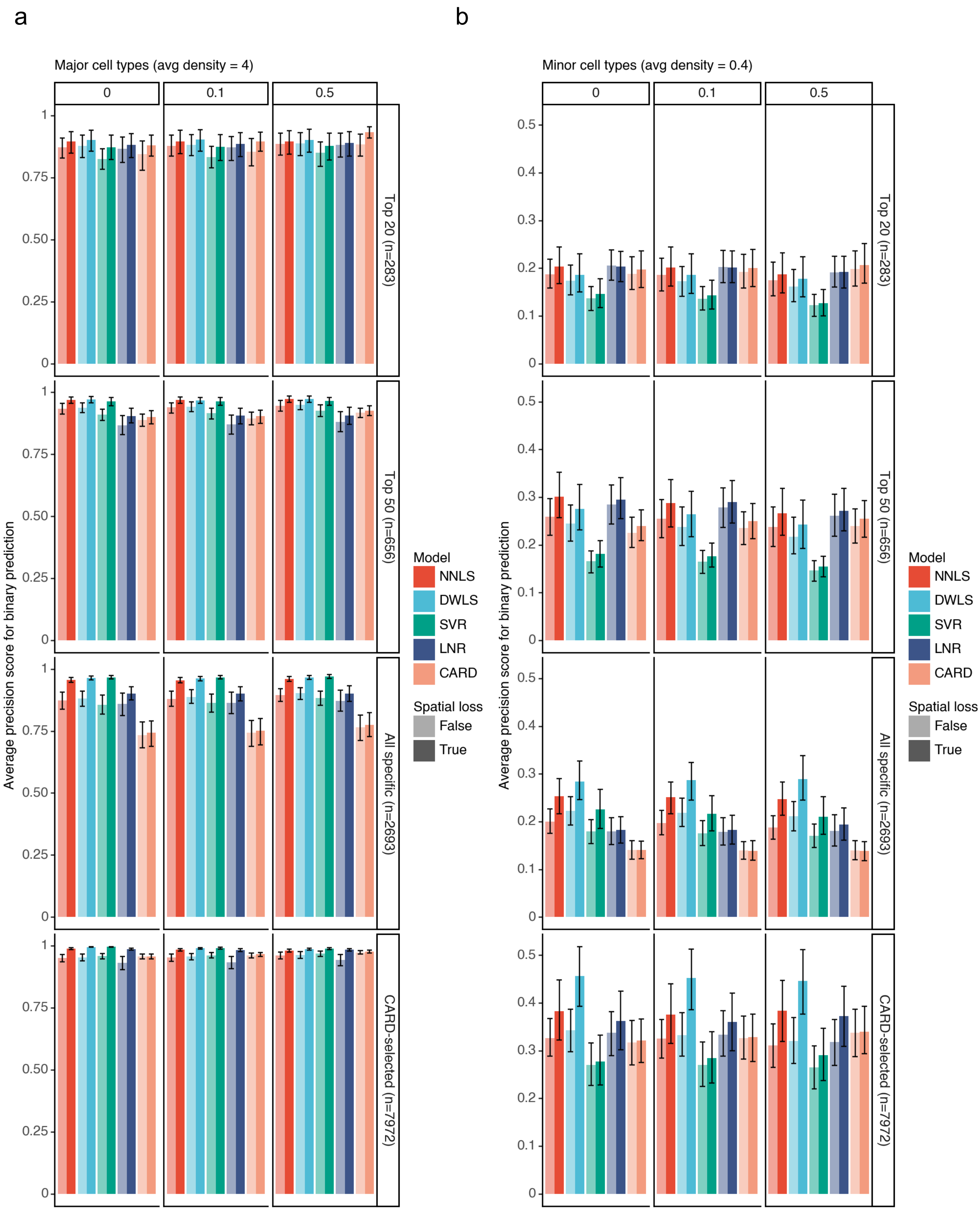

Fig. S12: Evaluation of deconvolution accuracy on simulated data under different scenarios measured by binary prediction accuracy (the higher the better), related to Fig. 2a-d.
Similar to **Fig. S8** except the performance is measured by binary prediction accuracy. Cell types that have a ground truth proportion larger than 0.05 were considered as present at a spot. Average precision scores were calculated for cell types that are at least present in one location in each experiment individually.

Fig. S13

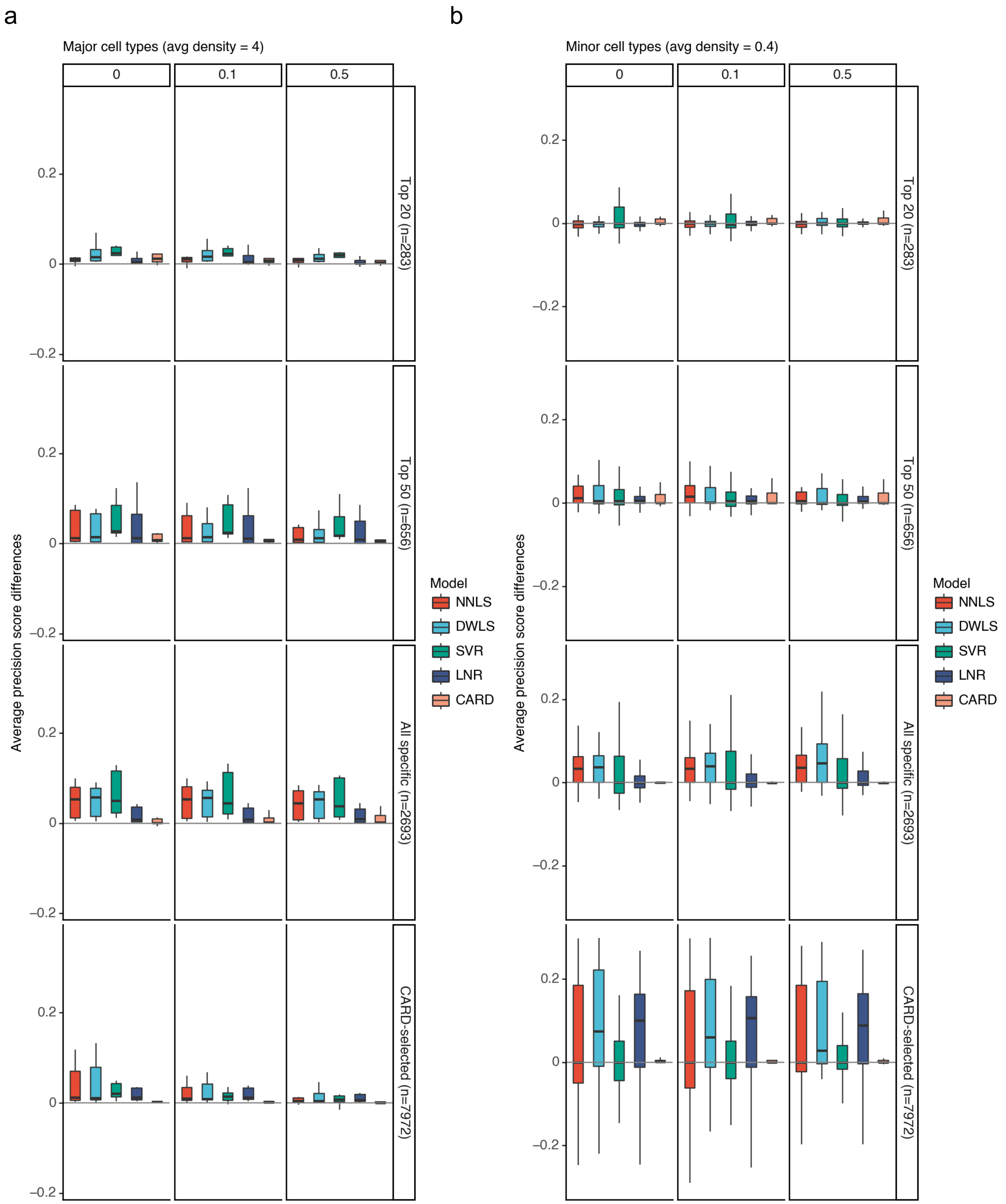

Fig. S13: Performance gained from the spatial regularization under different scenarios, measured by binary prediction accuracy (the higher the better), related to Fig. S12.
Similar to **Fig. S9** except the performance is measured by the average precision score.

Fig. S14

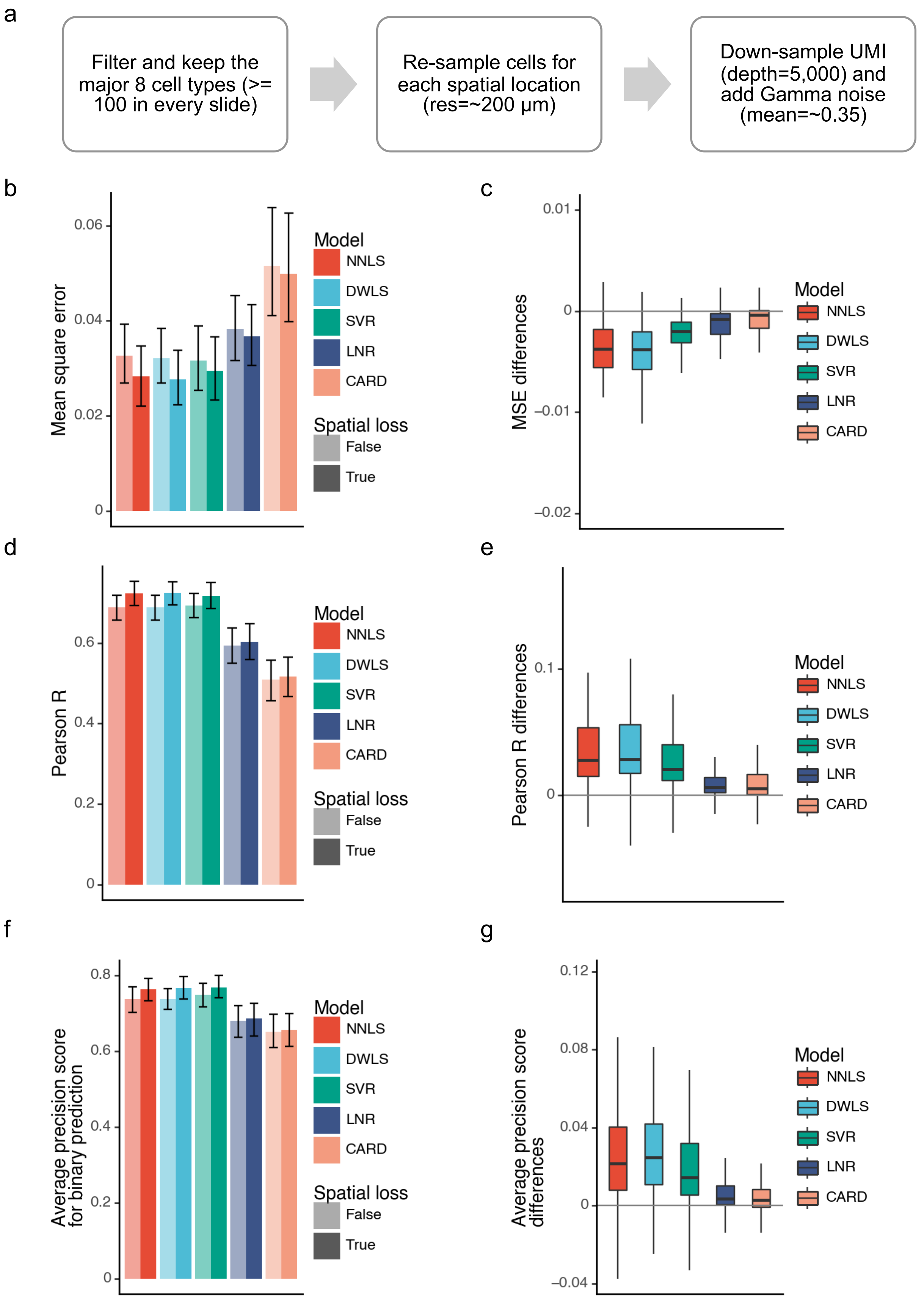

Fig. S14: Evaluation of deconvolution performance on the Sci-Space data of mouse embryo, related to Fig. 2e-h. (a) Schematic overview of the data simulation pipeline. (b-g) Deconvolution performance as measured by mean square error (b, c), Pearson correlation (d, e), and average precision score for binary prediction (f, g). Error bars denote standard error of the mean over all 8 cell types across 14 slides (112 data points in total). Box plots show median, 25th/75th percentiles of the 112 samples, and whiskers indicating 1.5 times the interquartile ranges.

Fig. S15

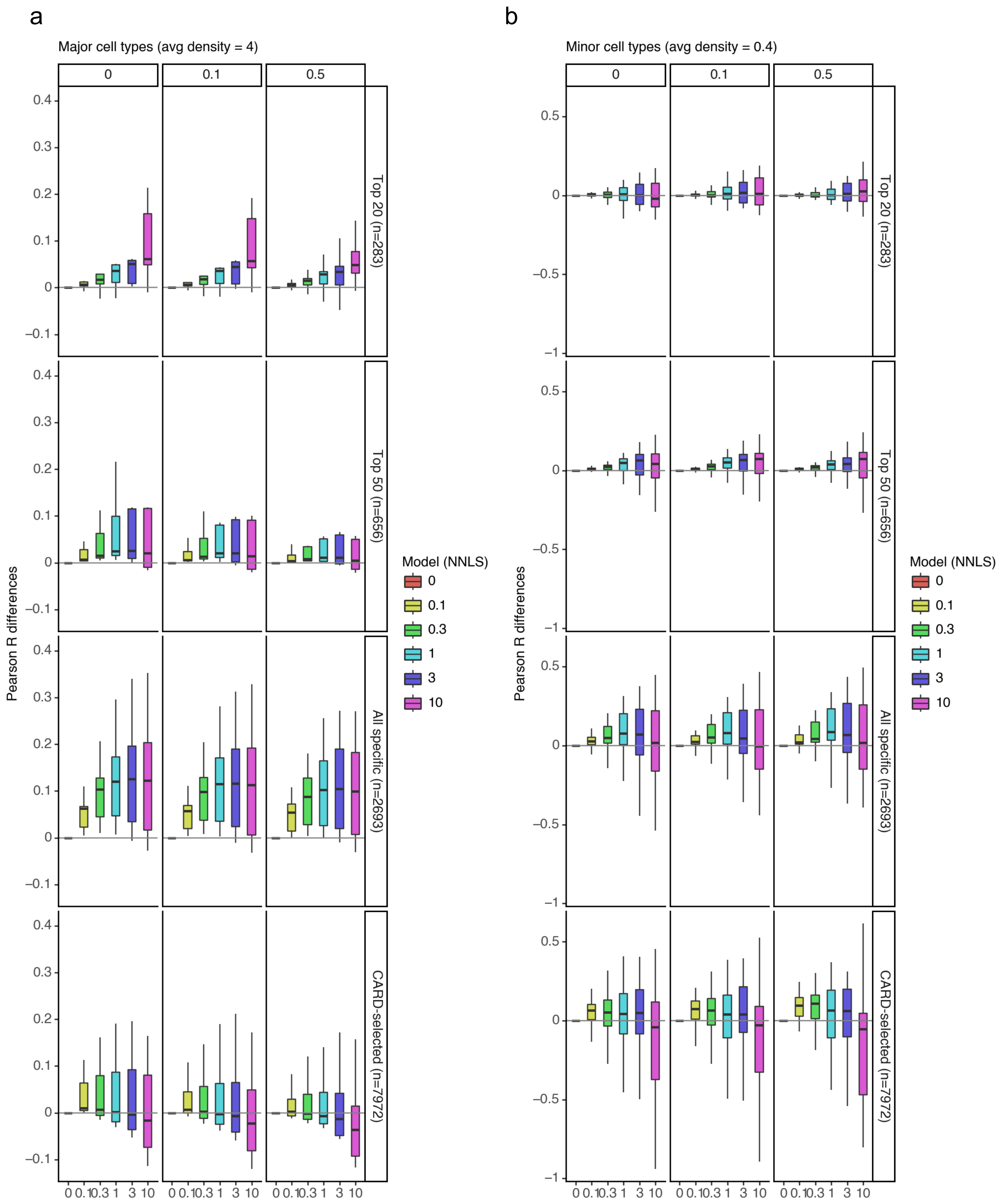

Fig. S15: Robust beneficial effect of spatial regularization in NNLS-based deconvolution.
Box plots display the distribution of deconvolution performance differences after incorporating spatial losses with varying strengths (λ_sp, x-axis). Performance was measured by Pearson correlation, and the coefficient of a non-spatial baseline NNLS model was subtracted from the value of the spatially aware NNLS with different λ_sp for each cell type in each experiment. Each box plot demonstrates the median and the 25th/75th percentiles, and whiskers indicating 1.5 times interquartile ranges indicated by the whiskers of 50 samples for major cell types (a) and 100 for minor cell types (b).

Fig. S16

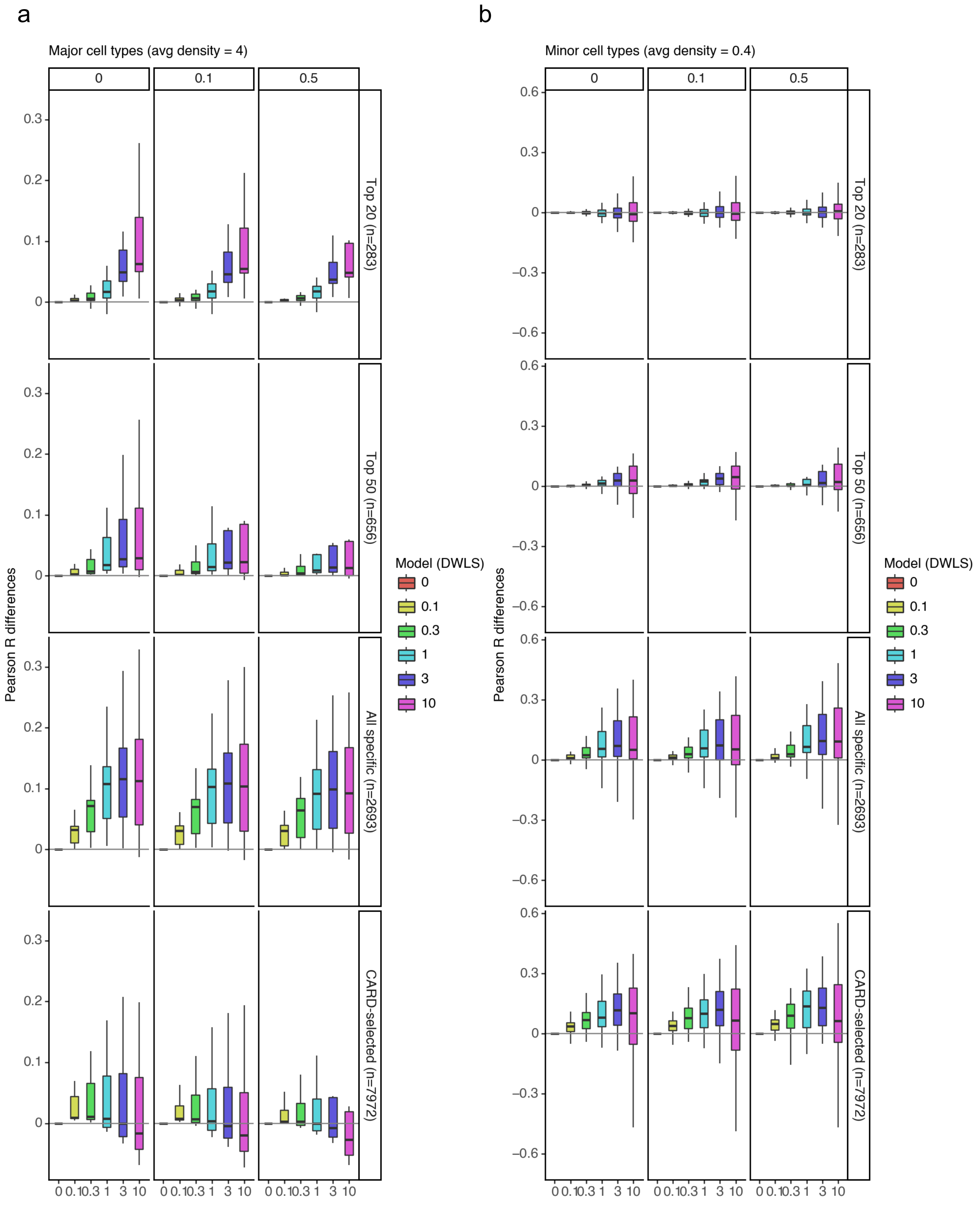

Fig. S16: Robust beneficial effect of spatial regularization in DWLS-based deconvolution.
Similar to **Fig. S15** except the base non-spatial deconvolution model is DWLS.

Fig. S17

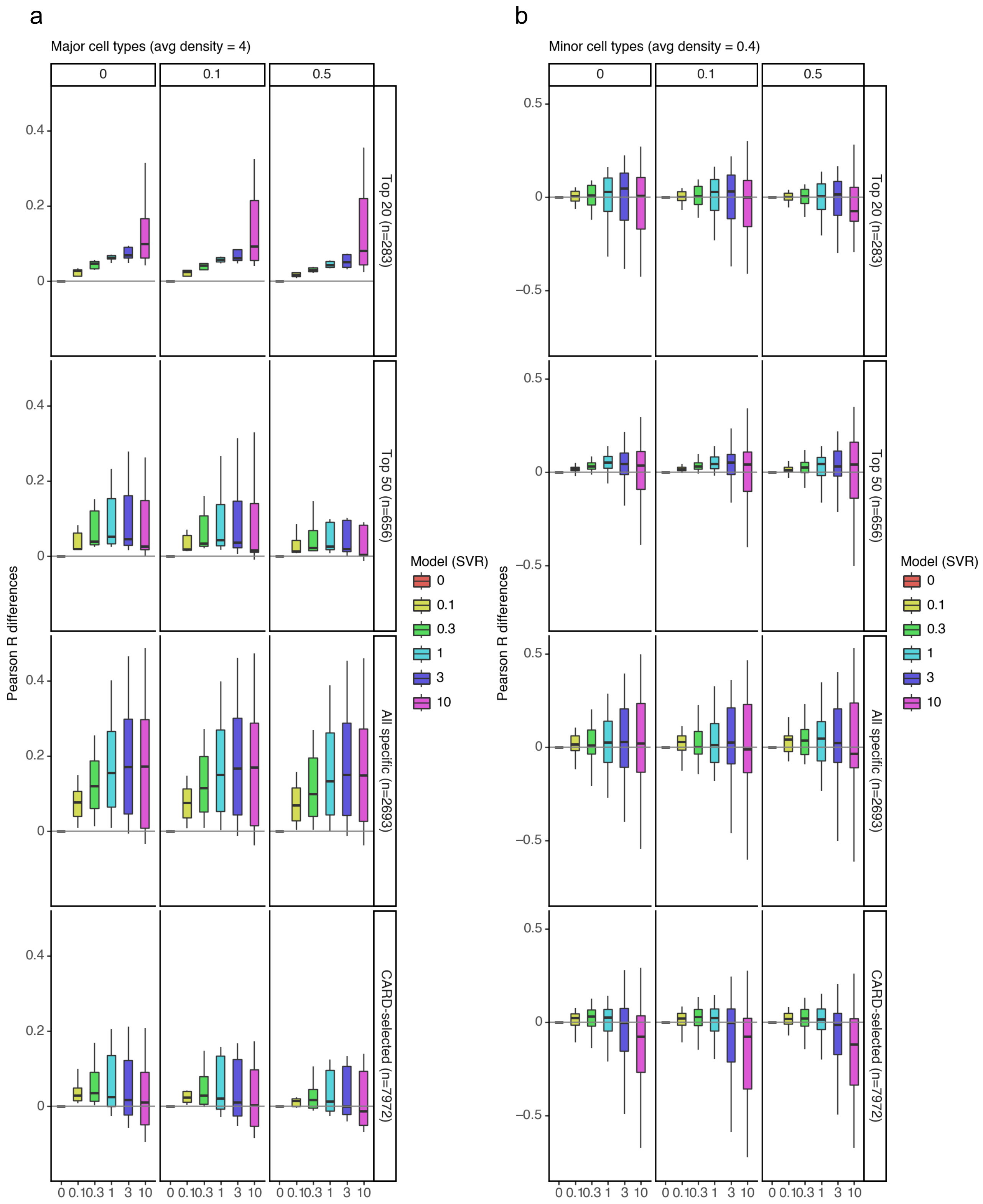

Fig. S17: Robust beneficial effect of spatial regularization in SVR-based deconvolution.
Similar to **Fig. S15** except the base non-spatial deconvolution model is nu-SVR.

Fig. S18

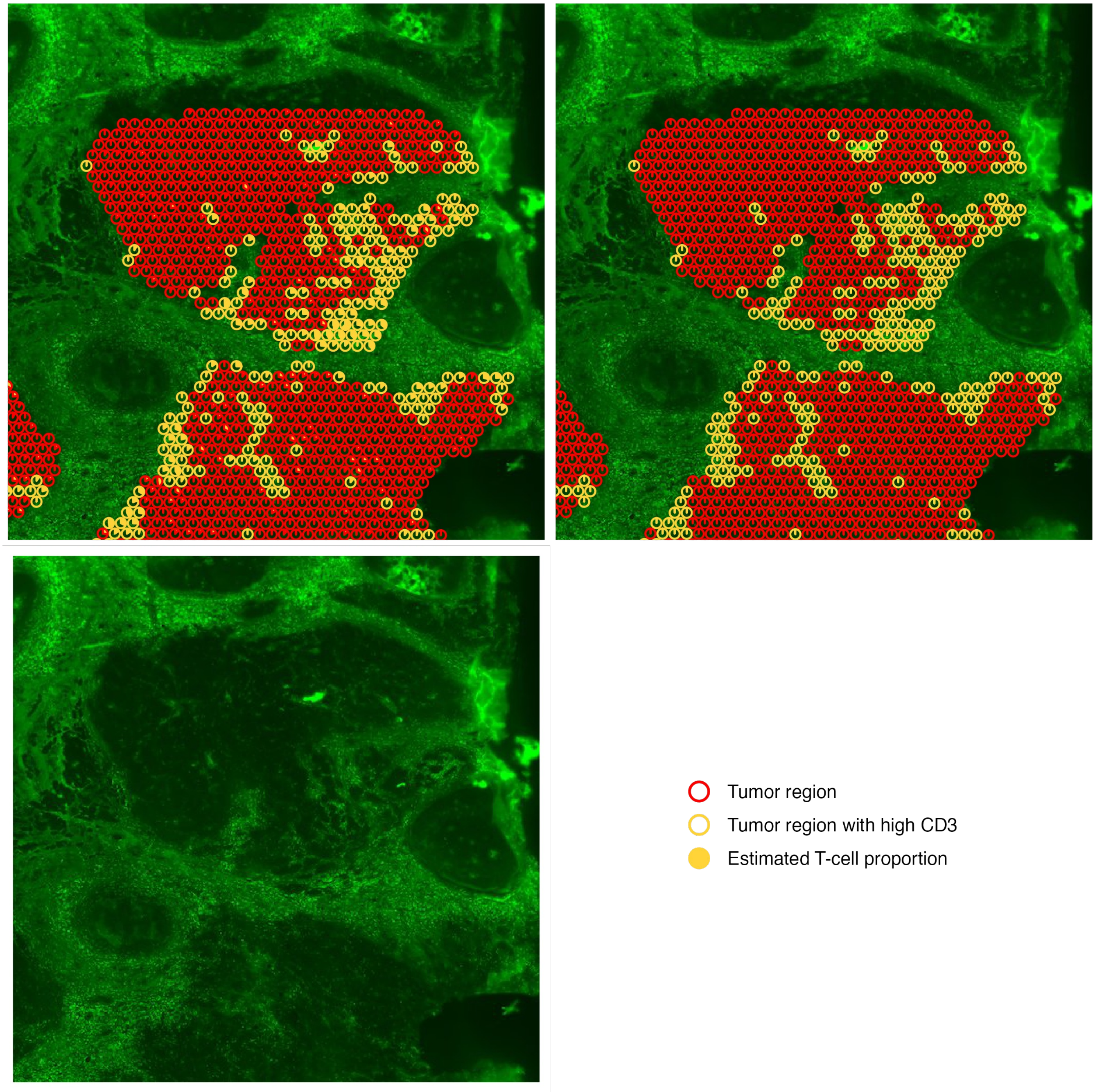

Fig. S18: T-cell infiltration in the zoom-in tumor region of the ductal carcinoma section, related to Fig. 3a-c.
Each circle denotes a tumor occupied spot based on pathological annotation. Spots were classified as CD3+ (yellow) or CD3- (red) according to a two-component Gaussian mixture model on CD3 intensity. Pie charts indicate the estimated T-cell proportions at each spot. Background shows the single channel CD3 immunofluorescence staining (FITC/green) intensity.

Fig. S19

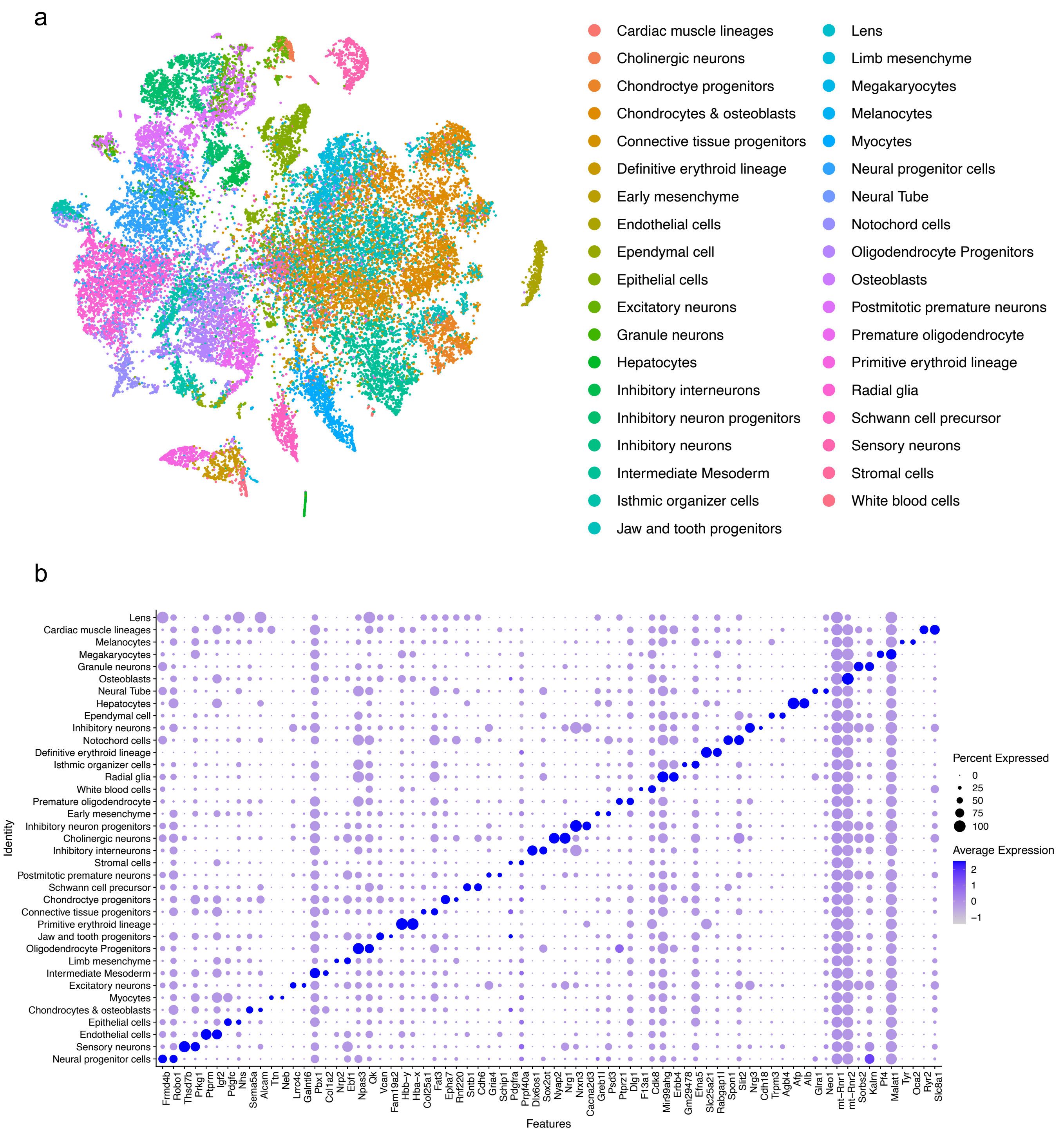

Fig. S19: T-SNE visualization of and marker expression of the scRNA-seq reference from the Mouse Organogenesis Cell Atlas (MOCA), related to Fig 3h-k.

Fig. S20

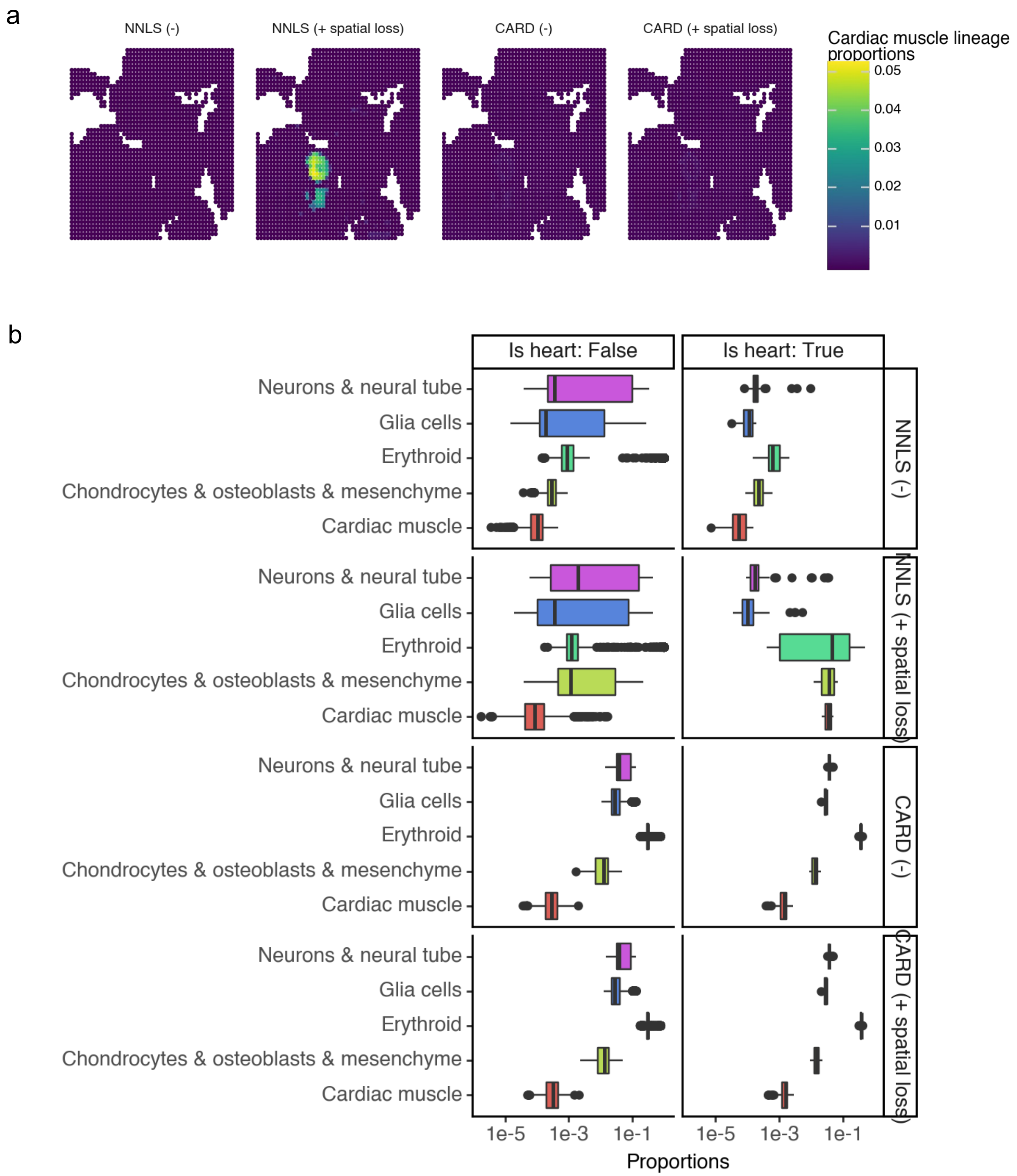

Fig. S20: Detailed examination of deconvolution of cardiac muscle lineage in the spatial-CUT&Tag dataset, related to Fig. 3h-k.

(a) Estimated proportions of cardiac muscle lineage. (b) Distributions of estimated proportions of the five major lineages in and outside the heart region. The heart region is defined by NNLS (+ spatial loss)-estimated proportion >= 0.02. List of cell types in each lineage: 'Neurons & neural tube': cholinergic neurons, excitatory neurons, granule neurons, neural progenitor cells, neural tube, notochord cells, sensory neurons, inhibitory interneurons, inhibitory neuron progenitors, postmitotic premature neurons, inhibitory neurons. 'Glia cells': oligodendrocyte progenitors, premature oligodendrocyte, ependymal cell, Schwann cell precursor, radial glia. 'Erythroid': primitive erythroid lineage, definitive erythroid lineage, megakaryocytes, white blood cells. 'Chondrocytes & osteoblasts & mesenchyme': chondrocyte progenitors, chondrocytes & osteoblasts, osteoblasts, connective tissue progenitors, early mesenchyme, limb mesenchyme'. 'Cardiac muscle': cardiac muscle lineages, myocytes.

Fig. S21

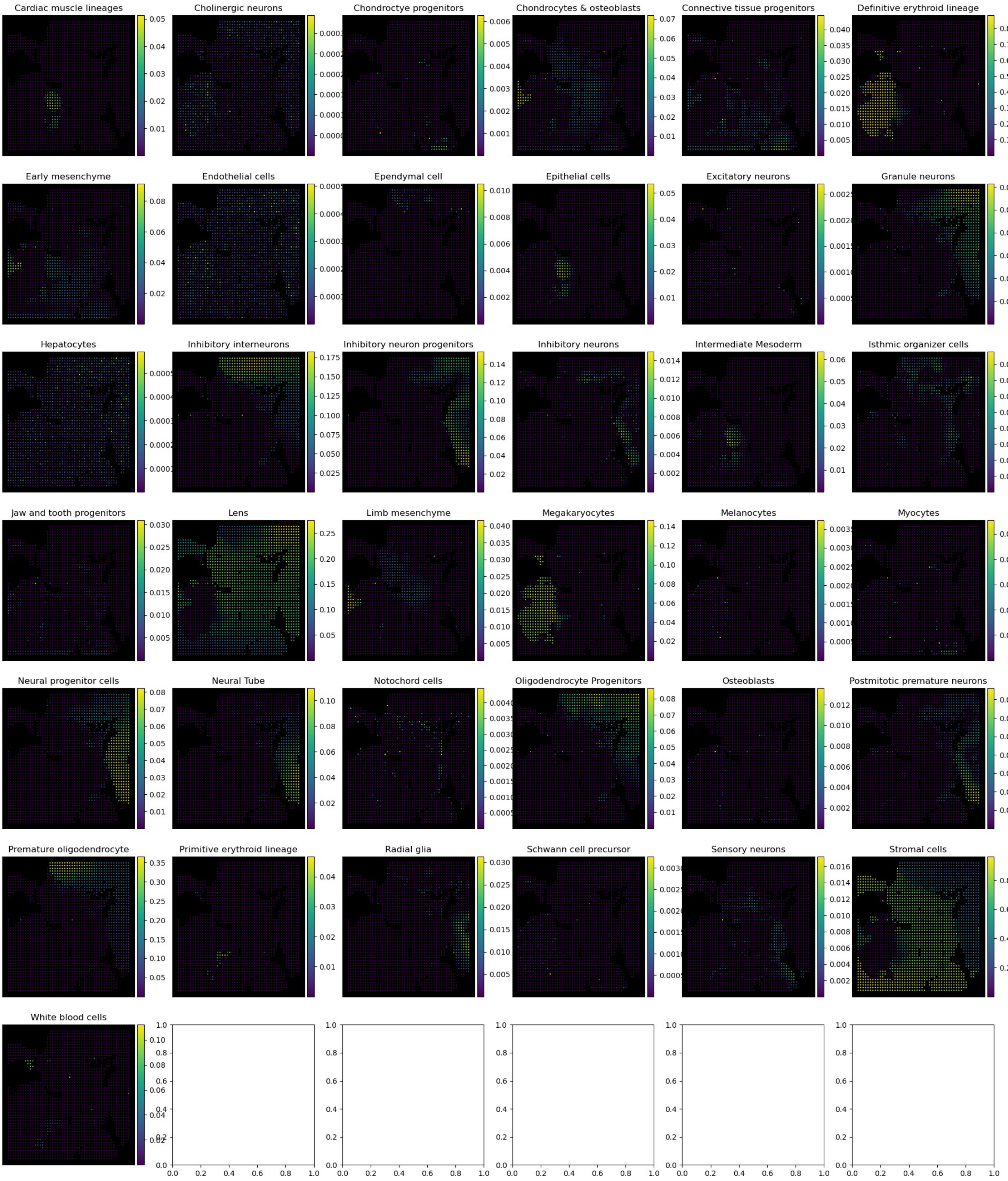

Fig. S21: Per-cell-type deconvolution results of NNLS (+ spatial loss) in the spatial-CUT&Tag dataset, related to Fig. 3h-k.

Fig. S22

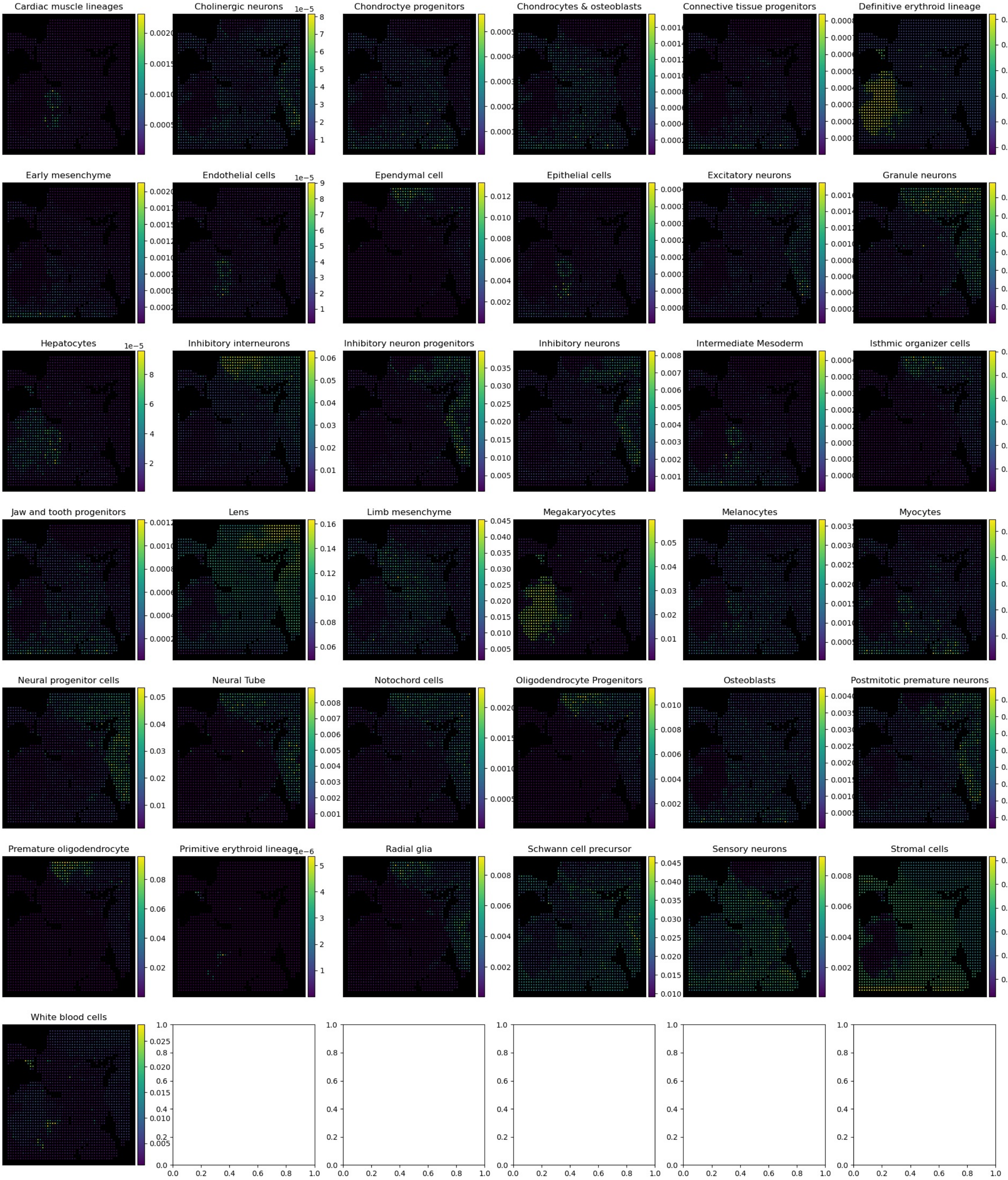

Fig. S22: Per-cell-type deconvolution results of CARD (phi=0.99) in the spatial-CUT&Tag dataset, related to Fig. 3h-k.

Fig. S23

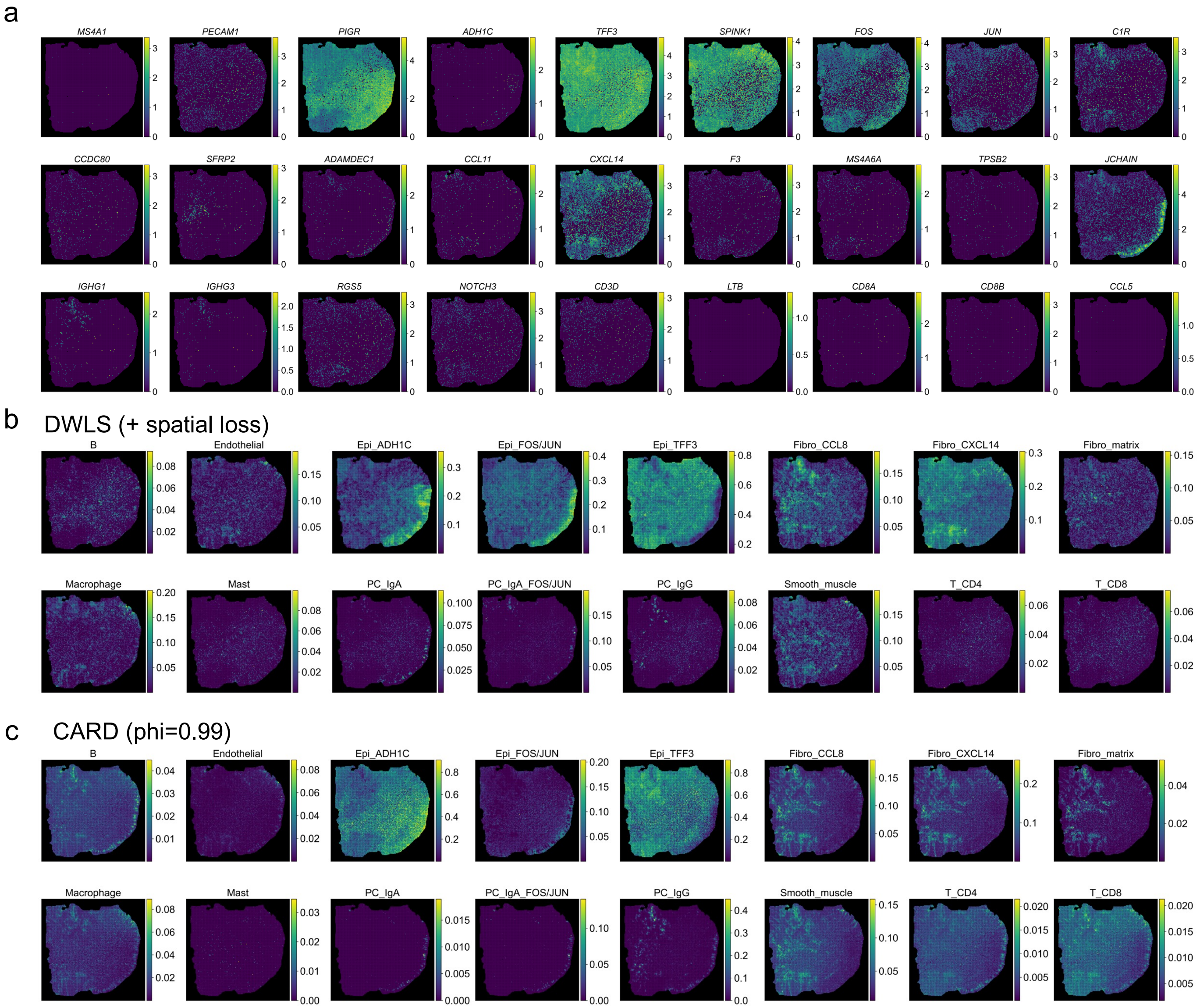

Fig. S23: Full deconvolution results and marker gene expression in the CRC tumor tissue Stereo-seq section, related to Fig. 4. (a) Spatial expression of cell-type specific marker genes. (b-c) Cell type proportions as predicted by Smoother-guided DWLS (b) and CARD with spatial regularization (c).

Fig. S24

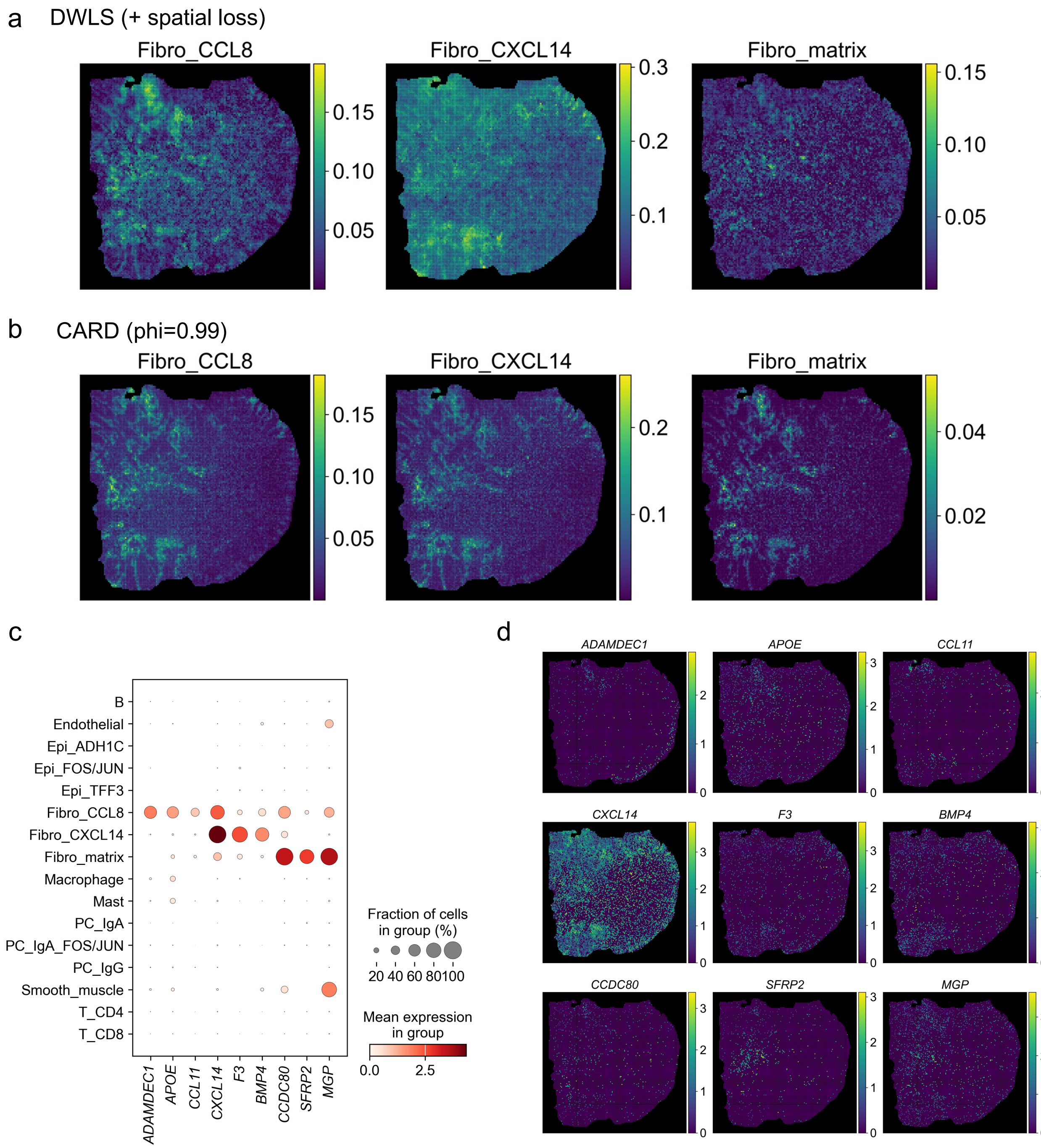

Fig. S24: Comparison between fibroblast subtype deconvolution results and marker gene expression in the CRC tumor tissue Stereo-seq section, related to Fig. 4.

(a-b) Fibroblast subtype proportions as predicted by Smoother-guided DWLS (a) and CARD with spatial regularization (b). (c) Expression dotplot of three marker genes for each fibroblast subtype in the reference scRNA-seq samples, which correspond to previously derived ADAMDEC1+/CCL8+, CXCL14+ and matrix transcriptional programs (Pelka et al.(12)). (d) Spatial expression of the subtype-specific markers shown in (c).

Fig. S25

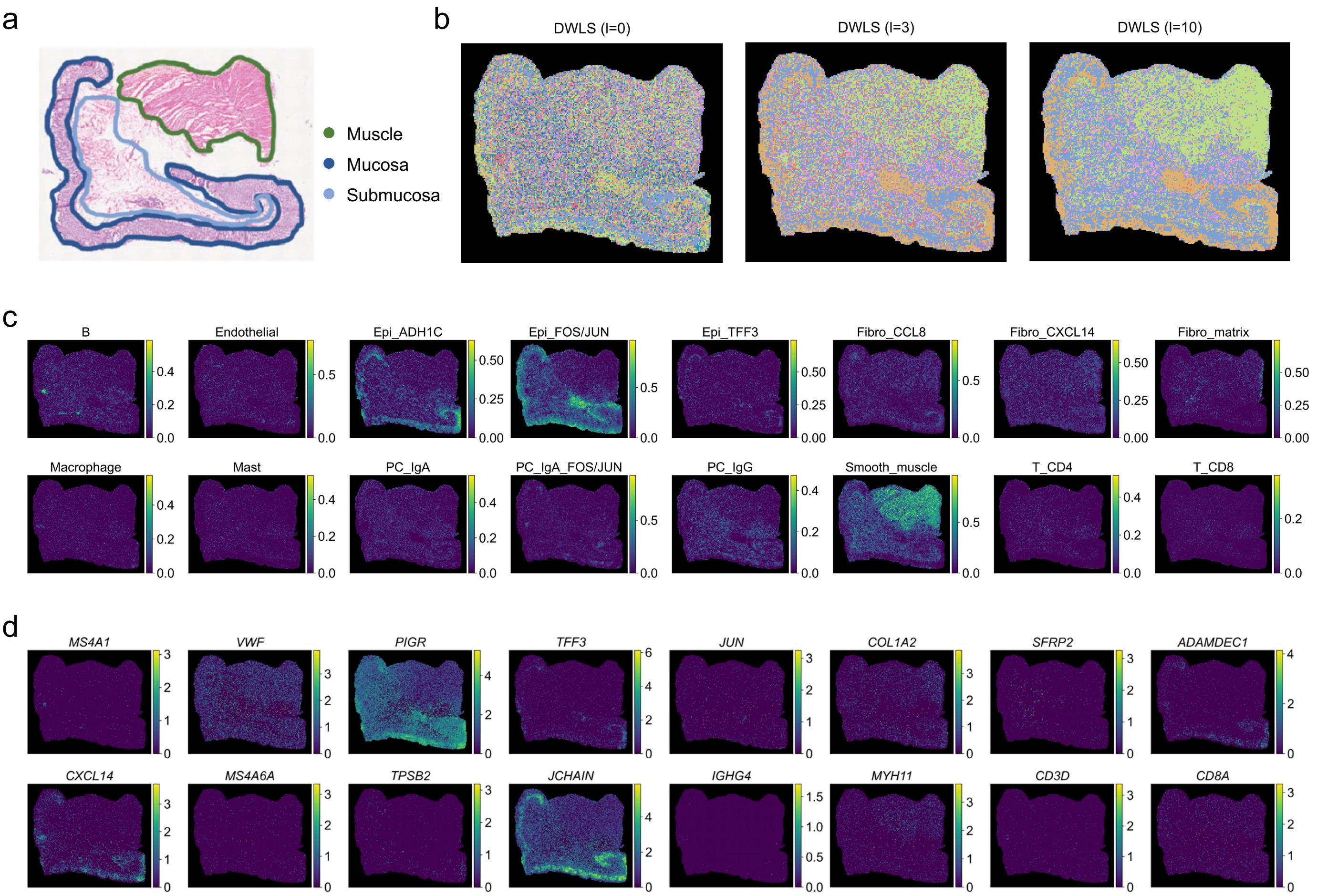

Fig. S25: Deconvolution results and marker gene expression in the adjacent healthy tissue Stereo-seq section of the same CRC patient, related to Fig. 5.

(a) Pathology annotation of the histology slide, adapted from(8). (b-d) Deconvolution performed using the same reference expression matrix as the tumor section. (b) Slide subregions identified by clustering based on Smoother-estimated cell-type compositions, with increasing spatial loss from left to right. (c) Cell type proportions as predicted by Smoother-guided DWLS with spatial loss (l=10). (d) Expression of cell-type specific marker genes.

Fig. S26

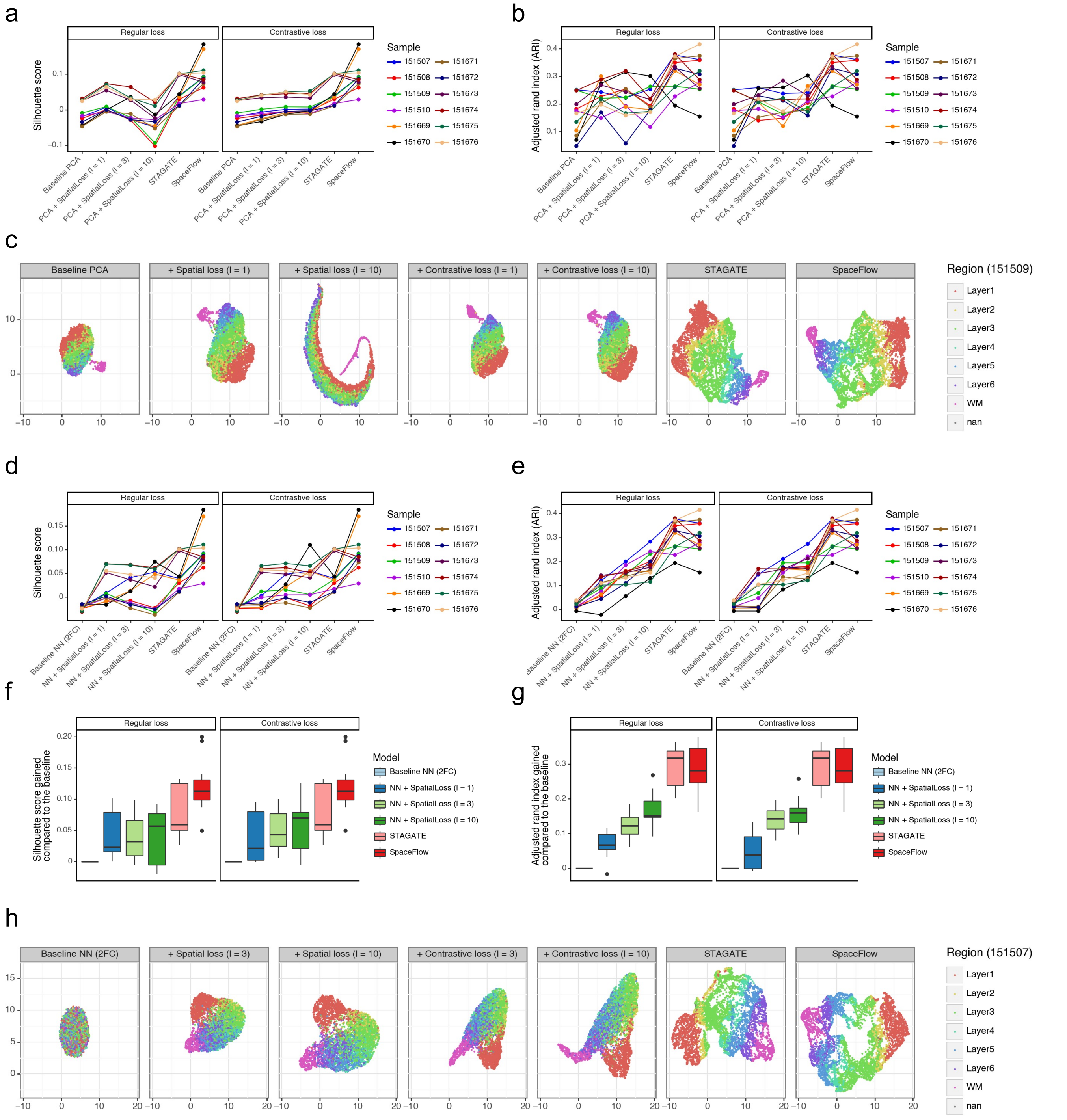

Fig. S26: Comparative analyses of dimensionality reduction performance on the DLPFC dataset(3).
For each sample, we projected either log-normalized expression (PCA) or the raw counts (other models) of the top 2000 highly variable genes onto a 30-dimension latent space using each model to calculate the Silhouette score. ‘Mclust’ was used to cluster spots based on the latent embeddings into the same number of clusters (cortex layers) as observed in the sample. Adjusted rand index (ARI) was calculated based on the clustering results. (a-c) Performance comparisons of PCA models with varying strength of the regular spatial loss and the contrastive spatial loss and the two graph neural networks, STAGATE and SpaceFlow. (c) UMAP visualization of the latent representation learned by each model, colored by spot layer membership. (d-h) Performance comparisons of the baseline neural network with varying strength of the regular spatial loss and the contrastive spatial loss and the two graph neural networks, STAGATE and SpaceFlow. The baseline model was constructed by replacing the two graph attention layers in STAGATE with two fully connected layers of the same number of hidden units (128). (f-g) Performance gains were computed against the baseline for each slide. Each boxplot shows the median and the 25th/75th percentiles over the 12 samples and whiskers indicating the 1.5 times interquartile ranges. (h) UMAP visualization of the latent representation learned by each model, colored by spot layer membership.

Fig. S27

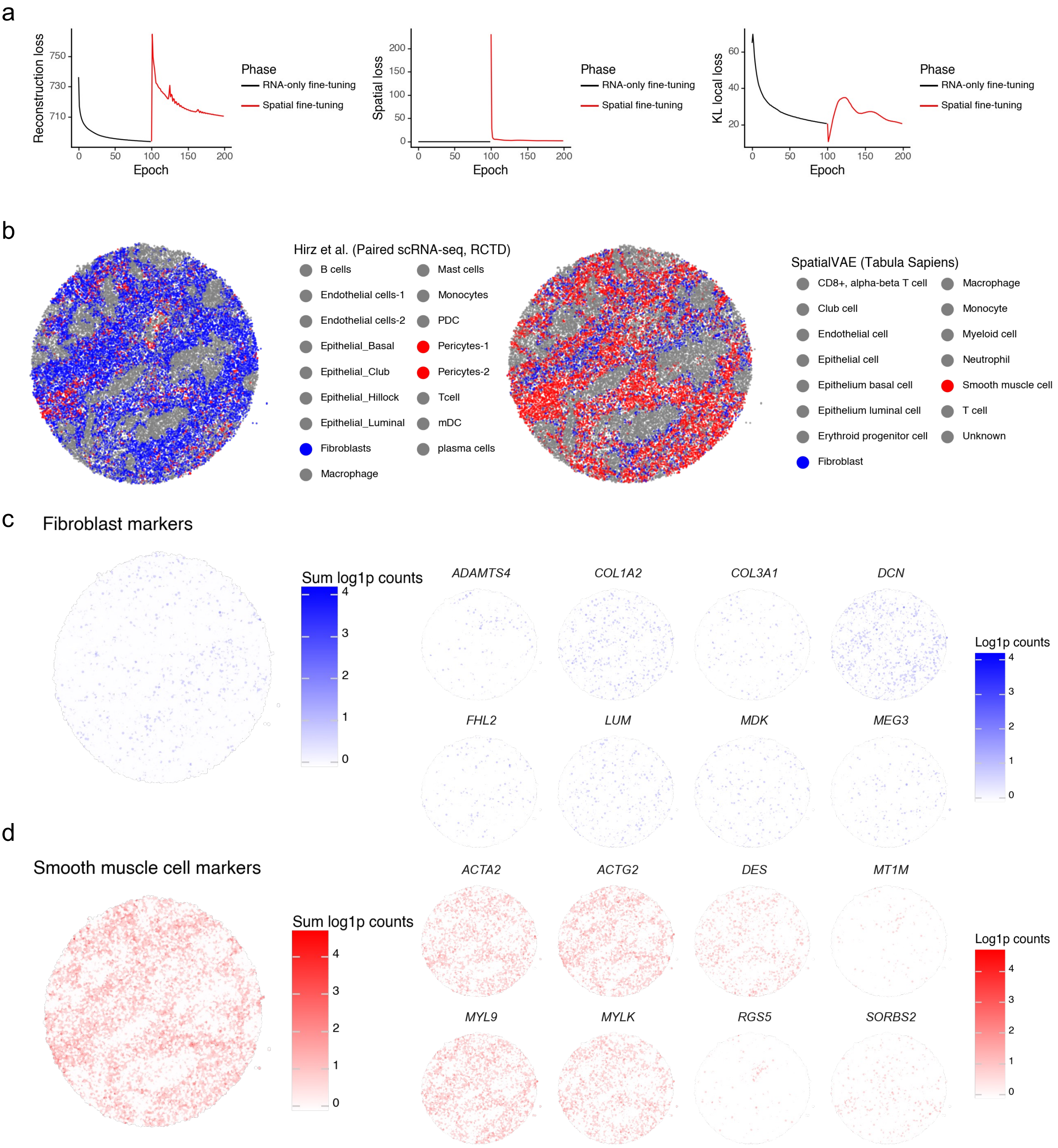

Fig. S27: Spatially aware joint embedding of single-cell and spatial transcriptomics data of human prostate, related to Fig. 5. (a) Visualizations of the training loss of the prostate SpatialVAE model. The overall loss objective is the sum of reconstruction loss (left), spatial loss (middle, zero for RNA-only models), and the KL local loss. Starting from the reference model, we first fine-tuned the model on the Slide-seqV2 data without the spatial loss until convergence (black curve, 100 epoch), then attached the spatial loss and fine-tuned with respect to the new objective (red curve). This is mainly to highlight the tradeoff between reconstruction accuracy and spatial consistency. Skipping the RNA-only fine-tuning step will not affect the performance of the final spatial model. (b-d) Mislabeling of the stromal populations in the original publication (Hirz et al.). (b) Hirz et al. and the Tabula Sapiens use slightly different cell type nomenclatures, where the ACTA2+ population is referred to as pericytes in Hirz et al. and as smooth muscle cells in the Tabula Sapiens. (c) Expression of fibroblast (DCN+, ACTA2- in Hirz et al.) marker genes. (d) Expression of smooth muscle cell (pericytes in Hirz et al., DCN-, ACTA2+) marker genes.

Table S1

Table S1: Computational time of Smoother components when applied to different datasets

Data sets		DLPFC 10X Visium	CRC Stereo-seq	Prostate Slide-seqV2
Number of samples/spots		3,936	18,125	29,278
Computational Time	Spatial loss construction[1]	0.15s	0.8s	1.3s
	Spatial loss per variable per execution[2]	<0.01s	<0.01s	<0.01s
	Data imputation (single feature)	<0.1s	<1s	<1s
	Deconvolution[3]	<1s	<10s	<20s
	Dimensionality reduction[4] (SpatialVAE)	2min	11min	16min

Results computed using 16G RAM M1 MacBook Air, CPU only.

1. The spatial weight matrix is soft-scaled by the expression of top 2000 highly variable genes.
2. Computational complexity = O(n^2), where n is the number of spots.
3. Exact performance depends on the choice of deconvolution algorithms and the number of marker genes.
4. Running 400 epochs with the top 2000 highly variable genes and the following params: n_hidden: 128, n_latent: 10, n_layers: 1, dropout_rate: 0.0, dispersion: gene, gene_likelihood: nb.

References:

1. Wolf FA, Angerer P, Theis FJ. SCANPY: large-scale single-cell gene expression data analysis. Genome Biol. 2018;19.
2. Yuan ZY, Pan WT, Zhao X, Zhao FY, Xu ZM, Li X, et al. SODB facilitates comprehensive exploration of spatial omics data. Nature Methods. 2023;20(3):387-+.
3. Maynard KR, Collado-Torres L, Weber LM, Uytingco C, Barry BK, Williams SR, et al. Transcriptome-scale spatial gene expression in the human dorsolateral prefrontal cortex. Nature Neuroscience. 2021;24(3):425-36.
4. Barkley D, Moncada R, Pour M, Liberman DA, Dryg I, Werba G, et al. Cancer cell states recur across tumor types and form specific interactions with the tumor microenvironment. Nat Genet. 2022;54(8):1192-+.
5. Stickels RR, Murray E, Kumar P, Li JL, Marshall JL, Di Bella DJ, et al. Highly sensitive spatial transcriptomics at near-cellular resolution with Slide-seqV2. Nat Biotechnol. 2021;39(3):313-9.
6. Biermann J, Melms JC, Amin AD, Wang YP, Caprio LA, Karz A, et al. Dissecting the treatment-naive ecosystem of human melanoma brain metastasis. Cell. 2022;185(14):2591-+.
7. Wei XY, Fu SL, Li HB, Liu Y, Wang S, Feng WM, et al. Single-cell Stereo-seq reveals induced progenitor cells involved in axolotl brain regeneration. Science. 2022;377(6610):1062-+.
8. Zhang R, Feng Y, Ma W, Guo Y, Luo M, Li Y, et al. Spatial transcriptome unveils a discontinuous inflammatory pattern in proficient mismatch repair colorectal adenocarcinoma. Fundamental Research. 2022.
9. Deng YX, Bartosovic M, Kukanja P, Zhang D, Liu Y, Su G, et al. Spatial-CUT&Tag: Spatially resolved chromatin modification profiling at the cellular level. Science. 2022;375(6581):681-+.
10. Wu SZ, Al-Eryani G, Roden DL, Junankar S, Harvey K, Andersson A, et al. A single-cell and spatially resolved atlas of human breast cancers. Nat Genet. 2021;53(9):1334-47.
11. Kleshchevnikov V, Shmatko A, Dann E, Aivazidis A, King HW, Li T, et al. Cell2location maps fine-grained cell types in spatial transcriptomics. Nat Biotechnol. 2022;40(5):661-+.
12. Pelka K, Hofree M, Chen JH, Sarkizova S, Pirl JD, Jorgji V, et al. Spatially organized multicellular immune hubs in human colorectal cancer. Cell. 2021;184(18):4734-+.
