# Supplementary material for: Smoother: a unified and modular framework for incorporating structural dependency in spatial omics data
Source: Genome Biol. 2023 Dec 18;24:291. doi: 10.1186/s13059-023-03138-x (PMC10726548; doi:10.1186/s13059-023-03138-x)
Supplement: Supplementary file 2 — Additional file 2. Supplementary Notes on Smoother: A Unified and Modular Framework for Incorporating Structural Dependency in Spatial Omics Data. [file 13059_2023_3138_MOESM2_ESM.pdf]

Supplementary Notes on Smoother: A Unified and Modular Framework for Incorporating Structural Dependency in Spatial Omics Data

Jiayu Su

August 7, 2023

1 Introduction

From subcellular arrangement to tissue compartmentalization, the spatial structure in an organism is highly organized at all scales. This harmonious architecture regulates a diverse variety of biological processes, including embryonic development, neuronal plasticity, and the tumor microenvironment. Recent advances in spatially resolved omics technologies provide unique opportunities to study the spatial patterns of gene and epigenetic activities and the dynamics of biological systems at cellular and tissue levels. However, many sequencing-based techniques have limited spatial resolution, as of 10x Visium[1], and relatively low signal-to-noise ratio, as of spatial-CUT&Tag[2] and spatial-ATAC-seq[3]. To overcome these challenges, it is necessary to take advantage of spatial dependencies underlying the data and develop efficient framework to incorporate the spatial context.

Despite having a common nature and even the same assumption, existing methods for different downstream tasks rely heavily on distinctive and indispensable components to model spatial dependencies, if not ignore them at all. For instance, many specialized deconvolution methods have been developed to recover cell-type composition in space from a mixture of cells captured at the same location, yet few of them[4, 5] consider spatial dependencies, in which the covariance is hard-coded, inalienable and thereby not applicable to other deconvolution models. To unlock the potential of context-guided inference in broader applications, we hereby present Smoother, a unified, modularized and scalable framework to represent structural dependencies in spatial omics data. Smoother explicitly incorporates spatial context through smoothing priors and losses, which we draw from a rich literature of image processing and spatial geographic data analysis. The core idea of Smoother is to borrow information from neighbors and thus to smooth out technical variability.

The rest of the notes is organized as follows: Section 2 lists the notations used in the study and basic mathematical background of the approach. Sections 3-5 detail the incorporation of spatial structure in deconvolution, dimensionality reduction, and data smoothing and imputation, respectively.

2 Background

2.1 Notations

g: gene[1] index
c: cell-type index

[1] For modality other than transcriptomics, g represents the corresponding feature. The same hereinafter.

s: spatial location index
f: spatial feature index
G: number of genes
C: number of cell types
S: number of spatial spots
F: number of spatial features
$M_{i:}, M_{:j}$: the i-th row and j-th column of the matrix M, respectively
$M_{a\times b}$: a matrix of dimension a-by-b
$Y_{G\times S} := \{y_{gs}, g \le G, s \le S\}$: spatial expression profile (of gene g in spatial spot s)
$R_{G\times C} := \{r_{gc}, g \le G, c \le C\}$: reference expression profile (of gene g in cell type c)
$X_{C\times S} := \{x_{cs}, c \le C, s \le S\}$: cell-type abundance (of type c at spatial location s)
$L_{F\times S} := \{l_{fs}, f \le F, s \le S\}$: spatial metadata where $L_{.s}$ is the concatenated spatial feature vector (coordinates, histological images, etc.) at spot s

2.2 Spatial stochastic process

This section introduces the mathematical concept of spatial stochastic process. Since we only measure the profile of each spatial spots once in spatial omics experiments, we must assume certain degrees of stationarity, such that it becomes possible to infer the correct spatial structure from the single observation. A spatial stochastic process is a stochastic process that specifies the spatial dynamics of some random variables, often by stating their joint distributions. We will derive the spatial covariance structure from local connectivity specified in the stochastic process and use it as a prior to guide downstream tasks.

2.2.1 Spatial moving average process

Spatial moving average (SMA) models assume that each random variable is a weighted sum of a random innovation at the location (an innovation part) and an average of random innovations at neighboring locations (a smoothing part):

$$s_i = u_i + \gamma \sum_{j\neq i} w_{ij} u_j$$

where s_i is the spatial variable of interest at location i, u_i is the innovation at location i, w_{ij} is part of a weight matrix specifying the connectivity between location i and j, and γ is the smoothing parameter. The equation above can also be expressed in matrix form

$$s = u + \gamma W u = (I + \gamma W)u.$$

To see the spatial covariance structure of s, we may further assume that u_i is i.i.d. and zero-centered. Using the above equation we know that s is also zero-centered and

$$Cov[s] = E[ss'] = E[(I+\gamma W)uu'(I+\gamma W)'] = \sigma^2(I + \gamma(W+W') + \gamma^2 WW'). \tag{1}$$

This tells us that under a SMA model, the spatial smoothing effect is local. There can only be at most first-order and second-order dependencies among locations, since s has zero covariance beyond second-order neighbors.

2.2.2 Spatial autoregressive process

On the contrary, autoregressive (AR) models assume that each random variable at a location depends directly on random variables at neighboring locations, thereby creating feedbacks between locations and allowing

distant pairs to be connected by a more global smoothing effect. In this study, we consider the simultaneous autoregressive (SAR) model and the conditional autoregressive (CAR) model. While the two are mostly equivalent after re-parameterization[6], in their natural forms SAR and CAR each specifies a slightly different covariance structure.

Intuitively, SAR is a model for complete spatial patterns, meaning that dependencies can be reduced and values at all locations can be explained as functions of exogenous variables. For example, the simultaneous equations (in matrix form)

$$s = \rho W s + u$$

is equivalent to the reduced form

$$s = (I - \rho W)^{-1} u$$

provided that the matrix inverse exists (which is determined by the smoothing parameter ρ). Again, if we assume that u_i is i.i.d. and zero-centered, we would have

$$Cov[s] = E[ss'] = E[(I - \rho W)^{-1} uu' (I - \rho W')^{-1}] = \sigma^2 [(I - \rho W)'(I - \rho W)]^{-1}. \quad (2)$$

Because of the matrix inverse here, the variance is no longer sparse and every location is expected to co-vary with every other location. In other words, the smoothing effect is rather global. To simplify the search for a proper value of ρ, we can normalize the weight matrix W by the sum of each row. This gives us the intrinsic simultaneous autoregressive (ISAR) model and its covariance:

$$s = (I - \rho \tilde{W})^{-1} u, \tilde{w_{ij}} = \frac{w_{ij}}{\sum_j w_{ij}}$$
$$Cov[s] = \sigma^2 [(I - \rho \tilde{W})'(I - \rho \tilde{W})]^{-1}, \rho \in [0, 1].$$

CAR is a model for prediction, and is commonly used as the spatial prior for parameters in a hierarchical model. It builds on the idea that the joint distribution can be factorized into a product of conditional distributions and marginal distributions. CAR models usually specify the full conditional distributions, and are interested in deriving the joint distribution using those conditionals

$$p(s) = p(s_1, ..., s_n) = \prod_i p(s_i | s_{\setminus i}).$$

The Brook's Lemma establishes link between full conditionals and the joint distribution. However, such factorization is not always achievable: Individual conditionals can be incompatible, and the resulting joint distribution may be improper. Even when a factorization does exist, there is generally no guarantee that the factorization is unique. Here we focus on Markov Random Field (MRF), a subclass of CAR models that acquires some nice properties from the Markov assumption. The Hammersley-Clifford theorem states that for any MRF model that defines a proper (and unique) joint distribution through local conditionals in concordance with its Markov properties, the joint distribution is a Gibbs distribution that can be factorized over cliques (fully connected sub-graphs), and vice versa. Ignoring high-order dependencies, the theorem gives us the following factorization:

$$Q(x) := \frac{P(x)}{P(0)} \propto exp(\sum_i G_i(x_i) x_i + \sum_{i,j} G_{i,j}(x_i, x_j) x_i x_j)$$

where G is an appropriate Gibbs energy function. In particular, if $Q(x)$ can be parameterized as the density of a multivariate Gaussian distribution, then the conditionals and marginals are also normal. This model is called the auto-normal MRF model or the Gaussian Markov Random Field (GMRF) model:

$$s_i | s_{\setminus i} \sim \mathcal{N}(\sum_{j \neq i} w_{ij} s_j, \sigma_i^2)$$

$$p(s_i|s_{\setminus i}) \propto exp(-\frac{1}{2}\sigma^{-2}(s_i - \sum_{j \neq i} w_{ij}s_j)^2)$$

$$Q(s) \propto exp(-\frac{1}{2}s'D^{-1}(I - W)s) \tag{3}$$

where $D = diag(\{\sigma_i^2\})$.

For $Q(s)$ to be proper, the covariance matrix $[D^{-1}(I - W)]^{-1}$ must be symmetric and positive-definite. We may introduce another parameter ρ to restrict $D - \rho W$ to the proper space. An improper joint distribution, however, can still be useful when applied as a prior in a hierarchical Bayesian model, as the conditional setting is directly linked with Gibbs sampling. Such models include the intrinsic conditional autoregressive (ICAR) model, where the spatial weight matrix W^* is normalized by the following transformation

$$s_i|s_{\setminus i} \sim \mathcal{N}(\frac{1}{\sum_j w_{ij}} \sum_j w_{ij}s_j, \frac{\sigma^2}{\sum_j w_{ij}})$$

and the joint density thus becomes

$$Q(s) \propto exp(-\frac{1}{2}s'(D_w - W)s) \tag{4}$$

where $D_w = diag(\{\sum_j w_{ij}\})$. Here $Q(s)$ is both improper ($D_w - W$ is singular) and non-identifiable (adding any constant to all s will give the same conditionals and thus the same joint density).

2.3 Practical guides on selecting the spatial prior

Smoother offers five diverse yet related spatial process models, namely SMA, CAR, SAR, ICAR, and ISAR. As introduced above, the SAR and CAR are mathematically equivalent with appropriate transformation. We included both models to offer users the flexibility to choose their preferred parameterization. This is analogous to choosing between the mean-dispersion and the size-prob parameterizations of the negative distribution. Additionally, we also introduced ICAR and ISAR as the weights-scaled versions of CAR and SAR. This is for convenience so that the spatial autocorrelation hyperparameter ρ falls in [0, 1], which controls the positive definiteness of the resulting covariance.

Empirically, the specific choice of the spatial process does not have a significant impact as long as the final covariance structure is similar. That is, an ICAR model with a smaller ρ will yield the same effect as a more local SMA model. In practice, we recommend using ICAR with varying ρs (or ISAR with smaller ρs) to accommodate data with different dependencies. For example, 'ICAR (ρ=0.99)' for data with clear anatomy and 'ICAR (ρ=0.9)' for tumor data. This is based on pure numeric considerations, as the inverse covariance matrix of the SMA tends to be less sparse, potentially slowing down computation.

2.4 MRF in spatial smoothing and related works

MRF and its extensions have long been deployed to introduce spatial smoothness and consistency in different applications, including image segmentation[7, 8], range sensing[9], and geographical clustering[10]. On the analyses of spatial transcriptomics data in particular, we document the following models that builds upon MRF to share information across locations: Giotto[11, 12] develops a hidden Markov random field (HMRF) model to identify spatial patterns in gene expression and cluster spots into spatial domains. BayesSpace[13] implements a similar HMRF in the Bayesian framework and enhances the spatial resolution by sampling data at each latent unobserved sub-spot. Spicemix[14] and DR-SC[15] connect HMRF clustering with factorization-based dimensionality reduction and jointly optimize the two components. STARCH[16] uses HMRF to detect copy number variations in spatial transcriptomics data of tumor. CARD[4] introduces a Gaussian CAR prior

on cell-type abundances to improve deconvolution accuracy and impute data for unseen locations at greater resolution. SpatialPCA[13] proposes a variant of probabilistic PCA that imposes a similar GMRF prior on latent low-dimensional representations. Despite the fact that all of these methods relies on similar MRF structures, each method implements and solves the joint model individually (often via different approaches, i.e. gradient- and Hessian-based optimizations, EM or MCMC sampling). This makes it hard if not impossible to modify and share the spatial covariance structure of the same data across different applications like deconvolution and dimensionality reduction. Moreover, either extending the spatial component to include additional information, such as histology images or region boundaries, or updating the non-spatial component to fit new data with different properties, such as the spatial-CUT&Tag data[2] and the spatial-ATAC-seq data[3], would require the overwriting of the entire method. To address these issues, we hereby present a unified, modular, versatile, and scalable framework for the design and modeling of structural dependencies in spatial omics data.

3 Deconvolution models with spatial priors and losses

3.1 Problem formalization

We define spatial deconvolution as the problem of inferring cell-type abundances at each location from the observed omics data, with or without cell-type reference information from external data. In a typical in situ capture experiment (like 10x Visium), each spatial spot captures a mixture of individual cells. We denote the spatial omics data as a gene-by-spot count matrix $Y_{G\times S}$ where y_{gs} is the expression level of gene g at spot s, and additional information as the metadata $L_{F\times S}$ where $L_{.s}$ is the concatenated spatial feature vector (coordinates, histological images, etc.) at spot s. Now we have

$$\begin{aligned} Y_{G\times S} &= Generative(R_{G\times C}, X_{C\times S}) \\ X_{C\times S} &= Deconv(Y_{G\times S}, R_{G\times C}) \end{aligned} \tag{5}$$

where x_{cs} is the of interest cell-type abundance of type c at spatial location s, r_{gc} is the external reference expression of gene g in cell type c (if needed), and *Generative* and *Deconv* are the generative and the corresponding deconvolution models, respectively. In the simplest case of a linear generative model, the deconvolution is essentially a least squares regression problem:

$$\begin{aligned} Y_{G\times S} &= R_{G\times C} X_{C\times S} \\ X_{C\times S} &= (R'R)^{-1} R'Y. \end{aligned} \tag{6}$$

The key advancement of our work is to incorporate additional spatial information through the design of spatial prior on $X_{c:}$ of cell type c

$$X_{c:} \sim SpatialPrior(Y, L).$$

In the following sections we will present a series of spatially aware deconvolution models.

3.2 Linear regression with spatial smoothing priors

We start with a simple linear deconvolution model. Here, the observed expression of g at spatial location s is generated by the linear combination of all cells captured at that location

$$y_{gs} = \sum_c r_{gc} x_{cs} + \delta_{g,s} = R_{g.} X_{.s} + \delta_{gs}$$

where r_{gc} is the reference expression of gene g in cell type c, x_{cs} is the abundance for cell type c at spatial location s, and δ_{gs} is the noise term. If we further assumed δ_{gs} to be Gaussian i.i.d., then $X_{C\times S}$ can be solved via ordinary least squares

$$\tilde{X}_{MLE} = \underset{X}{\text{argmax}}\, P(Y|X) = \underset{X}{\text{argmin}}\, \|Y_{G\times S} - R_{G\times C}X_{C\times S}\|_2. \quad (7)$$

By applying spatial priors on either X or δ, we may introduce and model spatial dependencies in the observed data Y. We reason that shared cell-type composition is the main source of spatial dependence in the data. Therefore, for parameter identifiability, we only add spatial prior on $X_{c:}$ and assume δ_{gs} to be i.i.d. This gives us the following model

$$\begin{aligned} y_{gs} &\sim \mathcal{N}(R_{g.}\beta_{.s}, \sigma^2) \\ X_{c:} &\sim MVN(\mu_c, \Sigma_c) \end{aligned}$$

where Σ_c is the spatial covariance matrix imposed on cell type c. Similar to the probabilistic interpretation of L2 regularization in the context of linear regression, solving the above model for X is equivalent to minimizing the mean squared error eq. (7) plus a regularization term. For simplicity, we may assume cell types to be independent and $X_{c:}$ to be zero-centered, so that the deconvolution task can be described as the following optimization problem

$$\tilde{X}_{MAP} = \underset{X}{\text{argmax}}\, P(X|Y) = \underset{X}{\text{argmin}}\, \|Y_{G\times S} - R_{G\times C}X_{C\times S}\|_2^2 + \lambda \sum_c X_{c:}\Sigma_c^{-1}X'_{c:} \quad (8)$$

where λ is an additional parameter to control the relative contribution of spatial covariance to the overall variance in the data. Additional constraints like non-negativity may be imposed on X. Note Σ is defined by the underlying stochastic process eqs. (1) to (4) and is constructed a priori. If Σ and thus the joint distribution of X are properly defined, which depends on the specific choice of W and the prior spatial covariance strength ρ, then the objective function is convex. For more general cases, the above problem can still be solved via gradient descent.

$$\begin{aligned} \Sigma_{SMA} &= \sigma^2(I + \rho(W + W') + \rho^2 WW') \\ \Sigma_{SAR} &= \sigma^2[(I - \rho W)'(I - \rho W)]^{-1} \\ \Sigma_{CAR} &= \sigma^2(I - \rho W)^{-1} \\ \Sigma_{ICAR} &= (D_w - \rho W)^{-1} \end{aligned}$$

To impose different structures, one can either select different models or, more directly and efficiently, modify the spatial weight matrix W. For example, w_{ij} can be a function of the relative physical and transcriptomics distances between spot i and j.

3.3 Relationship to CARD

Conditional autoregressive-based deconvolution (CARD)[4], as its name suggests, is another deconvolution method that imposes conditional autoregressive prior on cell-type abundance to model spatial dependencies in the data. Mathematically, CARD's objective function is similar to eq. (8) except CARD: (a) uses Gaussian kernel to construct the covariance matrix Σ, which is based solely on physical distances, and (b) has additional parameters such as the mean and variance of the abundance distribution of each cell type. In particular,

$$\begin{aligned} \Sigma_{CARD} &= \lambda_c(D_w - \rho W)^{-1} \\ w_{ij} &= \exp\left(-\frac{\|s_i - s_j\|^2}{2\sigma^2}\right) \end{aligned}$$

where s_i is the spatial location of spot i, σ is the user-defined bandwidth, D_w is the diagonal matrix with the sum of each row of W as its diagonal elements, λ is the cell-type-specific variance, and ρ is the strength of

spatial dependency. CARD performs an internal grid search to find the optimal ρ. In benchmark analyses, we turned off the spatial covariance component by manually setting ρ to zero (the covariance will be diagonal but not isotropic).

We argue that our formulation is better than CARD since (a) by modularizing the spatial loss, users have more control over the design of spatial dependence, and the same mechanism can be applied to different models, also (b) by assuming zero-centered abundances, the model is simpler to train and introduces regularization to help increase the robustness of solution. In particular, we parameterize eq. (8) such that the strength of spatial dependence is controlled by both the prior knowledge (Σ) and data- or task-specific contribution (λ). Implementation-wise, we solve the above problem in the PyTorch framework (and also CVXPY for convex objectives). This allows us to customize or generalize the model and objective without the need to implement a new optimization algorithm each time. The spatial loss module can also be easily incorporated into other machine learning and deep learning models. Taken together, our formulation has advantages in both deconvolution and other applications like data imputation and dimensionality reduction.

3.4 Spatially aware deconvolution models implemented in this study

Leveraging the rich literature on gene expression deconvolution, we seek to demonstrate the versatility of our model by equipping existing models with the newly proposed spatial loss. In this study, we implement and solve the following models using PyTorch, and have conducted extensive benchmarking analyses in both synthetic and real datasets.

3.4.1 NNLS

This is the simple extension of eq. (8) where X is constraint to be non-negative. By default, we also introduce a bias term γ to account for additional spot-specific effects.

$$\tilde{X} = \underset{X \geq 0}{\operatorname{argmin}} \frac{1}{GS} \|Y_{G\times S} - R_{G\times C}X_{C\times S} - \gamma_{1\times S}\|_2^2 + \frac{\lambda}{CS} \sum_c X_{c:}\Sigma_c^{-1}X'_{c:} \tag{9}$$

3.4.2 DWLS

This model is the extension of the dampened weighted least squares (DWLS) method[17] that mitigates biases against rare cell types by adjusting the contribution of each gene. One key difference is that we use observed expression ($Y_{G\times S}$) instead of the predicted expression ($R_{G\times C}X_{C\times S}$) to calculate the dampened weights. Empirically the modification speeds up the convergence and leads to more stable solutions.

$$\begin{aligned} \tilde{X} &= \underset{X \geq 0}{\operatorname{argmin}} \frac{1}{GS} \|W_{G\times S} \odot (Y_{G\times S} - R_{G\times C}X_{C\times S} - \gamma_{1\times S})\|_2^2 + \frac{\lambda}{CS} \sum_c X_{c:}\Sigma_c^{-1}X'_{c:} \\ W_{gs} &= \min(\frac{\max_g(y_{gs}^2)}{y_{gs}^2}, w_0) \in [1, w_0] \end{aligned} \tag{10}$$

where w_0 is a user-defined parameter that sets the upper limit of weights.

3.4.3 ν-SVR

Support vector regression (SVR) is a generalization of support vector machine (SVM) where a hyperplane is fitted to data points within the given distance. One of the widely used bulk expression deconvolution methods, CIBERSORT[18], uses linear ν-SVR to adaptively select informative genes such that the deconvolution

performance is more robust against batch effects between the reference and the spatial omics data. Here we parameterize the model as follows:

$$\begin{aligned}\tilde{X}, \tilde{\epsilon} &= \underset{X\geq 0, \epsilon \geq 0}{\operatorname{argmin}}\ C(\nu\epsilon + \frac{1}{GS}L_\epsilon) + \frac{1}{cs}\|X\|_2^2 + \frac{\lambda}{CS}\sum_c X_{c:}\Sigma_c^{-1}X'_{c:} \\ L_\epsilon &= \|\max(|Y_{G\times S} - R_{G\times C}X_{C\times S} - \gamma_{1\times S}| - \epsilon, 0)\|\end{aligned} \tag{11}$$

where C and ν are user-defined penalty parameters that control the strength of regularization and the allowance of outliers, respectively.

3.4.4 Log-normal regression (LNR)

This model is described in SpatialDecon[19] where the least squares regression on the raw counts is replaced by log-normal regression to better model variability of skewed data. Here we only consider the log-normal deconvolution module of SpatialDecon. Accordingly, the objective function is the following:

$$\tilde{X} = \underset{X\geq 0}{\operatorname{argmin}}\ \frac{1}{GS}\|\log(Y_{G\times S} + \epsilon) - \log(R_{G\times C}X_{C\times S} + \gamma_{1\times S} + \epsilon)\|_2^2 + \frac{\lambda}{CS}\sum_c X_{c:}\Sigma_c^{-1}X'_{c:} \tag{12}$$

where ϵ is the pseudo-counts (by default 1).

3.4.5 Notes on selecting the scale for deconvolution

While we make no additional assumption on the characteristics of spatial omics data except the Gaussian assumption implied in L2-norm-related losses, deconvolution performance certainly depends on the distribution properties of input data. For example, RNA-seq counts data are typically skewed and, if not corrected, can bias the estimation against lowly expressed genes and rare cell types. One natural and simple solution is to perform deconvolution on the logarithmic scale, i.e., replacing Y and X with $\log(Y+1)$ and $\log(X+1)$ in eqs. (9) to (11). This approach is not physically sound, but empirically the log-scale has been shown to improve model performance by large margins, as do the square root scaling $\sqrt{Y}$ and $\sqrt{X}$ albeit to a less extent (data not shown). In contrast, the physically plausible model eq. (12) is not as robust as expected. One possible explanation is that the objective is non-convex and the optimization of β is not as efficient as other least squares models.

Unless otherwise stated, we supplied log-scaled transcriptomics data to NNLS, DWLS, and ν-SVR and raw scale data to LNR and CARD in our benchmark analysis. For general deconvolution cases, such as in spatial epigenomic data, we deliberately leave the choice of data scale to the user so that the model is best fit to the modality of interest.

3.5 Incorporating spatial priors and losses into complex deconvolution models

The models implemented above are selected primarily for their simplicity that makes them suitable for a wide range of data modalities and also reduces the computational burden (few seconds to minutes on a personal laptop for a typical 10x Visium sample). However, because of the modular design, we may also integrate the spatial loss into more sophisticated models. Below we outline two examples:

3.5.1 A generative hierarchical Bayesian model with spatial prior

The assumption that different cell types distribute independently is defective as information cannot be shared across cell types. Cell2location[20] is a generative hierarchical model that introduces an additional layer of

variables to share common abundance profiles among co-localized cell types. Here we follow the Cell2location model and demonstrate how to incorporate spatial priors into the framework.

Under the mixture model, the spatial transcriptomics data is generated by a negative binomial distribution. For simplicity, we assume that only one spatial dataset $Y_{G \times S}$ is considered and the reference expression signature $R_{G \times C}$ is provided. We further ignore priors of distribution parameters as they are unaffected by the proposed spatial prior. This leads to

$$\begin{aligned} y_{gs} \sim& NB(\mu_{gc}, \alpha_g) \\ \mu_{gs} =& \gamma_s(\zeta_g \sum_c r_{gc} x_{cs} + \eta_g) \end{aligned}$$

where γ_s is the spot-specific scaling factor, ζ_g and η_g are the gene-specific multiplicative and additive scaling factors, and α_g is the gene-specific over-dispersion parameter.

The linear dependencies (across cell types) of cell-type abundance is modeled by the following Gamma prior with mean υ^x and fixed rate δ^x that controls the strength of the spatial prior:

$$\begin{aligned} x_{cs} \sim& Gamma(\upsilon^x_{cs}\delta^x, \delta^x) \\ \upsilon^x_{cs} =& \sum_k d_{ck} z_{ks} \\ z_{ks} \sim& Gamma(\upsilon^z_k \delta^z, \delta^z) \end{aligned}$$

where d_{ck} is the contribution of latent spatial distribution pattern (i.e. a group of co-localized cell types) k to the abundance of cell type c, and z_{ks} is the total abundance of spatial pattern k at spot s controlled by a Gamma prior with mean υ^z and rate δ^z. To incorporate spatial dependencies across spots, simply replace the original Gamma prior on z_{ks} with a multivariate Gaussian prior:

$$z_{k.} \sim MVN(\upsilon^z_k, \Sigma_k)$$

3.5.2 A deep-learning-based model with spatial prior

Classic linear models might not work well since $R_{G \times C}$ itself is also a random variable, and the relationship between R and Y can deviate from perfect linearity as noise and technical artifacts prevail. Deep learning models are especially powerful for modeling nonlinear relationships between variables. As shown in Scaden[21], a deep neural network that takes bulk gene counts as input and outputs cell-type compositions, it's possible to learn noise-free latent representations of cell types even if the training data is artificially generated by the naive aggregation of randomly sampled single cells.

During the training phase, Scaden tries to minimize the prediction loss for every pair of normalized pseudo-bulk gene expression profile Y and the ground truth cell-type composition $\hat{X}$:

$$L_1(Y, \hat{X}) = \|M_\theta(Y) - \hat{X}\|_1$$

where M_θ is an ensemble of three four-layer dense neural networks with dropout regularization.

With the ability to simulate realistic spatial omics data with distinct patterns and varying degree of noise (Supplementary Figure. 5), training a similar deep-learning-based model for spatial omics data is straightforward. We may simply add the proposed spatial loss to the objective function and optimize model parameters on simulated spatial data

$$\tilde{\theta} = \underset{\theta}{\operatorname{argmin}} \|M_\theta(Y_{G \times S}) - \hat{X}_{C \times S}\| + \lambda \sum_c (M_\theta(Y_{G \times S})_{c:}) \Sigma_c^{-1} M_\theta(Y_{G \times S})'_{c:}.$$

4 Dimensionality reduction with spatial priors and losses

4.1 Dimensionality reduction, reference-free deconvolution, and matrix factorization

Most linear dimensionality reduction methods, including non-negative matrix factorization (NMF), principal component analysis (PCA), independent component analysis (ICA), and linear discriminant analysis (LDA), are generalizations of the basic matrix factorization

$$Y_{G\times S} = W_{G\times H} Z_{H\times S} \tag{13}$$

where G is the size of the original space and $H \ll G$ is the size of the latent reduced space. The apparent analogy between the above problem to deconvolution eq. (6) suggests that cell types may be viewed as latent factors. When the reference expression signature $X_{G\times C}$ ($i.e. W_{G\times H}$ above) is unknown, factorizing $Y_{G\times S}$ is both a dimensionality reduction task and a reference-free deconvolution task. This translates into the following formulation:

$$\tilde{W}, \tilde{Z} = \underset{W,Z}{\operatorname{argmin}}\, L_M(Y, W, Z) + L_W(W) + L_Z(Z) \tag{14}$$

where W is the estimated reference signature of latent variables (or cell types), Z is the contribution of latent variables (or cell-type abundance) across space, L_M is any measure on the quality of lower representations, and L_W and L_Z are the losses that impose soft constraints W and Z. In the special case where we expect isotropic Gaussian noise, a flat prior on W and a zero-centered spatial smoothing prior on Z

$$\begin{aligned} y_{gs} &\sim \mathcal{N}(\sum_h w_{gh} z_{hs}, \delta^2) \\ z_{h.} &\sim MVN(0, \Sigma_h) \end{aligned}$$

we can write the maximum a posteriori (MAP) estimation of the solution as

$$\begin{aligned} \tilde{W}_{MAP}, \tilde{Z}_{MAP} &= \underset{W,Z}{\operatorname{argmax}}\, P(W, Z|Y) \\ &= \underset{W,Z}{\operatorname{argmin}}\, \|Y_{G\times S} - W_{G\times H} Z_{H\times S}\|_2^2 + \lambda \sum_h Z_{h.} \Sigma_h^{-1} Z'_{h.} \end{aligned} \tag{15}$$

Note that while the above is similar to eq. (8), it is non-convex as W is unknown and is optimized jointly with Z. Still, practical solutions can be found via iterative algorithms.

4.2 Autoencoders with spatial losses

Autoencoder (AE) is a class of neural network in which an encoder network is trained to learn an efficient low-dimensional representation of the input, and a decoder network is trained to reconstruct the input from the latent representation. In this section we present several examples of AE models with the proposed spatial losses following the previous formulation eq. (14).

4.2.1 PCA with spatial losses

Often regarded as one of the simplest AE, PCA uses the same linear layer in its encoder and decoder and minimizes the L2 reconstruction loss with orthogonality constraints. More specifically,

$$\tilde{W} = \underset{W}{\operatorname{argmin}}\, \frac{1}{GS} \|Y_{G\times S} - W_{G\times H} Z(W, Y)_{H\times S}\|_2^2 + \frac{\lambda}{HS} \sum_h Z(W, Y)_{h.} \Sigma_h^{-1} Z(W, Y)'_{h.} \tag{16}$$

where $Z(W,Y)_{H\times S} = W'Y$ is the latent representation and $W_{G\times H}$ is constrained to be column-orthogonal. We solve the above problem via gradient descent and orthogonality is restored at the end of each iteration by the orthogonal (Gram-Schmidt) projection.

4.2.2 Linear and multi-layer autoencoders with spatial losses

Next, we seek to introduce spatial losses to more sophisticated AE models. We first loosen the symmetry and orthogonality constraints of PCA, which leads to the following linear AE model

$$\hat{Y}_{G\times S} = W^2_{G\times H}(W^1_{H\times G}Y_{G\times S})$$

and its solution is

$$\begin{aligned}
\tilde{W^1}, \tilde{W^2} &= \underset{W^1,W^2}{\operatorname{argmin}} \frac{1}{GS}\|Y - W^2(W^1Y)\|_2^2 + \lambda_{orth}L_{orth} + \frac{\lambda_{sp}}{HS}L_{sp} \\
L_{orth} &= \|(\hat{W^1Y})(\hat{W^1Y})' - I_{H\times H}\| \\
L_{sp} &= \sum_h (W^1Y)_{h.}\Sigma_h^{-1}(W^1Y)'_{h.}
\end{aligned} \tag{17}$$

where L_{orth} and L_{sp} are the soft orthogonality loss and the spatial smoothing loss imposed on the latent embedding $(W^1Y)_{H\times S}$, respectively. Here $\hat{W^1Y}$ is the column-normalized embedding so that each hidden dimension has unit norm. For more complicated models, we formulate the objective function in a similar way

$$\begin{aligned}
\tilde{\theta}, \tilde{\phi} &= \underset{\theta,\phi}{\operatorname{argmin}} \frac{1}{GS}\|Y - D_\phi(E_\theta(Y)\|_2^2 + \lambda_{orth}L_{orth} + \frac{\lambda_{sp}}{HS}L_{sp} \\
L_{orth} &= \|(E_\theta\hat{(Y)})(E_\theta\hat{(Y)})' - I_{H\times H}\| \\
L_{sp} &= \sum_h (E_\theta(Y))_{h.}\Sigma_h^{-1}(E_\theta(Y))'_{h.}
\end{aligned} \tag{18}$$

where E_θ and D_ϕ is the nonlinear encoder and decoder in a multi-layer AE model, respectively. Batch normalization is often required for deep AE models to prevent embedding collapse.eqs. (17) and (18) are implemented and solved using PyTorch.

4.2.3 Variational autoencoders with spatial losses

Variational Autoencoders (VAEs) are a type of autoencoder that adds a probabilistic twist, imposing and learning a distribution on the latent vectors. Specifically, the encoder learns to map the input data to a distribution in the latent space, while the decoder learns to reconstruct the original data from this latent distribution. The encoder and decoder are trained together using a combination of reconstruction loss (like traditional AEs) and a KL divergence term that measures how much the learned latent distribution deviates from a target prior distribution (usually a standard Gaussian). When incorporating spatial losses into VAEs, by default we enforce spatial consistency on distribution means in the latent representation. This would lead to the following form:

$$\begin{aligned}
\tilde{\theta}, \tilde{\phi} &= \underset{\theta,\phi}{\operatorname{argmin}} L_{recon}(Y, D_\phi(Z)) + \lambda_{KL}D_{KL}(q_\theta(Z|Y)||p(Z)) + \lambda_{sp}L_{sp} \\
L_{sp} &= \sum_h (E[q_\theta(Z|Y)])_{h.}\Sigma_h^{-1}(E[q_\theta(Z|Y)])'_{h.}
\end{aligned} \tag{19}$$

where L_{recon} is the reconstruction accuracy (e.g., negative log likelihood of a negative binomial distribution), $q_\theta(Z|Y)$ is the encoding network, $p(Z)$ is the target prior distribution (often chosen as $N(0,1)$), and $D_\phi(Z)$ is the decoding network.

Like eq. (18), we implemented and solved eq. (19) using PyTorch. Specifically, we designed the SpatialVAE model to be spatially informed counterpart of the SCVI models from the package scvi-tools.

4.3 Contrastive spatial loss

While the quadratic spatial loss $L_{sp} = X_S' \Sigma^{-1} X_S$ has its natural roots in the multivariate Gaussian prior, it is a loss that rewards local similarity but not separability. For example, the CAR prior eq. (4) implies that if X is constant, then the loss is always zero regardless of the covariance structure Σ. To encourage the model to separate physically distant spots in the latent space, we propose a contrastive extension of the above spatial loss

$$L_{csp} = X_S' \Sigma_0^{-1} X_S - \frac{1}{T} \sum_t X_S' \Sigma_t^{-1} X_S := X_S' A X_S \tag{20}$$

where Σ_0 is the covariance derived from the correct spatial graph and $\{\Sigma_t, t \in [1, T]\}$ are those from corrupted spatial graphs generated by random shuffling. Note that in general $A = \Sigma_0^{-1} - \frac{1}{T} \sum_t \Sigma_t^{-1}$ is not positive semi-definite, therefore the contrastive loss can be negative with no finite lower bound.

When the strength of spatial loss (λ_{sp}) is too large, hidden embeddings generated by the encoder are likely to degenerate and collapse. This issue is more prominent in complex AE models and when the training data is sparse. Empirically, we observed that the contrastive loss increases the robustness of spatial loss to λ_{sp} by an order of magnitude.

4.4 Relationship to GNN-based autoencoders

Graph neural network (GNN) is a class of neural networks that capture data dependency via information flow along edges of the corresponding graph of the data. In spatial transcriptomics, that graph can be built from physical coordinates, gene expression, and histology of each spot. Many GNN-based models have been developed for spatial transcriptomics data to generate a better representation. SpaGCN[22] first builds a spatial graph with spots as nodes and physical and histological distances as edges, then applies a single graph convolutional layer to encode raw expression data and optimizes the model on an unsupervised iterative clustering task. STAGATE[23] is a graph attention AE with symmetric encoder and decoder where similarities between spots are learned adaptively via attention. DeepST[24] is a graph convolutional network (GCN)-based AE that performs contrastive learning by additionally minimizing mutual information between lower representations and corrupted graph structures. While these models are all powerful for spatial transcriptomics data in their individual tasks, the fact that all three methods incorporate the spatial graph as an integral part makes them incapable of joint modeling other data modality where spatial information is not available. Importantly, there is no way to transfer the rich knowledge learned from non-spatial single-cell transcriptomics atlas to guide the encoding of spatial data. By modularizing the spatial dependence as a dispensable loss term, our Smoother framework essentially fills the gap. AE models such as DCA[25] that were pre-trained on larger volume of non-spatial single-cell data can be seamlessly extended to spatial data, where it only needs fine-tuning with regard to the new spatially aware objective.

5 Data imputation and resolution enhancement with spatial priors and losses

5.1 Simple data denoising by spatial smoothing

Historically, Gaussian filters and convolutions (weighted local average) have long been used to attenuate technical noise in the data. In this section, we show how the same basic idea can be applied to spatial data

via spatial priors and losses. Here we assume the noise is zero-centered and Gaussian i.i.d., and the latent true expression of gene g at spot s has the proposed spatial prior (for convenience, also zero-centered)

$$\begin{aligned} y_{gs} &\sim \mathcal{N}(\mu_{gs}, \delta^2) \\ \mu_{g.} &\sim MVN(0, \Sigma_g). \end{aligned} \tag{21}$$

The solution is

$$\tilde{\mu}_{MAP} = \underset{\mu}{\operatorname{argmin}} \|Y_{G\times S} - \mu_{G\times S}\|_2^2 + \lambda_{sp} \sum_g \mu_{g.} \Sigma_g^{-1} \mu'_{g.}, \tag{22}$$

which is a special case of Tikhonov regularization and an explicit analytical solution exists if Σ_g^{-1} is positive semi-definite. To see the equivalence of eq. (22) to weighted-average-based smoothing, we return to the ICAR model eq. (4) where the conditional expectation of y_{gi} is given by $\frac{1}{\sum_j w_{ij}} \sum_j w_{ij} y_{gj}$, the weighted average of its neighbors. As such, the spatial loss is at the minimum if all μ_{gs} are equal to the local weighted averages (or in the trivial case when μ is the zero vector).

Again, Smoother's modular design makes it easy to adjust the strength of spatial smoothing, and the simple L2 reconstruction loss in eq. (22) can be replaced by more sophisticated model-based losses to fit the need and complexity of the data, as is discussed in section 5.3.

5.2 Imputation of missing data and resolution enhancement

When data at part of the profiled region is missing, we may recover it via an imputation scheme that also relies on neighbors. In eq. (21), unobserved spots do not directly contribute to the overall likelihood, therefore the solution of imputation is still eq. (22) except the reconstruction loss is only calculated at observed locations. Similarly, for any arbitrary set of spatial locations, we can impute the latent values by first building the spatial covariance structure a priori, then applying the above solution to jointly minimize the reconstruction loss over observed positions and the spatial smoothing loss over all positions.

5.3 Model-based denoising and imputation

Often, technical noise in the data is neither gene- nor location-independent. By carefully modeling of this variability, we may borrow information across genes and locations to improve denoising and imputation performance.

5.3.1 Deconvolution-guided denoising and imputation

The expressions of marker genes can only be independent if conditioned on cell-type abundances. Therefore, it is intuitively plausible to combine the deconvolution task and denoising/imputation task so that gene-level dependencies can be better modeled. Given the deconvolution framework eq. (5), we may use the generative model and the latent cell-type abundances estimated by a separate deconvolution model to denoise the observed data. If we further consider cell-type abundance as fixed, we can impose additional structure on the noise term to model remaining spatial variability in deconvolution residues.

$$\begin{aligned} y_{gs} &\sim \mathcal{N}(\mu_{gs}, \sigma^2) \\ \mu_{gs} &= \gamma_{gs} + \sum_c r_{gc} x_{cs} \\ \gamma_{g.} &\sim MVN(0, \Sigma_g). \end{aligned}$$

5.3.2 AE-guided denoising and imputation

An autoencoder compresses the original data by learning an efficient latent representation, and the decoder specifically is designed to reconstruct noise-free data using only essential information encoded in the latent embedding. For detailed discussion of the design and implementation of AE models, please refer to the previous section 4.2.

References

[1] Patrik L Ståhl, Fredrik Salmén, Sanja Vickovic, Anna Lundmark, José Fernández Navarro, Jens Magnusson, Stefania Giacomello, Michaela Asp, Jakub O Westholm, Mikael Huss, et al. Visualization and analysis of gene expression in tissue sections by spatial transcriptomics. *Science*, 353(6294):78–82, 2016.

[2] Yanxiang Deng, Marek Bartosovic, Petra Kukanja, Di Zhang, Yang Liu, Graham Su, Archibald Enninful, Zhiliang Bai, Gonçalo Castelo-Branco, and Rong Fan. Spatial-cut&tag: spatially resolved chromatin modification profiling at the cellular level. *Science*, 375(6581):681–686, 2022.

[3] Yanxiang Deng, Marek Bartosovic, Sai Ma, Di Zhang, Petra Kukanja, Yang Xiao, Graham Su, Yang Liu, Xiaoyu Qin, Gorazd B Rosoklija, et al. Spatial profiling of chromatin accessibility in mouse and human tissues. *Nature*, pages 1–9, 2022.

[4] Ying Ma and Xiang Zhou. Spatially informed cell-type deconvolution for spatial transcriptomics. *Nature Biotechnology*, pages 1–11, 2022.

[5] Haoran Zhang, Miranda V Hunter, Jacqueline Chou, Jeffrey F Quinn, Mingyuan Zhou, Richard White, and Wesley Tansey. Bayestme: A unified statistical framework for spatial transcriptomics. *bioRxiv*, 2022.

[6] Jay M Ver Hoef, Ephraim M Hanks, and Mevin B Hooten. On the relationship between conditional (car) and simultaneous (sar) autoregressive models. *Spatial statistics*, 25:68–85, 2018.

[7] Haluk Derin, Howard Elliott, Roberto Cristi, and Donald Geman. Bayes smoothing algorithms for segmentation of binary images modeled by markov random fields. *IEEE Transactions on Pattern Analysis and Machine Intelligence*, (6):707–720, 1984.

[8] Karsten Held, E Rota Kops, Bernd J Krause, William M Wells, Ron Kikinis, and H-W Muller-Gartner. Markov random field segmentation of brain mr images. *IEEE transactions on medical imaging*, 16(6):878–886, 1997.

[9] James Diebel and Sebastian Thrun. An application of markov random fields to range sensing. *Advances in neural information processing systems*, 18, 2005.

[10] Olivier François, Sophie Ancelet, and Gilles Guillot. Bayesian clustering using hidden markov random fields in spatial population genetics. *Genetics*, 174(2):805–816, 2006.

[11] Qian Zhu, Sheel Shah, Ruben Dries, Long Cai, and Guo-Cheng Yuan. Identification of spatially associated subpopulations by combining scrnaseq and sequential fluorescence in situ hybridization data. *Nature biotechnology*, 36(12):1183–1190, 2018.

[12] Ruben Dries, Qian Zhu, Rui Dong, Chee-Huat Linus Eng, Huipeng Li, Kan Liu, Yuntian Fu, Tianxiao Zhao, Arpan Sarkar, Feng Bao, et al. Giotto: a toolbox for integrative analysis and visualization of spatial expression data. *Genome biology*, 22(1):1–31, 2021.

[13] Edward Zhao, Matthew R Stone, Xing Ren, Jamie Guenthoer, Kimberly S Smythe, Thomas Pulliam, Stephen R Williams, Cedric R Uytingco, Sarah EB Taylor, Paul Nghiem, et al. Spatial transcriptomics at subspot resolution with bayesspace. *Nature Biotechnology*, 39(11):1375–1384, 2021.

[14] Benjamin Chidester, Tianming Zhou, Shahul Alam, and Jian Ma. Spicemix: Integrative single-cell spatial modeling of cell identity. *bioRxiv*, pages 2020–11, 2022.

[15] Wei Liu, Xu Liao, Yi Yang, Huazhen Lin, Joe Yeong, Xiang Zhou, Xingjie Shi, and Jin Liu. Joint dimension reduction and clustering analysis of single-cell rna-seq and spatial transcriptomics data. *Nucleic acids research*, 50(12):e72–e72, 2022.

[16] Rebecca Elyanow, Ron Zeira, Max Land, and Benjamin J Raphael. Starch: Copy number and clone inference from spatial transcriptomics data. *Physical Biology*, 18(3):035001, 2021.

[17] Daphne Tsoucas, Rui Dong, Haide Chen, Qian Zhu, Guoji Guo, and Guo-Cheng Yuan. Accurate estimation of cell-type composition from gene expression data. *Nature communications*, 10(1):1–9, 2019.

[18] Aaron M Newman, Chih Long Liu, Michael R Green, Andrew J Gentles, Weiguo Feng, Yue Xu, Chuong D Hoang, Maximilian Diehn, and Ash A Alizadeh. Robust enumeration of cell subsets from tissue expression profiles. *Nature methods*, 12(5):453–457, 2015.

[19] Patrick Danaher, Youngmi Kim, Brenn Nelson, Maddy Griswold, Zhi Yang, Erin Piazza, and Joseph M Beechem. Advances in mixed cell deconvolution enable quantification of cell types in spatial transcriptomic data. *Nature communications*, 13(1):1–13, 2022.

[20] Vitalii Kleshchevnikov, Artem Shmatko, Emma Dann, Alexander Aivazidis, Hamish W King, Tong Li, Rasa Elmentaite, Artem Lomakin, Veronika Kedlian, Adam Gayoso, et al. Cell2location maps fine-grained cell types in spatial transcriptomics. *Nature biotechnology*, 40(5):661–671, 2022.

[21] Kevin Menden, Mohamed Marouf, Sergio Oller, Anupriya Dalmia, Daniel Sumner Magruder, Karin Kloiber, Peter Heutink, and Stefan Bonn. Deep learning–based cell composition analysis from tissue expression profiles. *Science advances*, 6(30):eaba2619, 2020.

[22] Jian Hu, Xiangjie Li, Kyle Coleman, Amelia Schroeder, Nan Ma, David J Irwin, Edward B Lee, Russell T Shinohara, and Mingyao Li. Spagcn: Integrating gene expression, spatial location and histology to identify spatial domains and spatially variable genes by graph convolutional network. *Nature methods*, 18(11):1342–1351, 2021.

[23] Kangning Dong and Shihua Zhang. Deciphering spatial domains from spatially resolved transcriptomics with an adaptive graph attention auto-encoder. *Nature communications*, 13(1):1–12, 2022.

[24] Yahui Long, Kok Siong Ang, Mengwei Li, Kian Long Kelvin Chong, Raman Sethi, Chengwei Zhong, Hang Xu, Zhiwei Ong, Karishma Sachaphibulkij, Ao Chen, et al. Deepst: A versatile graph contrastive learning framework for spatially informed clustering, integration, and deconvolution of spatial transcriptomics. *bioRxiv*, 2022.

[25] Gökcen Eraslan, Lukas M Simon, Maria Mircea, Nikola S Mueller, and Fabian J Theis. Single-cell rna-seq denoising using a deep count autoencoder. *Nature communications*, 10(1):1–14, 2019.
