# Supplementary material for: Smoother: a unified and modular framework for incorporating structural dependency in spatial omics data
Source: Genome Biol. 2023 Dec 18;24:291. doi: 10.1186/s13059-023-03138-x (PMC10726548; doi:10.1186/s13059-023-03138-x)
Supplement: Supplementary file 3 — Additional file 3. Review history. [file 13059_2023_3138_MOESM3_ESM.docx]

Review History

**First round of review**

**Reviewer 1**

**Were you able to assess all statistics in the manuscript, including the appropriateness of statistical tests used?**

No.

**Were you able to directly test the methods?**

No.

**Comments to author:**

In this manuscript, Su et al. proposed Smoother, a unified modular framework to model spatial dependencies in spatial omics data. Through the introduction of spatial priors and losses, Smoother can be readily integrated into existing non-spatial models to deal with different analytic tasks in spatial omics, including data imputation, cell-type deconvolution, and dimensionality reduction. The authors demonstrated the superiority of incorporating spatial dependencies in the above tasks by applying Smoother on simulated and real spatial data.

Overall, the study provided a universal solution to modeling and incorporating spatial dependencies, which is compatible with a variety of algorithms that are not specifically designed for spatial analyses, and facilitates their applications on spatial omics data. However, given that there are already many spatially-aware algorithms that were developed for different tasks, such as STAGATE, GraphST for cell type deconvolution, Sprod, and MIST for data imputation. The reviewer has concerns about the advantages of Smoother over other spatially aware methods that were designed for specific tasks. The authors should compare the performance of Smoother-guided analysis with other spatially aware methods, as done in the task of cell-type deconvolution, and emphasize the superiority of Smoother. I have the following comments.

Major comments:

1. The authors introduced four methods in the supplementary note to model spatial random processes, including SMA, SAR, CAR, and ICAR. How do the four models perform in different tasks and in different spatial techniques or omics? The authors should conduct more analyses to show their performance and give more detailed advice about the choice of these models.

2. In the applications of cell-type deconvolution and dimensionality reduction, the authors compared the performance of models with and without spatial priors. However, in the application of data imputation, the authors only showed the performance of Smoother and didn't compare it with imputation without spatial priors or other published spatially aware imputation methods, such as Sprod, MIST, STAGATE, and XFUSE. The authors should add more analyses to demonstrate the necessity of modeling spatial dependencies in spatial data imputation and show the advantages of Smoother over other spatially aware imputation methods.

3. The authors implemented variants of PCA and general linear auto-encoders (LAE) by leveraging spatial context and applied them to the DLPFC dataset. As shown in Extended data Fig. 2, neither PCA nor LAE did very well in separating spatial spots from different cortical layers. I'm wondering whether performing UMAP following spatially aware PCA or LAE would improve the visualization. Or could it be helpful to add spatial loss in deep learning-based auto-encoders? The authors should provide more convincing examples to show the superior performance of spatially aware dimensionality reduction by Smoother over other methods, such as STAGATE, SEDR, and SpaceFlow.

4. Though the authors mentioned that the tuning of spatial strength relied on cross-validation and empirical Bayes approaches in 'Discussion and conclusion', is it possible for Smoother to choose the spatial strength automatically when users have no prior knowledge?

Minor comments:

1. The figure legends should be more detailed to help users understand the figures. For example, in the simulated doughnut-shaped or embryo ST dataset, Fig 1b-i shows the deconvolution performance.

1) Fig 1b shows the relative abundance of cell types with high or low densities. Does the abundance plot only show one representative cell type or the aggregated cell-type abundance of high or low densities?

2) Do the bar plots in Fig 1 show the deconvolution accuracy of one cell type or the overall performance of all cell types?

3) In Fig 1d and 1h, which color represents the presence, yellow or purple?

4) As described in the legend of Fig 1d and 1h, the presence of one cell type is defined by 'proportion >= 0.05', which is inconsistent with the description in the method (abundance >= 0.1). Please confirm it.

2. Fig 2a visualizes the deconvolution results of Smoother and CARD, but it's difficult to distinguish the tumor region represented by black circles from the darker region in the CD3 immunofluorescence staining image. Also, with the cell-type proportion pie plot covering the staining image, we cannot easily read the CD3 intensity in the same position. The authors should provide more readable figures to visualize T cell proportions inferred by Smoother and CARD and compare them with CD3 immunofluorescence intensity.

3. In the spatial-CUT&Tag H3K4me3 data, since 37 embryonic cell types from scRNA-seq are inseparable in the UMAP plot, is it reasonable to infer the proportions of such small cell types? Will it impact the deconvolution accuracy?

4. Similar chartreuse regions near the blue regions were identified in both NNLS (+ spatial loss) and CARD (Fig 2i), but the lineage of cardiac muscle is only detected in NNLS (+ spatial loss) (Fig 2j). Are there any explanations for these results?

**Reviewer 2**

**Were you able to assess all statistics in the manuscript, including the appropriateness of statistical tests used?**

Yes: The reported statistics are relatively straightforward.

**Were you able to directly test the methods?**

No.

**Comments to author:**

In this manuscript, Su and colleagues presented Smoother, a modular model to integrate spatial information into transcriptomic data for spatial transcriptomics data analysis. The authors demonstrated the numerical improvement, which is incremental to this reviewer. Moreover, there are also several concerns that should be addressed.

Major concern:

1) The approach (and to a large extent, most other approaches claiming to integrating the spatial information in analysis) explicitly assumes that spatially adjacent spots tend to derive from a similar cluster. Although this approach may perform better in processing data derived with clear anatomy such as normal brain, there are equally abundant (and likely to be much more) samples derived from malignancy (to understand the spatial intertumoral heterogeneity as well as tumor-stromal interaction). Compared to the normal tissue, tumor samples are generally less organized and often have small patch of tumor subpopulations surrounded by different stromal component (or small patch of immune cells infiltrate into the malignant tissues). Will the proposed approach over-smooth the analysis and generate sub-optimal result? Specifically, Fig 2b showed a performance of T cell infiltration in a cancer dataset. Although Smoother achieved the highest performance among the methods in the benchmark study, its Spearman correlation around 0.3 essentially suggested that all methods (including the proposed Smoother) failed in this test and cannot be applied to cancer spatial transcriptomics datasets with confidence.

2) Spatial transcriptomics data often suffer from signal bleeding from adjacent spots (https://www.nature.com/articles/s41467-022-30587-y, https://www.sciencedirect.com/science/article/pii/S167202292200167X). Does the proposed approach handle this major concern?

3) Compared to commonly used 10X Visium platform, some spatial transcriptomic platforms (such as Seq-scope and Stereo-seq) have significantly larger scale of spots profiled in each experiment (also suffer from an increasing data sparcity issue). How are the computational efficiency and robustness of the proposed Smoother approach in these different setting?

4) Is there any major finding that other existing algorithm can't reveal?. If so, any experimental validation?

Minor concerns:

1) Why did the author use cosine similarity for PCA projection of gene expression where the features are supposed to be orthogonal and the scale of PC is representing the variance explained? On the other side, they used Euclidean distance to measure the similarity in histology.

**Authors Response**

**Point-by-point responses to the reviewers’ comments:**

**Reviewer #1:**

In this manuscript, Su et al. proposed Smoother, a unified modular framework to model spatial dependencies in spatial omics data. Through the introduction of spatial priors and losses, Smoother can be readily integrated into existing non-spatial models to deal with different analytic tasks in spatial omics, including data imputation, cell-type deconvolution, and dimensionality reduction. The authors demonstrated the superiority of incorporating spatial dependencies in the above tasks by applying Smoother on simulated and real spatial data.

Overall, the study provided a universal solution to modeling and incorporating spatial dependencies, which is compatible with a variety of algorithms that are not specifically designed for spatial analyses, and facilitates their applications on spatial omics data. However, given that there are already many spatially-aware algorithms that were developed for different tasks, such as STAGATE, GraphST for cell type deconvolution, Sprod, and MIST for data imputation. The reviewer has concerns about the advantages of Smoother over other spatially aware methods that were designed for specific tasks. The authors should compare the performance of Smoother-guided analysis with other spatially aware methods, as done in the task of cell-type deconvolution, and emphasize the superiority of Smoother. I have the following comments.

*Reply:*

- *Thank you for taking the time to review our manuscript and for providing valuable feedback. We are glad to hear that you found our proposed framework, Smoother, to be a potentially universal solution for modeling spatial dependencies. We understand your concern regarding the advantages of Smoother over other spatially aware methods that were designed specifically for certain tasks. To address this, we have performed additional comparisons to highlight the superiority of Smoother in the task of cell-type deconvolution (as requested by Reviewer #2) and dimensionality reduction with a focus on new biological discoveries.*
- *Specifically, we compared Smoother-guided deconvolution against SPOTlight and CARD on a new colorectal cancer Stereo-seq dataset (new Figure 5). The results show that Smoother identified clinically relevant, literature-confirmed localization patterns of three plasma cell subpopulations, which were entirely missed by SPOTlight in the original Zhang et al., Fundamental Research 2022 and CARD. A detailed analysis can be found below in our responses to Reviewer #2’s major comments 1 and 4.*
- *Furthermore, following your suggestion, we have incorporated spatial loss into other deep variational auto-encoders (SCVI and SCANVI) and generated for the first time spatially aware joint embeddings of both spatial and non-spatial data of human prostate. This enhancement led to a substantial improvement in reference mapping accuracy (new Figure 6). Notably, our results also correct a mistake in Hirz et al., Nature Communications 2023, where smooth muscle cells were mistakenly classified as fibroblasts. To the best of our knowledge, no other existing spatially aware dimensionality reduction methods can be applied to non-spatial data (scRNA-seq atlas) and therefore cannot be used to transfer cell-type annotations. A detailed analysis can be found below in the response to your major comment 3.*

Major comments:

1.      The authors introduced four methods in the supplementary note to model spatial random processes, including SMA, SAR, CAR, and ICAR. How do the four models perform in different tasks and in different spatial techniques or omics? The authors should conduct more analyses to show their performance and give more detailed advice about the choice of these models.

*Reply:*

- *We apologize for not providing sufficient details about the differences of spatial processes in the original manuscript. We have included detailed advice on model selection in the ‘Methods - Construction of spatial priors and loss’ subsection.*
- *The spatial random processes, SAR and CAR, are mathematically equivalent with appropriate transformation (see:* [*https://mhooten.github.io/publications/VerHoef_etal_SpatStat_2018.pdf*](https://mhooten.github.io/publications/VerHoef_etal_SpatStat_2018.pdf)*). We included both models to offer users the flexibility to choose their preferred parameterization. This is analogous to choosing between the mean-dispersion and the size-prob parameterizations of the negative distribution. Additionally, we also introduced ICAR and ISAR as the weights-scaled versions of CAR and SAR. This is for convenience so that the spatial autocorrelation hyperparameter ‘rho’ falls in [0, 1], which controls the positive definiteness of the resulting covariance. We always recommend using ICAR and ISAR unless otherwise necessary. In contrast to the other processes, SMA is a ‘local’ model that incorporates spatial dependency up to second-order neighbors (neighbors’ neighbors) of each spot.*
- *To illustrate the covariance structure imposed by different models, we have visualized the correlation decay between neighbors (see below, Supplementary Fig. 3a-c). Notably, the raw covariance without graph pruning is homogenous, and after scaling, the pairwise correlation varies even for neighboring pairs of the same distance (shadows above and below each line).*

***Supplementary Figure 3: Covariance structure analysis under different spatial random processes and autocorrelation parameters.*** *(a-c) Distribution of pairwise correlation between the k-th nearest neighbors, as specified by the spatial stochastic process (columns) and the autocorrelation parameter (rows) ‘rho’. Intuitively, the autocorrelation parameter ‘rho’ can be viewed as the proportion of information that comes from the neighbors. The distributions are shown with colored lines and the associated gray areas indicating the median and 25%-75% range of correlation, respectively, over all neighboring pairs of the same neighborhood degree k (self-correlation of 1 when k=0). Color denotes different strategies to construct the spatial weights matrix. The spatial weights in the neighborhood graph are further scaled by expression similarity in (b), and by histology similarity in (c).*

-*Empirically, the specific choice of the spatial process does not have a significant impact as long as the final covariance structure is similar. That is, an ICAR model with a smaller ‘rho’ will yield the same effect as a more local SMA model. In practice, we recommend using ICAR with varying ‘rho’s (or ISAR with smaller ‘rho’s) to accommodate data with different dependencies. For example, ‘rho=0.99’ for data with clear anatomy and ‘rho=0.9’ for tumor data. This is based on pure numeric considerations, as the inverse covariance matrix of the SMA tends to be less sparse, potentially slowing down computation. On the other hand, the ISAR model's inverse covariance matrix can become improper when 'rho' is close to one. While this is acceptable for loss function usage, it may lead to issues in interpretation, such as dramatic increases in spot variance due to numeric instability in matrix inverse operations (see below, Supplementary Fig. 3g).*

***Supplementary Figure 3: Covariance structure analysis under different spatial random processes and autocorrelation parameters. …*** *distributions of per-spot variances are shown in (g-i).*

2.      In the applications of cell-type deconvolution and dimensionality reduction, the authors compared the performance of models with and without spatial priors. However, in the application of data imputation, the authors only showed the performance of Smoother and didn't compare it with imputation without spatial priors or other published spatially aware imputation methods, such as Sprod, MIST, STAGATE, and XFUSE. The authors should add more analyses to demonstrate the necessity of modeling spatial dependencies in spatial data imputation and show the advantages of Smoother over other spatially aware imputation methods.

*Reply:*

- *We again apologize for any confusion caused by insufficient comparisons and the terminology used. The imputation task we refer to is closer to the concept of kernel smoothing in computer vision where the input can be any spatial variable, not necessarily expression data. We have changed the name of* *the module from “data imputation” to “feature smoothing” in the new Figure 1 to better reflect the difference.*

- *We develop the Smoother-guided imputation to be an ultra-fast versatile functionality for users who want to smooth or enhance the resolution of any spatial data, especially non-expression data such as protein abundances (See Figure 2b in* [*https://www.nature.com/articles/s41587-022-01536-3*](https://www.nature.com/articles/s41587-022-01536-3)*) and cell-type density (See the cancer dataset analysis in* [*https://www.nature.com/articles/s41592-022-01575-3#Sec18*](https://www.nature.com/articles/s41592-022-01575-3#Sec18)*: “the myeloid cell type density covariate was calculated by convolving the cell type locations, weighted by UMI number, with an exponential filter”). It is important to emphasize that our imputation model deliberately uses information no more than the neighborhood structure (i.e., no cross-gene information borrowing, no histology image) so that the method can be applied to any single spatial feature of interest.*

**Table 1: Conceptual differences between imputation methods**

|  | Smoother | Sprod | MIST | STAGATE | XFUSE |
| --- | --- | --- | --- | --- | --- |
| Non-counts data | x | x | x | x |  |
| Single feature | x | x |  |  |  |
| Resolution enhancement | x |  |  |  | Requires histology image |
| Arbitrary data distribution | x |  |  |  |  |
| Speed | <10 secs for ~40,000 spots* |  |  |  |  |

*CPU only, 16G RAM M1 MacBook Air

- *As shown in the Table 1 above, most existing imputation methods like MIST (low-rank approximation), STAGATE (latent representation), and XFUSE (metagene) cannot work with single feature, since they require information borrowing across genes. XFUSE also requires additional histological image data and is count-specific since it uses negative binomial distribution to model the data. On the other hand, Sprod’s imputation step is mathematically the same as the Smoother-guided imputation model with MSE loss and, since the problem is convex, the solution is also the same if given the same neighboring graph. However, MSE loss implies that the feature is intrinsically Gaussian, which might not be the case with other type of spatial data (e.g., compositional data is on a simplex). The modular design of* *Smoother allows users to combine spatial loss with any reconstruction loss suitable for their applications, while low-level optimizations are handled by the smoother package.*
- *Furthermore, among published methods compared in the above Table 1, Smoother-guided imputation is the only one that can achieve histology-free resolution enhancing. We provide an example (see below the new Supplementary Fig. 5) where region signature scores calculated on 10x Visium data were augmented to arbitrary resolutions (in <1 secs). The accuracy of our resolution enhancing relies mainly on the underlying assumption that spatially adjacent spots share more similar characteristics. Since there is no noise-free super-resolved spatial transcriptomics technologies available to generate ground truth, we unfortunately cannot provide more quantitative benchmark. We have included this limitation in the corresponding Results section.*

***Supplementary Figure 5: Resolution enhancement of marker gene expression in the DLPFC dataset (151673).*** *The figure depicts the enhancing of the spatial resolution of gene signature scores in the DLPFC slide 151673. The 10x Visium original slide was cropped (first column) to visualize the enhancement at different resolutions. Gene signature scores were defined per region using ‘scanpy.tl.score_genes’ as the weighted scaled average of top20 marker genes of that region (adjusted for gene background).*

- *While our method is unique in many technical aspects, we recognize the need to showcase its practical utility. In the context of single feature imputation, the baseline model without spatial priors is filling missing values with the global average, which is clearly inadequate when the feature exhibits certain spatial patterns. To demonstrate the biological meaningfulness of the Smoother-guided imputation method, we applied it to Slide-seqV2 data to smooth and enhance the resolution of pathway activity scores (see below, Supplementary Fig. 6). These scores were defined as the average scaled expression of a gene module and were adjusted for gene background. Since genes in the same pathway are already closely connected, imputation algorithms that borrow information across similar genes may introduce biases to the statistic and artificially amplify the signal. Instead, we treated the pathway score as a holistic variable and imputed the score individually. The resulting smoothed functional scores showed enhanced pairwise correlations (upper right triangle in Supplementary Fig. 6b and d), offering improved insights into the spatial relationships among functional pathways.*

***(Next page) Supplementary Figure 6: Application of Smoother for smoothing and enhancing functional activity scores in the Slide-seqV2 MBM data.*** *The figure demonstrates the imputation (a, b) and resolution enhancement (c, d) of functional signature scores in a Slide-seqV2 melanoma brain metastasis slide (MBM11_rep2, a, b) and an extracranial melanoma metastasis slide (ECM01_rep2, c, d). Functional scores are calculated based on marker gene expression following the original publication. (a) MHC-I and ribosomal scores before and after smoothing. (b) Pairwise correlation between functional signature scores before (lower left) and after (upper right) smoothing. (c) Resolution enhancement of chemokine and interferon response scores before and after enhancement. (d) Pairwise correlation between functional signature scores before (lower left) and after (upper right) enhancement.*

3.      The authors implemented variants of PCA and general linear auto-encoders (LAE) by leveraging spatial context and applied them to the DLPFC dataset. As shown in Extended data Fig. 2, neither PCA nor LAE did very well in separating spatial spots from different cortical layers. I'm wondering whether performing UMAP following spatially aware PCA or LAE would improve the visualization. Or could it be helpful to add spatial loss in deep learning-based auto-encoders? The authors should provide more convincing examples to show the superior performance of spatially aware dimensionality reduction by Smoother over other methods, such as STAGATE, SEDR, and SpaceFlow.

*Reply:*

- *Thank you for your valuable suggestions. We have made the necessary revisions to showcase the unique strength of Smoother-guided dimensionality reduction models over other methods.*
- *We first sincerely apologize for not presenting the conceptual difference of Smoother-guided dimensionality reduction models clearly enough in the original manuscript. Existing spatially aware models including STAGATE, SEDR and SpaceFlow use hard-coded approach (e.g., graph attention) to make use of the spatial location and, in consequence, cannot be applied to non-spatial data (e.g., scRNA-seq atlas). In contrast, the Smoother framework encodes spatial information via the modular and detachable spatial loss that is not part of the encoder, making it the current only approach capable of generating a spatially aware joint embedding of both spatial and non-spatial data.*
- *To better illustrate our point, we remade the cartoon on three different embedding paradigms (new Figure 6a, see below). Here we choose scVI, a widely used variational inference toolbox for scRNA-seq data analysis (*[*https://www.nature.com/articles/s41592-018-0229-2*](https://www.nature.com/articles/s41592-018-0229-2)*;* [*https://www.nature.com/articles/s41587-021-01206-w*](https://www.nature.com/articles/s41587-021-01206-w)*), as an example. When integrating scRNA-seq datasets and transferring annotations with scVI, the conventional paradigm is to train a reference model on large atlas datasets, fine-tune on the query data, and generate joint embeddings of both reference and query for label transfer (RNA-only embeddings). Since existing spatially aware models like STAGATE, SEDR, and SpaceFlow require neighborhood structures as input, they cannot be applied to the scRNA-seq reference and hence cannot be used for reference mapping (ST-only embeddings). In our study, we propose a third paradigm where an RNA-only model is first learned from the non-spatial atlas data, then converted into a spatially aware one via fine-tuning under the spatial loss (spatially aware joint embeddings). Importantly, this paradigm is not limited to the scVI workflow. In theory, the spatial loss can be attached to any optimization-based non-spatial model, either pretrained or not, to make it spatially aware. For example, if initialized using scRNA-seq data and fine-tuned with spatial loss, the SpatialPCA model can also project spatial and non-spatial data onto a joint latent space.*

***Figure 6: Smoother enables spatially aware joint embeddings of single-cell and Slide-seqV2 data of human prostate and improves reference mapping accuracy.*** *(a) Schematic overview of the spatial conversion of non-spatial auto-encoder models in the Smoother framework. Smoother enforces spatial consistency via the detachable loss function. This allows the same model to be trained and applied to both spatial and non-spatial data, generating a joint spatially aware embedding.*

- *When applied to a human prostate dataset, SpatialVAE, the SCVI model equipped with our spatial loss, demonstrated significantly better performance compared to its RNA-only counterpart. Here our goal is to annotate healthy human prostate Slide-seqV2 slides using scRNA-seq reference from the Tabular Sapiens. We first downloaded the pretrained RNA-only model (new Figure 6b, see below) from the SCVI model hub and fine-tuned the model on the spatial data (ignoring coordinates) to remove batch effects as much as possible. While SCVI is already among the best performing integration tools (https://www.nature.com/articles/s41592-021-01336-8), the Slide-seqV2 data were still clearly separated from the rest of single-cell data in the joint RNA-only embedding space (new Figure 6d, see below), probably because of low sequencing depth (new Figure 6c, see below). As a result, most spots were annotated as ambiguous (not close to any single-cell clusters within the uncertainty threshold, new Figure 6e). On the other hand, SpatialVAE effectively mitigated batch effects (new Figure 6f, see below) and transferred cell labels with dramatically reduced ambiguity. Detailed model diagnosis revealed a tradeoff between reconstruction accuracy and the spatial consistency of the embeddings,* *suggesting that the spatial loss potentially acts via enforcing the model to focus on spatially consistent features rather than batch-specific technical noise. (new Supplementary Fig.27a, see below).*

***Figure 6: Smoother enables spatially aware joint embeddings of single-cell and Slide-seqV2 data of human prostate and improves reference mapping accuracy.*** *(b) UMAP visualization of the latent representation of scRNA-seq data of human prostate from the Tabula Sapiens, colored by tissue compartment (left) and technical batch (right). The representation was generated from a pre-trained RNA-only SCVI prostate model. (c) Violin plots showing the number of expressed gene (left) and total RNA counts (right) per cell or spot in data of different technologies. (d) UMAP visualization of the joint latent representation of the Tabula Sapiens prostate scRNA-seq reference and the Slide-seqV2 data of a healthy prostate section. Following the SCVI data integration workflow, the RNA-only model was fine-tuned on the query spatial data with unfrozen parameters to mitigate batch effect. (e) Spatial visualizations of the tissue compartment (left) and cell type prediction (right) results based on the joint RNA-only embeddings shown in (d). (f) UMAP visualization of the joint latent representation generated by SpatialVAE. The spatially aware model has the same architecture as RNA-only models in (b) and (d), except it was fine-tuned to minimize the proposed spatial loss in addition to the original reconstruction and KL losses. (g) Spatial visualizations of the tissue compartment (left) and cell-type prediction (right) results based on the joint RNA-only embeddings shown in (f).*

***Supplementary Figure 27: Spatially aware joint embedding of single-cell and spatial transcriptomics data of human prostate, related to Figure 6.*** *(a) Visualizations of the training loss of the prostate SpatialVAE model. The overall loss objective is the sum of reconstruction loss (left), spatial loss (middle, zero for RNA-only models), and the KL local loss. Starting from the reference model, we first fine-tuned the model on the Slide-seqV2 data without the spatial loss until convergence (black curve, 100 epoch), then attached the spatial loss and fine-tuned with respect to the new objective (red curve). This is mainly to highlight the tradeoff between reconstruction accuracy and spatial consistency. Skipping the RNA-only fine-tuning step will not affect the performance of the final spatial model.*

- *Using the prostate joint embeddings, we were also able to correct a previous annotation mistake in the original publication (Hirz et al., Nature Communications 2023). In their paper, Hirz et al. generated paired immune-enriched scRNA-seq data and applied RCTD to map cell types to the spatial data. While initially labeled as fibroblasts, the SpatialVAE model predicted the majority of stromal cells to be indeed smooth muscle cells (pericytes in Hirz et al., new Supplementary Fig.27b). We confirmed the finding via marker gene expression (new Supplementary Fig.27c-d).*

***Supplementary Figure 27: Spatially aware joint embedding of single-cell and spatial transcriptomics data of human prostate, related to Figure 6.*** *(b) Hirz et al. and the Tabula Sapiens use slightly different cell type nomenclatures, where the ACTA2+ population is referred to as pericytes in Hirz et al. and as smooth muscle cells in the Tabula Sapiens. (c) Expression of fibroblast (DCN+, ACTA2- in Hirz et al.) marker genes. (d) Expression of smooth muscle cell (pericytes in Hirz et al., DCN-, ACTA2+) marker genes.*

- *We also recognize the interest to apply dimensionality reduction to spatial data alone. As suggested by the reviewer, we compared the performance of the SpatialPCA against two graph neural networks, STAGATE and SpaceFlow. In general, deep learning models outperformed SpatialPCA by a large margin (See below, new Supplementary Fig. 26a-b). This is expected as the parameter space of deep* *neural nets is much larger, requiring more computational resources to fit but is also capable of generating more flexible and usually better representations. Indeed, UMAP visualizations showed that adding the spatial loss can only expand PCA embeddings up to a certain degree before collapsing, which was not sufficient for separating nuanced cortex layer structures (new Supplementary Fig. 26c).*

***Supplementary Figure 26: Comparative analyses of dimensionality reduction performance on the DLPFC dataset.*** *For each sample, we projected either log-normalized expression (PCA) or the raw counts (other models) of the top 2000 highly variable genes onto a 30-dimension latent space using each model to calculate the Silhouette score. ‘Mclust’ was used to cluster spots based on the latent embeddings into the same number of clusters (cortex layers) as observed in the sample. Adjusted rand index (ARI) was calculated based on the clustering results. (a-c) Performance comparisons of PCA models with varying strength of the regular spatial loss and the contrastive spatial loss and the two graph neural networks, STAGATE and SpaceFlow. (c) UMAP visualization of the latent representation learned by each model, colored by spot layer membership.*

- *We next sought to determine if the spatial loss could help with more flexible deep-learning-based models, as suggested by the reviewer. Specifically, we designed a baseline neural net where the two graph attention layers in STAGATE were replaced by two fully connected layers. By increasing the strength of spatial loss, we were able to recover up to 50% - 90% performance (see below new Supplementary Fig. 26d-e). The effect of spatial loss on the latent embeddings is apparent under UMAP visualization, as we see that the space gradually expands and the transitions between layers become clearer (new Supplementary Fig. 26h).*

***(Next page) Supplementary Figure 26: Comparative analyses of dimensionality reduction performance on the DLPFC dataset.*** *… (d-h) Performance comparisons of the baseline neural network with varying strength of the regular spatial loss and the contrastive spatial loss and the two graph neural networks, STAGATE and SpaceFlow. The baseline model was constructed by replacing the two graph attention layers in STAGATE with two fully connected layers of the same number of hidden units (128). (f-g) Performance gains were computed against the baseline for each slide. Each boxplot shows the median and the 25th/75th percentiles over the 12 samples and whiskers indicating the 1.5 times interquartile ranges. (h) UMAP visualization of the latent representation learned by each model, colored by spot layer membership.*

- *In conclusion, the modular nature of Smoother allows our dimensionality reduction models to generate spatially aware joint embeddings of both spatial and non-spatial data, enabling accurate reference mapping and label transferring. Additionally, when working with spatial data alone, the spatial loss can be deployed as a fast and versatile alternative strategy to enforce spatial awareness independently of model architecture, albeit with a slight performance tradeoff.*

4.      Though the authors mentioned that the tuning of spatial strength relied on cross-validation and empirical Bayes approaches in 'Discussion and conclusion', is it possible for Smoother to choose the spatial strength automatically when users have no prior knowledge?

*Reply:*

- *Thank you for the insightful comment regarding the automatic selection of spatial strength. Unfortunately, it would be challenging and even counterproductive to select the strength in a one-size-fits-all manner. Since the spatial loss is ultra-fast to compute, we instead recommend users to run multiple explorative analyses with varying strengths and select the best performing model. Our previous results also confirmed that the beneficial effect of spatial regularization is robust across a wide range of strengths (Supplementary Fig. 15-17).*
- *The spatial loss in Smoother is a generic loss term that can be attached to any objective function for optimization. As such, the strength of the regularization depends on other components of the objective in each specific scenario. For instance, when switching from MSE to likelihood-based loss when* *calculating the reconstruction error of an auto-encoder, the objective can vary significantly by orders of magnitude. Therefore, there is simply no one-size-fits-all solution that can uniformly balance spatial regularization and the non-spatial objective across all scenarios.*
- *Within a specific model, the spatial loss strength acts as a tunable regularization hyperparameter, similar to other regularization parameters commonly used in regression models (e.g., ‘C’ in Support Vector Regression as implemented in sklearn.svm.SVR). To select the most suitable strength, one typically requires a measurable performance metric (e.g., accuracy on a training set) as the optimization target, which is usually not available when working with spatial data. For example, the number of slides per experiment is usually limited, and the ground truth (cell-type labels, etc.) is usually inaccessible. It might be possible to select the strength, which scaled inversely with the variance, using maximum likelihood estimators if the model is probabilistic. But again, that procedure would be model-specific.*
- *Nevertheless, we have developed tools that allow users to learn and adjust the spatial covariance structure based on their knowledge or assumptions about the data. One informative approach involves analyzing the pairwise similarity decay curve, as demonstrated in Supplementary Fig. 1 (shown below). Normal samples with clear anatomy tend to exhibit stronger spatial similarities, whereas spots in tumor and low-depth samples tend to be less similar. While similarity should not be confused with covariance, it can still guide the selection of spatial priors where the spatial process and an autocorrelation hyperparameter ‘rho’ collectively control the covariance decay rate (Supplementary Fig. 3a-f, see below). Empirically, we observed that ‘ICAR’ with rho=0.99 works well on data with strong spatial dependencies, while ‘ICAR’ with rho=0.9 may align better with tumor samples.*

***Supplementary Figure 1: Spatially adjacent spots share similar profiles, a common property of spatial omics data observed across biological systems and technologies.*** *The figure demonstrates the distributions of pairwise cosine similarity between neighboring spots in spatial omics data. The similarity is calculated based on the first 20 PCs of log-normalized gene counts (for transcriptomics data) or gene activity scores (for epigenomics data). All transcriptomics data were preprocessed following the standard Scanpy workflow by SODB. Left in each panel: Cumulative distribution of pairwise similarity between k-nearest physically adjacent spots. Red line shows the observed distribution whereas blue line shows the null distribution of random shuffled spots. Right in each panel: Variability in pairwise similarity as a function of neighbor distance. For each neighborhood degree level k (x-axis), pairwise similarities were calculated between the k-th nearest neighbors (self-similarity of 1 when k=0). The light gray area indicates the 10%-90% range, dark gray the 25%-75% range, and the red line the median, respectively. Datasets inspected: (a) 10x Visium human brain dorsolateral prefrontal cortex (DLPFC). (b) 10x Visium human pancreatic ductal adenocarcinoma (PDAC). (c) Slide-seqV2 mouse hippocampus. (d) Slide-seqV2 human melanoma brain metastasis (MBM).*

***Supplementary Figure 3: Covariance structure analysis under different spatial random processes and autocorrelation parameters.*** *(a-c) Distribution of pairwise correlation between the k-th nearest neighbors, as specified by the spatial stochastic process (columns) and the autocorrelation parameter (rows) ‘rho’. Intuitively, the autocorrelation parameter ‘rho’ can be viewed as the proportion of information that comes from the neighbors. The distributions are shown with colored lines and the associated gray areas indicating the median and 25%-75% range of correlation, respectively, over all neighboring pairs of the same neighborhood degree k (self-correlation of 1 when k=0). Color denotes different strategies to construct the spatial weights matrix. The spatial weights in the neighborhood graph are further scaled by expression similarity in (b), and by histology similarity in (c). Estimated exponential decay rates of median correlation are depicted in (d-f), and distributions of per-spot variances are shown in (g-i).*

Minor comments:

1.      The figure legends should be more detailed to help users understand the figures. For example, in the simulated doughnut-shaped or embryo ST dataset, Fig 1b-i shows the deconvolution performance.

*Reply:*

- *We apologize for the lack of details in the previous figures and thank the reviewer for all constructive suggestions. The original Figure 1b-i are now presented in new Figure 3 showing the deconvolution performance on synthetic data. We have also added corresponding legends to improve readability.*

***Figure 3: Evaluation of spatial regularization effects on deconvolution accuracy using simulated data.***

*In the first simulation, doughnut-shaped spatial transcriptomic datasets were generated from a scRNA-seq reference on a 50x50 grid. We assigned 15 cell types of high (n=5) and low (n=10) densities to overlapping spatial compartments (a-d). In the second simulation, spatial transcriptomic datasets of the mouse embryo were generated from the sci-Space dataset by pooling single cells of eight types barcoded at the same spot (approximately 200µm pitch) (e-h). (a) Relative abundance of two typical cell types in a single replicate. (b) Deconvolution accuracy of different methods as measured by Pearson correlation, with results aggregated over ten independent replicates. Error bars denote standard errors of the mean (50 high-density and 100 low-density cell types in total). (c) Binary presence status (proportion >= 0.05) of two typical cell types in a single replicate. (d) Deconvolution accuracy of different methods, similar to (b), but measured by binary prediction average precision score. (e) Relative abundance of the lateral plate mesoderm in one sci-Space slide. (f) Deconvolution accuracy of different methods as measured by Pearson correlation, with results aggregated over 14 biological slides. Error bars denote standard errors of the mean (112 cell types in total). (g) Binary presence status (proportion >= 0.05) of the myocyte in one sci-Space slide. (d) Deconvolution accuracy of different methods, similar to (f), but measured by binary prediction average precision score. Smoother-guided deconvolution models: NNLS (nonnegative least square), DWLS (dampened weighted least squares, modified implementation), SVR (support vector regression), and LNR (log normal regression. The CARD model features its own implementation of spatial regularization.*

1)      Fig 1b shows the relative abundance of cell types with high or low densities. Does the abundance plot only show one representative cell type or the aggregated cell-type abundance of high or low densities?

*Reply:*

- *The previous panels Figure 1b, d, f, h (current Figure 3a, c, e, g, shown above) each visualizes the abundance of a single (different) cell type in one replicate/slide. Since spatial patterns were randomly assigned to different cell types in each replicate, cell types in Figure 3a and 3c have no specific* *meanings and were picked for visualization purposes (i.e., to demonstrate the smoothing effect on region boundaries). The spatial localization patterns of lateral plate mesoderm and myocytes in Figure 3e and 3g are real, except the spatial data used for deconvolution were manually synthesized and subject to sampling error.*

2)      Do the bar plots in Fig 1 show the deconvolution accuracy of one cell type or the overall performance of all cell types?

*Reply:*

- *The bar plots show the deconvolution accuracy of all cell types across all replicates. For the previous Figure 1c and 1e (current Figure 3b and 3d), it is the aggregated results of 5 highly abundant cell types and 10 rare cell types across 10 independent replicates (50 and 100 data points in total). For the previous Figure 1g and 1i (current Figure 3f and 3h), it is the aggregated results of 8 cell types over 14 slides (112 data points in total). Error bar denotes standard error of the mean.*

3)      In Fig 1d and 1h, which color represents the presence, yellow or purple?

*Reply:*

- *Yellow. We have added the corresponding legend to the new figure.*

4)      As described in the legend of Fig 1d and 1h, the presence of one cell type is defined by 'proportion >= 0.05', which is inconsistent with the description in the method (abundance >= 0.1). Please confirm it.

*Reply:*

- *We apologize for the confusion and have confirmed that ‘proportion >= 0.05’ was the threshold used to generate the plot. This threshold does not affect performance benchmark and conclusions.*

2.      Fig 2a visualizes the deconvolution results of Smoother and CARD, but it's difficult to distinguish the tumor region represented by black circles from the darker region in the CD3 immunofluorescence staining image. Also, with the cell-type proportion pie plot covering the staining image, we cannot easily read the CD3 intensity in the same position. The authors should provide more readable figures to visualize T cell proportions inferred by Smoother and CARD and compare them with CD3 immunofluorescence intensity.

*Reply:*

- *Thank you for the feedback. The previous Figure 2 is now updated to Figure 4, where the CD3 staining intensity and inferred T cell proportions are now presented in separate panels (new Figure 4a, global view of the entire slide). Additionally, we have changed the color and rearranged the previous zoom-in panels (previous Figure 2a) to the new Supplementary Fig. 18 (see below). Red circles represent tumor regions where the T cell density should be low according to the CD3 staining, and yellow circles represent tumor regions with high T cell infiltration.*

***Figure 4: Smoother enhances cell-type deconvolution performance in various spatial omics data.*** *(a-c) Detection of tumor T-cell infiltration in a 10x Visium ductal carcinoma sample. (a) Left to right: Images showing single channel CD3 immunofluorescence staining (FITC/green), T-cell proportions as estimated by Smoother-guided NNLS and CARD, respectively. (b) Spearman correlation of predicted T-cell proportions with CD3 fluorescence levels across all spots, with whiskers indicating the 95% confidence intervals. (c) Spearman correlation of predicted T-cell proportions with the expression of T cell marker genes across all spots, similar to (b).*

***Supplementary Figure 18: T-cell infiltration in the zoom-in tumor region of the ductal carcinoma section, related to Figure 4a-c.***

*Each circle denotes a tumor occupied spot based on pathological annotation. Spots were classified as CD3+ (yellow) or CD3- (red) according to a two-component Gaussian mixture model on CD3 intensity. Pie charts indicate the estimated T-cell proportions at each spot. Background shows the single channel CD3 immunofluorescence staining (FITC/green) intensity.*

3.      In the spatial-CUT&Tag H3K4me3 data, since 37 embryonic cell types from scRNA-seq are inseparable in the UMAP plot, is it reasonable to infer the proportions of such small cell types? Will it impact the deconvolution accuracy?

*Reply:*

- *We apologize for the low resolution of the previous plot. These embryonic populations are indeed distinct and clearly defined according to marker expression (current Supplementary Fig. 19b, see below). When building the reference, we down-sampled the reference data for computational efficiency, which is partly why the cluster boundary in the visualization was not as clear as in the original MOCA publication (*[*https://www.nature.com/articles/s41586-019-0969-x*](https://www.nature.com/articles/s41586-019-0969-x)*).*

***Supplementary Figure 19: T-SNE visualization of and marker expression of the scRNA-seq reference from the Mouse Organogenesis Cell Atlas (MOCA), related to Figure 4h-k.***

- *We agree with the reviewer that simultaneously deconvoluting 37 cell types is challenging, especially when the reference and the spatial data are from different modalities. However, since each embryonic cell type expresses distinct sets of marker genes and is expected to present in the spatial data, we* *believe the best practice is to sum up abundances of functionally similar cell types after deconvolution, rather than averaging out small cell types when building the reference. This is unlikely to affect deconvolution accuracy at higher levels (i.e., when inspecting performance by lineage as demonstrated in the current Figure 4j, see below). That is, even if two cell types of the same lineage are epigenetically inseparable, the sum of their effects should still be separable from the effect of other lineages. Furthermore, the spatial regularization mechanism underlying CARD and our spatial loss is widely accepted as a solution to the multicollinearity issue. In the case of inseparable cell types, the regularization will keep only one cell type in effect and shrink the abundances of others to zero, as observed in the full deconvolution results (new Supplementary Fig. 21 and 22, not listed here for space limits).*

***Figure 4: Smoother enhances cell-type deconvolution performance in various spatial omics data.*** *(h-k) Spatial mapping of embryonic cell types in the spatial-CUT&Tag H3K4me3 50µm data of mouse embryo. (j) Estimated relative abundances of five major lineages aggregated from individual cell types. Color intensities are proportional to the estimated cell-type proportion and are scaled by the same factor across methods for visualization purposes. (k) Standardized gene activity of selected marker genes.*

4.      Similar chartreuse regions near the blue regions were identified in both NNLS (+ spatial loss) and CARD (Fig 2i), but the lineage of cardiac muscle is only detected in NNLS (+ spatial loss) (Fig 2j). Are there any explanations for these results?

*Reply:*

- *Thanks for raising the issue. That chartreuse region in CARD’s result, corresponding to the embryonic heart, is an artifact of our unsupervised clustering procedure. We used PCA with scaling to transform the compositional cell-type proportion vector from the simplex space (sum to one) back to the Euclidean space. The scaling indeed amplified the differences among the CARD estimated cardiac lineage proportions, while the actual absolute abundance range is less than 0.2% (new Supplementary Fig. 22, see below). In the absolute scale, ‘NNLS (+ spatial loss)’ is the only method that predicted the cardiac muscle lineage to have non-neglectable density (new Supplementary Fig. 20a, see below). We also checked the cell type distributions in the heart and non-heart region predicted by different methods (new Supplementary Fig. 20b, see below). Apart from stromal cells that do not belong to the five* *lineages, CARD predicted that the heart was predominantly erythroid, and its spatial regularization failed to rescue the results, contrary to the effect of our spatial loss on the NNLS model.*

***Supplementary Figure 22: Per-cell-type deconvolution results of CARD (phi=0.99) in the spatial CUT&Tag dataset, related to Figure 4h-k.***

***Supplementary Figure 20: Detailed examination of deconvolution of cardiac muscle lineage in the spatial CUT&Tag dataset, related to Figure 4h-k.*** *(a) Estimated proportions of cardiac muscle lineage. (b) Distributions of estimated proportions of the five major lineages in and outside the heart region. The heart region is defined by NNLS (+ spatial loss)-estimated proportion >= 0.02. List of cell types in each lineage: ‘Neurons & neural tube’: cholinergic neurons, excitatory neurons, granule neurons, neural progenitor cells, neural tube, notochord cells, sensory neurons, inhibitory interneurons, inhibitory neuron progenitors, postmitotic premature neurons, inhibitory neurons. ‘Glia cells’: oligodendrocyte progenitors, premature oligodendrocyte, ependymal cell, Schwann cell precursor, radial glia. ‘Erythroid’: primitive erythroid lineage, definitive erythroid lineage, megakaryocytes, white blood cells. ‘Chondrocytes & osteoblasts & mesenchyme’: chondrocyte progenitors, chondrocytes & osteoblasts, osteoblasts, connective tissue progenitors, early mesenchyme, limb mesenchyme'. ‘Cardiac muscle’: cardiac muscle lineages, myocytes.*

**Reviewer #2:**

In this manuscript, Su and colleagues presented Smoother, a modular model to integrate spatial information into transcriptomic data for spatial transcriptomics data analysis. The authors demonstrated the numerical improvement, which is incremental to this reviewer. Moreover, there are also several concerns that should be addressed.

*Reply:*

- *We appreciate your feedback and remarks regarding the improvement demonstrated in the Smoother framework. We also apologize for the lack of sufficient compelling examples on important applications in the original manuscript. To address this concern and further validate the practical utility of Smoother-guided models, we have integrated substantial additional analyses and presented new biological findings that otherwise cannot be revealed by existing algorithms.*
- *We first would like to emphasize that, through the modular introduction of spatial prior, Smoother has the potential to transform existing non-spatial methods into spatially aware ones, potentially bridging the gap between single-cell and spatial data analyses. This itself is both a significant conceptual and technical advancement, since most existing spatially aware methods cannot be applied to non-spatial data. Specifically, we demonstrated the practical benefits of jointly analyzing single-cell and spatial data in the context of reference mapping (i.e., transferring annotations from the scRNA-seq atlas, new Figure 6, see below for further explanation).*
- *To address your concerns on cell-type deconvolution, we applied Smoother to a new colorectal cancer Stereo-seq dataset and discovered tumor-specific spatial patterns of three plasma cell populations (new Figure 5). We found that the IgG plasma cell localization, confirmed by recent literature, is associated with fibroblast-related extracellular matrix remodeling. In comparison, both SPOTlight, the deconvolution method used in the original publication (Zhang et al. Fundamental Research 2022), and CARD, the state-of-the-art (SOTA) method, failed to recover the patterns. A detailed analysis can be found below in our responses to your major comments 1 and 4.*
- *To address Reviewer #1’s concerns on dimensionality reduction, we extended the SOTA SCVI data integration workflow and generated the first spatially aware joint representation of both single-cell and spatial data of human prostate (new Figure 6). Smoother is currently the only spatially aware dimensionality reduction tool that can also be applied to non-spatial data. As a result, we were able to transfer cell-type annotations from Tabular Sapiens scRNA-seq data to the spatial Slide-seqV2 data with dramatically improved accuracy. Our results also correct a mistake in the original publication (Hirz et al., Nature Communications 2023) where most of the smooth muscle cells were mislabeled as fibroblasts. A detailed analysis can be found above in our responses to Reviewer #1’s major comment 3 and below to your major comment 4.*

Major concern:

1)      The approach (and to a large extent, most other approaches claiming to integrating the spatial information in analysis) explicitly assumes that spatially adjacent spots tend to derive from a similar cluster. Although this approach may perform better in processing data derived with clear anatomy such as normal brain, there are equally abundant (and likely to be much more) samples derived from malignancy (to understand the spatial intertumoral heterogeneity as well as tumor-stromal interaction). Compared to the normal tissue, tumor samples are generally less organized and often have small patch of tumor subpopulations surrounded by different stromal component (or small patch of immune cells infiltrate into the malignant tissues). Will the proposed approach over-smooth the analysis and generate sub-optimal result? Specifically, Fig 2b showed a performance of T cell infiltration in a cancer dataset. Although Smoother achieved the highest performance among the methods in the
benchmark study, its Spearman correlation around 0.3 essentially suggested that all methods (including the proposed Smoother) failed in this test and cannot be applied to cancer spatial transcriptomics datasets with confidence.

*Reply:*

- *Thank you for raising the concern regarding the spatial dependency assumption. Indeed, we have observed that the higher similarity between spatially adjacent spots is a general characteristic of spatial omics data across samples and technologies (see below, Supplementary Fig. 1). This pattern holds true even in tumor samples, where the pairwise similarity decreases faster with distance but remains above zero, indicating persistent spatial dependencies (Supplementary Fig. 1b, d, and f). In contrast, shuffling the spatial coordinates will completely remove the similarity pattern, corresponding to the null hypothesis that variables are randomly distributed across space (Supplementary Fig. 1i). Based on the null, we have confirmed that the long-range similarity pattern is not an artifact of spatial contamination (lateral diffusion or spot swapping, Supplementary Fig. 1i, see below). We have also confirmed this finding through analyses of different spatial technologies, suggesting that it is neither an artifact of technology nor of limited spatial resolution. For instance, the Stereo-seq axolotl data (Supplementary Fig. 1e) was segmented and binned into single-cell resolution, yet we observed the same similarity structure. Of the spatial technologies inspected, Slide-seqV2 tends to generate data with weaker similarity structure due to its low sequencing depth. Similar results have been reported in other studies examining image-based single-cell-resolution spatial transcriptomic data (for example, Supplementary Figs 1 and 2 in* [*https://www.nature.com/articles/s41587-022-01273-7*](https://www.nature.com/articles/s41587-022-01273-7)*).*

***Supplementary Figure 1: Spatially adjacent spots share similar profiles, a common property of spatial omics data observed across biological systems and technologies.*** *The figure demonstrates the distributions of pairwise cosine similarity between neighboring spots in spatial omics data. The similarity is calculated based on the first 20 PCs of log-normalized gene counts (for transcriptomics data) or gene activity scores (for epigenomics data). All transcriptomics data were preprocessed following the standard Scanpy workflow by SODB. Left in each panel: Cumulative distribution of pairwise similarity between k-nearest physically adjacent spots. Red line shows the observed distribution whereas blue line shows the null distribution of random shuffled spots. Right in each panel: Variability in pairwise similarity as a function of neighbor distance. For each neighborhood degree level k (x-axis), pairwise similarities were calculated between the k-th nearest neighbors (self-similarity of 1 when k=0). The light gray area indicates the 10%-90% range, dark gray the 25%-75% range, and the red line the median, respectively. Datasets inspected: (a) 10x Visium human brain dorsolateral prefrontal cortex (DLPFC). (b) 10x Visium human pancreatic ductal adenocarcinoma (PDAC). (c) Slide-seqV2 mouse hippocampus. (d) Slide-seqV2 human melanoma brain metastasis (MBM). (e) Stereo-seq axolotl brain (segmented and binned at single-cell resolution). (f) Stereo-seq human colorectal cancer (CRC, binned into 50 µm x 50 µm spots). (g) Spatial-CUT&Tag mouse embryo, H3K4me3 20µm. (h) Spatial-CUT&Tag mouse embryo, H3K4me3 50µm. (i) Shuffled Stereo-seq single-cell resolution data. Per contamination rate p, each spot contains 1-p of its original RNA counts and p of RNA counts from its adjacent neighbors.*

-*We agree with the reviewer that tumor samples are generally less organized. In fact, as shown in the distributions of pairwise similarities, not all neighbors look alike even in normal samples (see above Supplementary Fig. 1, dark and light gray areas). To address the potential risk of over-smoothing and to preserve region boundaries, we have deliberately designed mechanisms that control spatial interactions (new Figure 2, see below), which is absent in CARD and many other spatial methods. Building on spatial priors, Smoother provides a more interpretable and gentler approach to imposes dependencies compared to black box graph neural networks. In particular, Bayesian theory guarantees that the prior assumption can be overcome when information in the data is strong enough. The user can also completely block unwanted interactions between two regions (new Figure 2b, see below) given any prior annotations (new Figure 2d). By default. we use soft pruning by either histology or expression similarity to scale down the interaction between less similar physical neighbors. However, the actual boundary might not be as sharp as expected. For example, while pathological regions are clearly separated in the H&E staining in the breast cancer 10x Visium data (new Figure 2c), both expression and histology data suggest moderate similarities across region boundaries as well as heterogeneity within each region (new Figure 2d and 2e, see below).*

***Figure 2: Construction of boundary-preserving spatial priors.*** *(a) Schematic illustration of the process for constructing spatial weights and covariance matrices. Smoother first builds a distance-based spatial neighborhood graph, which is subsequently pruned using histology or other features to encode boundary information and inhibit undesired cross-region interactions. (b) The impact of hard pruning on the covariance structure. Given the regional membership annotation, hard pruning sets the weights between adjacent spots of different regions to zero (teal, top) or a nominal value (leak=0.01, yellow, bottom), generating distinctive correlation distributions for boundary and interior pairs of spots. (c) An example section (1160920F) from the human breast cancer datasets. Left to right: H&E staining, manual pathological annotation, and depiction of boundary and interior spots as defined by the pathological regions. (d) Unsupervised clustering results of the same slide in (c). Spots were clustered based on gene expression (left), or image-based features extracted from the H&E staining image (right). (e) The distribution of pairwise feature similarities between boundary pairs from two regions (orange) and interior pairs from the same region (blue), as defined in (c) by the pathological annotation. (f) The distribution of pairwise correlations imposed by the resulting spatial priors after graph pruning. Left: Soft scaling using expression- (top) and histology-based similarities (bottom). Right: Hard pruning using cluster memberships from expression (top) and histology (bottom) as defined in (d).*

- *Regarding the example of T cell infiltration (previous Figure 2a-c, current Figure 4a-c, see below), we apologize for the inadequate visualization and demonstration of deconvolution performance. Indeed, the relative low correlation between CD3 protein staining intensity and inferred T cell proportions is caused primarily by the imperfect capturing efficiency and sampling uncertainty of spatial transcriptomics technologies. For example, the correlation is even lower when comparing CD3 protein staining to the expression of T cell marker genes, including the CD3 genes themselves (new Figure 4b-c, see below). Qualitatively, Smoother-guided deconvolution models captured the overall spatial pattern of T cells and its infiltration into the tumor region with decent accuracy, while CARD failed the test (see below the new Figure 4a and yellow circles in the current Supplementary Fig. 18).*

***Figure 4: Smoother enhances cell-type deconvolution performance in various spatial omics data.*** *(a-c) Detection of tumor T-cell infiltration in a 10x Visium ductal carcinoma sample. (a) Left to right: Images showing single channel CD3 immunofluorescence staining (FITC/green), T-cell proportions as estimated by Smoother-guided NNLS and CARD, respectively. (b) Spearman correlation of predicted T-cell proportions with CD3 fluorescence levels across all spots, with whiskers indicating the 95% confidence intervals. (c) Spearman correlation of predicted T-cell proportions with the expression of T cell marker genes across all spots, similar to (b).*

***Supplementary Figure 18: T-cell infiltration in the zoom-in tumor region of the ductal carcinoma section, related to Figure 4a-c.***

*Each circle denotes a tumor occupied spot based on pathological annotation. Spots were classified as CD3+ (yellow) or CD3- (red) according to a two-component Gaussian mixture model on CD3 intensity. Pie charts indicate the estimated T-cell proportions at each spot. Background shows the single channel CD3 immunofluorescence staining (FITC/green) intensity.*

- *To further demonstrate the utility of Smoother-guided deconvolution on cancer spatial transcriptomics datasets in the context of immune infiltration, we have conducted additional analyses on a new Stereo-seq colorectal cancer (CRC) dataset (new Figure 5, see below in the response to your major comment 4). Here we would like to highlight the following results: (1) We re-discovered three tumor-associated plasma cell subpopulations (two in lesion, one in mucosa) and their spatial infiltration patterns, which was entirely missed in the original publication (Zhang et al. Fundamental Research 2022). (2) The clinical relevance of our finding has been reported in recent literature on breast cancer, CRC, and ulcerative colitis. (3) We observed differential colocalization of epithelial and fibroblast subtypes with IgG+ and IgA+ plasma cells, suggesting intensive remodeling in the extracellular matrix in the tumor microenvironment. The comprehensive presentation of all results in this analysis can be found below in our response to your major comment 4 and in the ‘Results - Smoother-guided deconvolution unveils distinct localizations of plasma cells and fibroblasts in mismatch repair-proficient colorectal cancer’ section.*

2)      Spatial transcriptomics data often suffer from signal bleeding from adjacent spots (<https://nam02.safelinks.protection.outlook.com/?url=https%3A%2F%2Fwww.nature.com%2Farticles%2Fs41467-022-30587-y&data=05%7C01%7Crr2579%40cumc.columbia.edu%7Ca6a5fabe4fe94fa6f59208db5aa01c59%7Cb0002a9b0017404d97dc3d3bab09be81%7C0%7C0%7C638203415965856135%7CUnknown%7CTWFpbGZsb3d8eyJWIjoiMC4wLjAwMDAiLCJQIjoiV2luMzIiLCJBTiI6Ik1haWwiLCJXVCI6Mn0%3D%7C3000%7C%7C%7C&sdata=uZFSs90%2F71%2BedP7jaKu2a5VJudA1jrVc6AhRfUUakIQ%3D&reserved=0>, [https://nam02.safelinks.protection.outlook.com/?url=https%3A%2F%2Fwww.sciencedirect.com%2Fscience%2Farticle%2Fpii%2FS167202292200167X&data=05%7C01%7Crr2579%40cumc.columbia.edu%7Ca6a5fabe4fe94fa6f59208db5aa01c59%7Cb0002a9b0017404d97dc3d3bab09be81%7C0%7C0%7C638203415965856135%7CUnknown%7CTWFpbGZsb3d8eyJWIjoiMC4wLjAwMDAiLCJQIjoiV2luMzIiLCJBTiI6Ik1haWwiLCJXVCI6Mn0%3D%7C3000%7C%7C%7C&sdata=G2RQ9tSSR6lt9NAnvU1OJlMfvUQGAhj4%2FC1ZzxWDv3E%3D&reserved=0)](https://nam02.safelinks.protection.outlook.com/?url=https%3A%2F%2Fwww.sciencedirect.com%2Fscience%2Farticle%2Fpii%2FS167202292200167X&data=05%7C01%7Cjs5756%40cumc.columbia.edu%7Cc31778ed06ab4d2f564c08db5ab5f5c7%7Cb0002a9b0017404d97dc3d3bab09be81%7C0%7C0%7C638203509785387615%7CUnknown%7CTWFpbGZsb3d8eyJWIjoiMC4wLjAwMDAiLCJQIjoiV2luMzIiLCJBTiI6Ik1haWwiLCJXVCI6Mn0%3D%7C3000%7C%7C%7C&sdata=EmZcn4ErzplDX94fV9Bko6WAcf7cc5genVPovg1smlc%3D&reserved=0). Does the proposed approach handle this major concern?

*Reply:*

- *Thank you for bringing up the concern about signal bleeding.*
-*We first would like to again clarify that the spatial similarity observed in spatial transcriptomics data is not an artifact of signal bleeding. Even if allowing up to 50% of UMIs to come from adjacent spots (as done in the data simulation workflow, see below Supplementary Fig. 1i), the pairwise similarity between spatially adjacent spots still rapidly drops to zero within the second-order neighborhood (neighbors' neighbors). Moreover, we have observed similar spatial similarity patterns across spatial omics technologies, including spatial CUT&TAG, where spatial barcodes (instead of genetic materials that could cause signal bleeding) are flowed over the tissue surface through microchannels.*

***Supplementary Figure 1: Spatially adjacent spots share similar profiles, a common property of spatial omics data observed across biological systems and technologies. …*** *(i) Shuffled Stereo-seq single-cell resolution data. Per contamination rate p, each spot contains 1-p of its original RNA counts and p of RNA counts from its adjacent neighbors.*

- *During the benchmarking of deconvolution performance, we did incorporate the bleeding problem into the data simulation workflow, which we referred to as “lateral diffusion” (see below Supplementary Fig. 7a). Specifically, columns in Supplementary Fig. 8-13 correspond to different bleeding rates, where a value of 0.5 indicates that 50% of the UMIs per spot are expected to come from adjacent spots. It is worth noting that the bleeding only affects the observed spatial data, and not the ground truth cell type abundances. Our results confirm that the benefit of having the spatial loss in deconvolution is robust against the presence of signal bleeding.*

***Supplementary Figure 7: Simulating spatial transcriptomics data for deconvolution benchmark.*** *(a) The simulation pipeline, based on cell2location, introduces key modifications to assign arbitrary spatial patterns to zones (co-localized cell type groups) and incorporate additional noise (termed lateral diffusion in this paper, also called contamination, spot swapping or bleeding in literature) to account for cell-type-independent dependencies.*

***Supplementary Figure 10: Evaluation of deconvolution accuracy on simulated data under different scenarios measured by Pearson correlation (the higher the better), related to Figure 3a-d.*** *Doughnut-shaped spatial transcriptomics datasets, with varying degree of lateral diffusion (columns, number indicates the proportion of mRNA shared with adjacent spots), were generated from scRNA-seq reference on a 50x50 grid. In each of the ten experiments (replicates), we assigned 5 major cell types with high average abundances (a) and 10 minor cell types with low abundances (b) to overlapping spatial compartments.*

3)      Compared to commonly used 10X Visium platform, some spatial transcriptomic platforms (such as Seq-scope and Stereo-seq) have significantly larger scale of spots profiled in each experiment (also suffer from an increasing data sparcity issue). How are the computational efficiency and robustness of the proposed Smoother approach in these different setting?

*Reply:*

- *Thank you for raising the concern regarding computational efficiency. We are pleased to show that the Smoother framework is designed with efficiency and scalability in mind. By leveraging the sparsity of the inverse covariance structure, the computational complexity of the spatial loss (naively) is O(k*n^2), where n is the number of spots and k is the number of spatial variables of interest (e.g., the number of cell types in deconvolution). In comparison, other methods that implement a similar spatial regularization, such as CARD, have a higher complexity of at least O(k*n^3). Additionally, Smoother utilizes the powerful backend optimization support of PyTorch (and cvxpy for convex problems), which is connected to a wide range of acceleration functionalities. Together, the additional computational burden from the spatial loss in practice is typically neglectable.*
- *Smoother-guided deconvolution models are by far the most time- and memory-efficient deconvolution models. Without any GPU support, the deconvolution of a large colorectal cancer Stereo-seq data (~18, 000 spots) was completed in just 17 seconds on a personal laptop (M1 16G RAM MacBook Air). This demonstrates the suitability of Smoother for efficient data exploration, particularly for large-scale spatial data, as pointed out by the reviewer.*
- *For dimensionality reduction, the computational bottleneck is mainly the baseline non-spatial model. In the case of SpatialVAE (the SCVI model with spatial loss), training an RNA-only SCVI model on ~30,000 Slide-seqV2 spots takes about 10 mins (CPU only, M1 16G RAM MacBook Air), while the spatially aware version added no extra perceptible running time.*
- *We agree that the data sparsity issue is a serious concern for super-resolved spatial data analysis, and we believe Smoother can be part of the solution. For instance, when we applied the Smoother-guided SpatialVAE model to a human prostate dataset, the spatial loss effectively reduced the impact of low sequencing depth of Slide-seqV2 in the joint RNA-only embedding space (new Figure 6). As a result, we achieved much lower ambiguity rate in transferring cell type labels from the Tabula Sapiens scRNA-seq reference to the spatial data. Detailed description and interpretation of results in this analysis can be found in our response to Reviewer #1’s major comment 3 above and in the response to your major comment 4 below.*

4)      Is there any major finding that other existing algorithm can't reveal?. If so, any experimental validation?

*Reply:*

- *Yes. We have since added two comprehensive case studies (new Figure 5 and 6) in addition to the existing three deconvolution applications (previous Figure 2, current Figure 4) to fully address the concerns from you and the Reviewer #1.*
- *We first sincerely apologize for not presenting biologically relevant findings clearly enough in the previous manuscript. In the T-cell infiltration analysis (previous Figure 2a-c, current Figure 4a-c, see above in the response to your major comment 1), Smoother-guided deconvolution revealed the presence of T cells in tumor regions as confirmed by CD3 staining, while CARD failed. In the Spatial-CUT&Tag analysis (previous Figure 2h-k, current Figure 4h-k, see below), the incorporation of spatial loss rescued deconvolution performance, revealing clear embryonic compartments (current Figure 4j) that were confirmed by marker gene activity (current Figure 4k). Epigenomics-based activity scores are significantly different from gene expression in scale, variability, and biological meanings. Notably, we demonstrated here for the first time that cross-modality cell-type deconvolution is feasible for spatial omics data. CARD again failed to reveal the tissue compartment structure.*

***Figure 4: Smoother enhances cell-type deconvolution performance in various spatial omics data.*** *(h-k) Spatial mapping of embryonic cell types in the spatial-CUT&Tag H3K4me3 50µm data of mouse embryo. (h) Left: Overview of the spatial-CUT&Tag technology. Right: T-SNE visualization of the scRNA-seq reference of 37 embryonic cell types. (i) Embryonic subregions identified by clustering based on estimated cell-type compositions. (j) Estimated relative abundances of five major lineages aggregated from individual cell types. Color intensities are proportional to the estimated cell-type proportion and are scaled by the same factor across methods for visualization purposes. (k) Standardized gene activity of selected marker genes.*

- *To further demonstrate the utility of Smoother-guided deconvolution on large scale cancer spatial transcriptomics datasets, we applied the method to a new Stereo-seq mismatch repair-proficient (MMRp) colorectal cancer (CRC) dataset and discovered three distinct plasma cell subpopulations with differing spatial distributions (new Figure 5, see below). The original publication (Zhang et al. Fundamental Research 2022) generated collections of Stereo-seq slides of CRC and adjacent normal tissues as well as a paired scRNA-seq reference, but it fell short of in-depth analysis to examine immune infiltration.*
- *We first re-annotated the paired single-cell reference data based on gene modules reported in another comprehensive study on CRC (*[*https://doi.org/10.1016/j.cell.2021.08.003*](https://doi.org/10.1016/j.cell.2021.08.003)*) and found three single-cell plasma subpopulations, namely IgG+, IgA+, and IgA+ FOS/JUN+ (new Figure 5a and b, see below). FOS and JUN are B-cell receptor pathway modules and have been shown to be upregulated in tumor-infiltrating B cells in other solid tumors (*[*https://doi.org/10.1158/0008-5472.CAN-20-3773*](https://doi.org/10.1158/0008-5472.CAN-20-3773)*). In the original paper, Zhang et al. reported two plasma populations (‘Plasma_IGKC’ and ‘Plasma_IGLC’) without clear cell type markers, which we now believe to be mainly batch effects (see Fig 3a in* [*https://doi.org/10.1016/j.fmre.2022.01.036*](https://doi.org/10.1016/j.fmre.2022.01.036)*. The two populations are indeed separable by sample donors, and most differentially expressed genes are mitochondrial and ribosomal genes).*

***Figure 5: Smoother detects tumor-specific plasma cell subtypes in colorectal adenocarcinoma Stereo-seq slide***

***(a)*** *UMAP representation of the cell types from the colorectal adenocarcinoma scRNA-seq reference used in the original publication (REF).* ***(b)*** *Expression dotplot of the top marker genes for each cell type in the scRNA-seq samples.*

- *Using Smoother-guided deconvolution, we observed that the three plasma populations uncovered in the scRNA-seq data reside in distinct spatial regions: specifically, the IgG+ population were predominantly in the tumor region (lesion) whereas the IgA+ cells were in mucosa (new Figure 5c and 5e). We further confirmed the differential localization of IgG+ and IgA+ plasma cells using marker gene expression (new Figure 5f and Supplementary Figure 23, see below). In addition, the lesion IgG+ and mucosa IgA+ populations exhibited divergent B-cell receptor V/C gene usage, with IGLC2, IGLC3, IGLV3-1 and IGKV4-1 all enriched in the tumor, suggesting a difference in the adaptive response between the two plasma cell types (new Figure 5f). Gene Ontology pathway analysis also highlighted this difference, as “B-cell activation” was enriched in the IgG+ plasma cell sections and “Defense response to bacterium” in the IgA+ plasma cell sections (new Figure 5g). This is consistent with recent reports that IgG+ plasma cells are enriched in tumor samples and that the isoform skewing from IgA to IgG is a clinically important characteristic of CRC (*[*https://doi.org/10.1016/j.cell.2021.08.003*](https://doi.org/10.1016/j.cell.2021.08.003)*;* [*https://doi.org/10.1016/j.isci.2023.106140*](https://doi.org/10.1016/j.isci.2023.106140)*). In contrast, CARD incorrectly predicted all plasma cell clones to be IgG+, including in the mucosa region (Supplementary Fig. 23c, omitted here for space limits).*

***Figure 5: Smoother detects tumor-specific plasma cell subtypes in colorectal adenocarcinoma Stereo-seq slide***

***(c)*** *Pathology annotation of the histology slide, adapted from (REF).* ***(d)*** *Slide subregions identified by clustering based on estimated cell-type compositions. Arrows emphasize the mucosa delineation apparent with Smoother but not CARD.* ***(e)*** *Spatial visualizations of the cell-type proportions of the three plasma cell subtypes.* ***(f)*** *B-cell receptor gene expression heatmap for the IgA plasma cell spots in the mucosa and the IgG plasma cell voxels in the lesion, normalized across spots.* ***(g)*** *Colocalization of each cell type with the IgA and IgG plasma cells.* ***(h)*** *Gene Ontology pathway enrichment analysis of the IgA plasma cell and IgG plasma cell spots.*

- *Additionally, the deconvolution with Smoother revealed three phenotypically and spatially distinct fibroblast populations, characterized by previously derived ADAMDEC1+/CCL8+, CXCL14+ and matrix transcriptional programs (*[*https://doi.org/10.1016/j.cell.2021.08.003*](https://doi.org/10.1016/j.cell.2021.08.003)*), and validated by marker gene expression (Supplementary Figure 24, see below). ADAMDEC1+/CCL8+ and CXCL14+ fibroblasts were preferentially co-localized with the IgG+ plasma cells (new Figure 5h), in accordance with observed CXCL14+ cancer-associated fibroblast and ADAMDEC1+/CCL8+ fibroblast presence in MMRp CRC (*[*https://doi.org/10.1016/j.cell.2021.08.003*](https://doi.org/10.1016/j.cell.2021.08.003)*). Recent work has demonstrated ADAMDEC1+ driven fibroblastic matrix remodeling in response to inflammation* *(*[*https://doi.org/10.1371/journal.pbio.3001532*](https://doi.org/10.1371/journal.pbio.3001532)*), which explains in part the enrichment of collagen and extracellular matrix-related pathways in IgG+ plasma cell sections (new Figure 5g). On the other hand, CARD failed to separate these three fibroblast populations (Supplementary Fig. 24, see below). In the original publication, Zhang et al. also incorrectly predicted that CD4 T cells infiltrate pervasively into the tumor regions using SPOTlight for deconvolution (see Fig 3c in* [*https://doi.org/10.1016/j.fmre.2022.01.036*](https://doi.org/10.1016/j.fmre.2022.01.036)*), which was not corroborated by our Smoother results or marker gene expression and which is in contradiction with the low T-cell infiltration observed in MMRp tumors (*[*https://doi.org/10.1016/j.cell.2021.08.003*](https://doi.org/10.1016/j.cell.2021.08.003)*).*

***Supplementary Figure 24: Comparison between fibroblast subtype deconvolution results and marker gene expression in the CRC tumor tissue Stereo-seq section, related to Figure 5. (a-b)*** *Fibroblast subtype proportions as predicted by Smoother-guided DWLS* ***(a)*** *and CARD* ***(b)*** *with spatial loss.* ***(c)*** *Expression dotplot of three marker genes for each fibroblast subtype in the reference scRNA-seq samples, which correspond to previously derived ADAMDEC1+/CCL8+, CXCL14+ and matrix transcriptional programs (Pelka et al.).* ***(d)*** *Spatial expression of the subtype-specific markers shown in* ***(c)****.*

- *Regarding dimensionality reduction, our Smoother framework is the current only spatially aware approach that can be jointly applied to both spatial and non-spatial data (e.g. scRNA-seq data). The cartoon attached below (new Figure 6a) illustrates the conceptual differences between existing single-cell-based RNA models (RNA-only), existing spatially aware models (ST-only), and our approach. In brief, we propose a new data integration paradigm where an RNA-only model is first learned from the non-spatial atlas data, then converted into a spatially aware one via fine-tuning under the spatial loss (spatially aware joint embeddings). The joint embeddings can then be used for downstream tasks, including annotation transfer as demonstrated in the new Figure 6b-g.*

***Figure 6: Smoother enables spatially aware joint embeddings of single-cell and Slide-seqV2 data of human prostate and improves reference mapping accuracy.*** *(a) Schematic overview of the spatial conversion of non-spatial auto-encoder models in the Smoother framework. Smoother enforces spatial consistency via the detachable loss function. This allows the same model to be trained and applied to both spatial and non-spatial data, generating a joint spatially aware embedding.*

- *When applied to a human prostate dataset, SpatialVAE, the SCVI model equipped with our spatial loss, enabled the cell-type annotation of Slide-seqV2 slides (10µm, near single-cell resolution) with dramatically reduced ambiguity compared to its RNA-only counterpart. While SCVI is already among the best performing integration tools (https://www.nature.com/articles/s41592-021-01336-8), the Slide-seqV2 data were still clearly separated from the rest of single-cell data from the Tabula Sapiens in the joint RNA-only embedding space (new Figure 6d, see below), probably because of low sequencing depth (new Figure 6c, see below). As a result, most spots were annotated as ambiguous (not close to any single-cell clusters within the uncertainty threshold, new Figure 6e). On the other hand, SpatialVAE effectively mitigated batch effects (new Figure 6f, see below) and subsequently transferred cell labels with much lower ambiguity rate. Using the prostate joint embeddings, we were also able to correct a previous annotation mistake in the original publication (Hirz et al., Nature Communications 2023). In their paper, Hirz et al. generated paired immune-enriched scRNA-seq data and applied RCTD to map cell types to the spatial data. While initially labeled as fibroblasts, the SpatialVAE model predicted the majority of stromal cells to be indeed smooth muscle cells (pericytes in Hirz et al., new Supplementary Fig.27b). We confirmed the finding via marker gene expression (new Supplementary Fig.27c-d).*

***Figure 6: Smoother enables spatially aware joint embeddings of single-cell and Slide-seqV2 data of human prostate and improves reference mapping accuracy.*** *(b) UMAP visualization of the latent representation of scRNA-seq data of human prostate from the Tabula Sapiens, colored by tissue compartment (left) and technical batch (right). The representation was generated from a pre-trained RNA-only SCVI prostate model. (c) Violin plots showing the number of expressed gene (left) and total RNA counts (right) per cell or spot in data of different technologies. (d) UMAP visualization of the joint latent representation of the Tabula Sapiens prostate scRNA-seq reference and the Slide-seqV2 data of a healthy prostate section. Following the SCVI data integration workflow, the RNA-only model was fine-tuned on the query spatial data with unfrozen parameters to mitigate batch effect. (e) Spatial visualizations of the tissue compartment (left) and cell type prediction (right) results based on the joint RNA-only embeddings shown in (d). (f) UMAP visualization of the joint latent representation generated by SpatialVAE. The spatially aware model has the same architecture as RNA-only models in (b) and (d), except it was fine-tuned to minimize the proposed spatial loss in addition to the original reconstruction and KL losses. (g) Spatial visualizations of the tissue compartment (left) and cell-type prediction (right) results based on the joint RNA-only embeddings shown in (f).*

***Supplementary Figure 27: Spatially aware joint embedding of single-cell and spatial transcriptomics data of human prostate, related to Figure 6.*** *(b) Hirz et al. and the Tabula Sapiens use slightly different cell type nomenclatures, where the ACTA2+ population is referred to as pericytes in Hirz et al. and as smooth muscle cells in the Tabula Sapiens. (c) Expression of fibroblast (DCN+, ACTA2- in Hirz et al.) marker genes. (d) Expression of smooth muscle cell (pericytes in Hirz et al., DCN-, ACTA2+) marker genes.*

Minor concerns:

1)      Why did the author use cosine similarity for PCA projection of gene expression where the features are supposed to be orthogonal and the scale of PC is representing the variance explained? On the other side, they used Euclidean distance to measure the similarity in histology.

*Reply:*

- *Thank you for your question. In soft graph pruning and weights scaling, we chose cosine similarity as the default mainly for convenience. This is because the metric needs to be within the range of [0, 1] so that the weights matrix is still legitimate after scaling (where the binary edge weight in the neighboring graph is multiplied by the feature similarity between the two spots). With cosine similarity, we only need to set all negative values to 0, meaning no interaction, to ensure nonnegativity. In contrast, to convert the Euclidean distance into a similarity score of range [0,1], one needs to tune the bandwidth (or characteristic length scale) of the exponential kernel based on the scale of the feature, which is less convenient.*
- *On the other hand, we agree that calculating cosine similarity on PCA-reduced data may seem redundant since the resulting similarity (X.T@X) is the low-rank approximation of the cosine similarity of the original (zero-centered and scaled) data. Empirically, we found that zero-centering and scaling expression data were helpful in maximizing the dissimilarity between disparate neighbors (see below in Supplementary Fig. 2a - left, the peak near zero), which is important for effectively distinguishing between different regions. If the kernel scale of the Euclidean distance is properly selected, it too can maximize the dissimilarity (bw=0.1 in this case, Supplementary Fig. 2a – right, see below).*
- *Other than convenience, the specific choice of scaling metrics is somewhat arbitrary. For example, by default the histological feature is the concatenated RGB values of pixels covered per spot, followed by PCA. Alternatively, it can also be the averaged RGB values across all covered pixels per spot. Regardless, the resulting similarity score distributions look similar (Supplementary Fig. 2b and c, see below). Overall, we observed that scaling choices tend to have a limited impact on the final accuracy in applications. This is because scaling (and the graph) acts indirectly via the spatial prior, which becomes irrelevant if the data is strong enough. Also, spots are connected via both direct (subject to the similarity scaling) and indirect interactions (i.e., through neighbors’ neighbors). Soft scaling, unlike hard pruning, are less efficient in removing indirect interactions.*

***Supplementary Figure 2: Distributions of pairwise similarities between boundary pairs from two regions and interior pairs from the same region, related to Figure 2e.*** *Boundary pairs from two regions (orange) and interior pairs from the same region (blue) are defined in Figure 2c by the pathological annotation. (a) Transcriptomic similarities are calculated based on the log-normalized expression data of the top 2000 highly variable genes. PCA with zero-centering and scaling was applied to reduce the feature to the first 10 PCs. Left two panels: Cosine similarity. Right two panels: Euclidean similarity as converted from the Euclidean distance using an exponential kernel of bandwidth 0.01. (b) Similar to (a), except the feature is the concatenated RGB values of pixels of the H&E staining covered by the spot. PCA with zero-centering and scaling was applied to reduce the feature to the first 10 PCs. (c) Similar to (b), except the feature is the RGB values averaged over pixels of the H&E staining covered by the spot.*

**Second round of review**

**Reviewer 1**

The authors have made substantial improvements in response to the previous feedback. They successfully implemented Smoother on a Stereo-seq dataset of colorectal cancer, and discovered spatial cell-localization patterns which other tools failed to uncover. Additionally, they designed SpatialVAE, a spatial extension of SCVI, to enable spatially aware joint representation of both single-cell and spatial data. Furthermore, the manuscript has been thoughtfully restructured, and the main figures have been reorganized to enhance overall readability. In summary, the majority of the concerns have been satisfactorily addressed, leading to a significant enhancement in the manuscript's clarity and quality.

The authors have significantly strengthened their manuscript by incorporating additional supplementary figures that elucidate the rationale behind the assumption that spatially adjacent spots exhibit higher expression similarity. To underscore the efficacy of Smoother, they extended its application to a colorectal cancer Stereo-seq dataset, revealing biologically relevant insights that other existing algorithms can’t reveal. Overall, the reorganization of the manuscript and figures has effectively addressed concerns raised by Reviewer #2. I do have a few minor comments and suggestions, which I will outline below:

1. The authors modified the original Extended Figure 1 to the new Figure 2; however, there is limited discussion of it in the manuscript. I recommend the authors provide more detailed text to elucidate the construction of boundary-preserving spatial priors and the difference between soft and hard pruning sets. Alternatively, consider moving Figure 2 to the supplementary figures.

2. The legends of Figure 5g-h do not correspond accurately to the respective figure panels. Please correct this discrepancy.

3. To highlight the computational efficiency of Smoother, the authors could consider adding supplementary figures or tables depicting the time taken for different tasks across various datasets with differing numbers of spots.

**Authors Response**

**Point-by-point responses to the reviewers’ comments:**

Reviewer #1:

The authors have made substantial improvements in response to the previous feedback. They successfully implemented Smoother on a Stereo-seq dataset of colorectal cancer, and discovered spatial cell-localization patterns which other tools failed to uncover. Additionally, they designed SpatialVAE, a spatial extension of SCVI, to enable spatially aware joint representation of both single-cell and spatial data. Furthermore, the manuscript has been thoughtfully restructured, and the main figures have been reorganized to enhance overall readability. In summary, the majority of the concerns have been satisfactorily addressed, leading to a significant enhancement in the manuscript's clarity and quality.

The authors have significantly strengthened their manuscript by incorporating additional supplementary figures that elucidate the rationale behind the assumption that spatially adjacent spots exhibit higher expression similarity. To underscore the efficacy of Smoother, they extended its application to a colorectal cancer Stereo-seq dataset, revealing biologically relevant insights that other existing algorithms can’t reveal. Overall, the reorganization of the manuscript and figures has effectively addressed concerns raised by Reviewer #2. I do have a few minor comments and suggestions, which I will outline below:

1. The authors modified the original Extended Figure 1 to the new Figure 2; however, there is limited discussion of it in the manuscript. I recommend the authors provide more detailed text to elucidate the construction of boundary-preserving spatial priors and the difference between soft and hard pruning sets. Alternatively, consider moving Figure 2 to the supplementary figures.

*Reply: Thank you for the suggestion. The previous Figure 2 is now part of the new supplementary Fig S2.*

2. The legends of Figure 5g-h do not correspond accurately to the respective figure panels. Please correct this discrepancy.

*Reply: Thanks for pointing that out. We have corrected the mistake.*

3. To highlight the computational efficiency of Smoother, the authors could consider adding supplementary figures or tables depicting the time taken for different tasks across various datasets with differing numbers of spots.

*Reply: Thanks for the suggestion. We have now provide Supplementary Table S1 to show the runtime of different tasks under the Smoother framework across different datasets.*
